# ddClone: joint statistical inference of clonal populations from single cell and bulk tumour sequencing data

**DOI:** 10.1186/s13059-017-1169-3

**Published:** 2017-03-01

**Authors:** Sohrab Salehi, Adi Steif, Andrew Roth, Samuel Aparicio, Alexandre Bouchard-Côté, Sohrab P. Shah

**Affiliations:** 1 Bioinformatics Graduate Program, University of British Columbia, 570 West 7th Avenue, Vancouver, V5Z 4S6 BC Canada; 2 Department of Pathology and Laboratory Medicine, University of British Columbia, Vancouver, V6T 2B5 BC Canada; 3 Department of Molecular Oncology, British Columbia Cancer Agency, 675 West 10th Avenue, Vancouver, V5Z 1L3 BC Canada; 4 Department of Statistics, University of British Columbia, 2207 Main Mall, Vancouver, V6T 1Z4 BC Canada

**Keywords:** Intra-tumour heterogeneity, Clonal evolution, Joint probabilistic model, Distance dependent, Chinese restaurant process, Single cell sequencing, Next-generation sequencing

## Abstract

Next-generation sequencing (NGS) of bulk tumour tissue can identify constituent cell populations in cancers and measure their abundance. This requires computational deconvolution of allelic counts from somatic mutations, which may be incapable of fully resolving the underlying population structure. Single cell sequencing (SCS) is a more direct method, although its replacement of NGS is impeded by technical noise and sampling limitations. We propose ddClone, which analytically integrates NGS and SCS data, leveraging their complementary attributes through joint statistical inference. We show on real and simulated datasets that ddClone produces more accurate results than can be achieved by either method alone.

**Electronic supplementary material:**

The online version of this article (doi:10.1186/s13059-017-1169-3) contains supplementary material, which is available to authorized users.

## Background

Human cancers develop through branched evolutionary processes [1] resulting in genetically diverse clonal cell populations. Every cancer cell likely harbours a distinct genome through accrual of individual mutations; however, evolutionary relationships between cells can be hierarchically encoded with phylogenetic trees. The major clades represent cell populations with a majority shared genotype. Mutations impacting phenotypic variation between clonal populations are thought to drive the clonal population dynamics of a cancer over temporal and microenvironmental dimensions. Clonal dynamics in turn impact clinical trajectories, underpinning disease complications such as treatment resistance and metastasis.

Quantitative characterization of the number of clones, their genotypes, and their abundance is of central importance in the study of the evolutionary dynamics of cancer. Ideally, the identified clones would correspond with the branches of an underlying generative process modelled by a phylogenetic tree. In practice, because of limitations of current sequencing technologies, we are not able to directly observe clones of interest. Instead, indirect experimental methods are used: bulk targeted deep sequencing [2] and single cell sequencing [3]. In both bulk and single cell, we focus the discussion on nucleotide variant markers (single nucleotide variants, SNVs), which we assume have been identified in a preliminary analysis [4–7]. In both experimental platforms, technical challenges remain which prevent accurate inference of the desired quantities. We posited that joint statistical modelling of bulk and single cell sequencing data could improve inference of clonal composition and abundance.

We begin the discussion with an overview of methods for bulk sequencing. Bulk methods can only provide a direct measure of sampled allele prevalences (the fraction of reads that harbour a mutation at a specific genomic locus) over DNA fragments sampled from a large, mixed pool of alleles extracted from the totality of cells present in the input tissues. Consequently, allele prevalence is a compound measure impacted by the unknown quantity of non-malignant cells and the unknown composition of the constituent malignant clones. Leveraging many mutations measured from the same allelic pool, computational methods have been developed to estimate subclonal structure from allele prevalences. The PyClone model [2] takes into account several confounding factors, including statistical variation coming from the sampling of the reads; non-malignant cell fraction; mis-called bases and other technical artefacts; and most importantly, how copy number alterations resulting from segmental aneuploidies locally and/or globally deviate from diploidy. PyClone and other methods such as PhyloSub [8], Clomial [9], AncesTree [10], and SciClone [11] generally assume mutations with shared prevalence (either cellular prevalence or allele prevalence) are more likely to be co-occurring within the same cell, thus defining components of a clonal genotype. This assumption may be violated to varying degrees; mutations may be present at similar allele prevalence but distributed across clones [12].

A potential solution to this problem lies in single cell sequencing (SCS). SCS via whole genome shotgun or multiplex targeted design by PCR amplification theoretically yields direct ascertainment of genotypes whereby the data itself will encode whether sets of mutations are co-occurring in individual cells. While the measurements of SCS are conceptually simpler, they come with a much higher level of technical noise [13–16]. Since the amount of measured DNA from each cell is minimal, missing one or both of the alleles (allelic drop-out (ADO) [15]) is common, resulting in sparse representation of underlying genotypes. While missing both alleles is relatively easy to detect, missing only one can seriously skew interpretation of heterozygous loci [17]. Moreover, by construction, SCS methods sample a dramatically smaller number of cells compared to bulk sequencing. As a consequence, when estimating cellular prevalences the sampling error will tend to be markedly higher (see Results section and also Additional file 1). A number of computational methods have been developed to work with SCS data that account for (some of) these limitations. The single cell genotyper (SCG) [16] uses a hierarchical Bayesian model to cluster single cells into clones and infer constituting genotypes and their prevalences, and it models various technical errors, including doublets. Using mutual SNV patterns in the single cells, OncoNEM [18] and BitPhylogeny [19] infer the evolutionary relationships between constituent clones, while SCITE [20] also reconstructs order of mutations.

We propose to leverage the strengths of both sequencing methods for optimal computational inference of clonal genotypes and prevalences. We present a novel probabilistic model based on non-parametric Bayesian integration of bulk and single cell data. We demonstrate on synthetic and real datasets how simultaneous analysis results in improved inference of salient quantities of interest for biological inference of clonal dynamics in cancer.

## Results and discussion

**Fig. 1:**
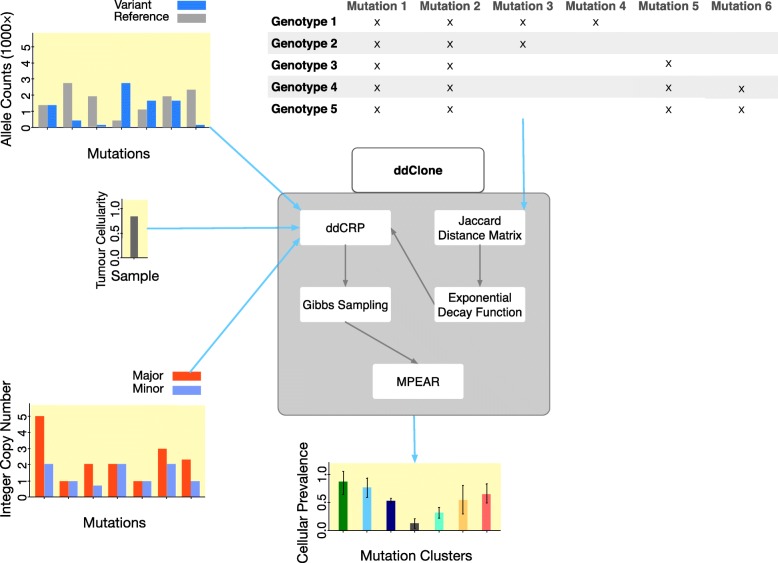
The workflow of ddClone. This figure shows the workflow of our method, ddClone. The ddClone approach is predicated on the notion that single cell sequencing data will inform and improve clustering of allele fractions derived from bulk sequencing data in a joint statistical model. ddClone combines a Bayesian non-parametric prior informed by single cell data with a likelihood model based on bulk sequencing data to infer clonal population architecture. Intuitively, the prior encourages genomic loci with co-occurring mutations in single cells to cluster together. Using a cell-locus binary matrix from single cell sequencing, ddClone computes a distance matrix between mutations using the Jaccard distance with exponential decay. This matrix is then used as a prior for inference over mutation clusters and their prevalences from deeply sequenced bulk data in a distance-dependent Chinese restaurant process framework. The output of the model is the most probable set of clonal genotypes present and the prevalence of each genotype in the population

We developed a statistical framework, ddClone, leveraging data obtained from both single cell and bulk sequencing methods (Fig. 1). The ddClone approach assumes single cell sequencing data will inform and improve clustering of allele fractions derived from bulk sequencing data in a joint statistical model. ddClone combines a Bayesian non-parametric prior informed by single cell data with a likelihood model based on bulk sequencing data to infer clonal population architecture through clustered mutations. Intuitively, the prior ‘encourages’ genomic loci with co-occurring mutations in single cells to cluster together. Using a cell-locus binary matrix from single cell sequencing, ddClone computes a distance matrix between mutations using the Jaccard distance with exponential decay. This matrix is then used as a prior for inference over mutation clusters and their prevalences from deeply sequenced bulk data in a distance-dependent Chinese restaurant process [21] framework. The output of the model is the most probable set of clonal genotypes present and the prevalence of each genotype in the population. Full mathematical and implementation details are provided in Methods and Additional file 1.

### Benchmarking over simulated data

**Fig. 2:**
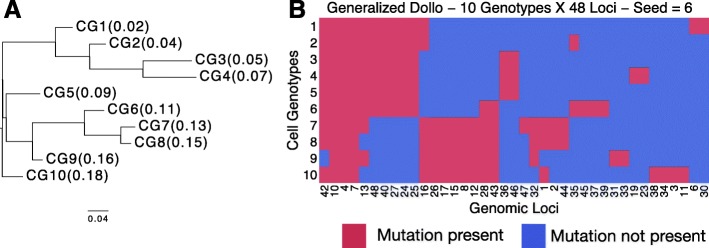
Simulated phylogenetic tree (panel **a**) and the resulting binarized cell genotype matrix (panel **b**). Transposed binarized simulated cell genotypes *Δ* from Generalized Dollo process over a fixed phylogeny. The original cell genotype matrix *Δ* ^CN^ is in copy number space. We binarize it by setting entries with non-zero variant allele copy number to one (*coloured red*) and setting entries with variant allele copy number of zero to zero (*coloured blue*). The clonal prevalence of each genotype is in parentheses

We benchmarked ddClone by simulating 10 ground truth synthetic datasets each with 10 cell genotypes and 48 genomic loci (Fig. 2). Joint bulk and single cell data were generated from a phylogenetic Dollo process (Additional file 1: Figure S1; Additional file 2).

We compared ddClone to three methods that operate on bulk data only: PyClone [2], PhyloWGS [22], and Clomial [9], and to two methods that leverage single cell data only: SCITE [20] and OncoNEM [18]. Two performance metrics were evaluated: clustering accuracy (by V-measure [23]) and accuracy of inferred cellular prevalences (the average over loci of the absolute differences between the inferred and true cellular prevalences). For the same bulk data, three sets of single cell data with different levels of noise were generated: (1) ideal data with no ADO or doublets; (2) data with moderate levels of sampling distortion, in the presence of 30% doublet cells and an ADO rate of 30%; and finally (3) data with higher levels of sampling distortion reflective of real data, with the same doublet and ADO rates as in (2). We designate these three regimes by *λ*=*∞*, *λ*=10, and *λ*=1.12 respectively. ddClone was supplied with the above single cell data for encoding the prior over clustering. Single cell-only methods were given the exact same input as ddClone’s prior.

**Fig. 3:**
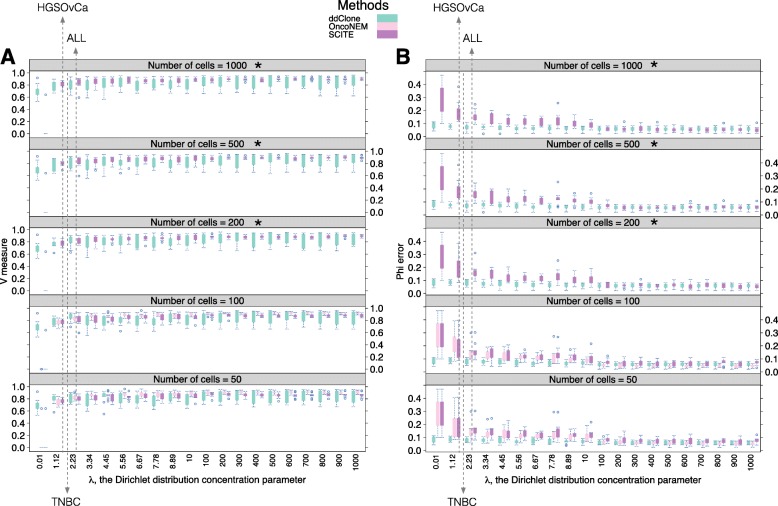
Performance analysis in presence of sampling distortion. Effect of sampling distortion on V-measure index (panel **a**) and mean absolute error of cellular prevalences (panel **b**) across multiple values for the total number of single cells (specified on *top of each panel*). Each box plot represents 10 simulated datasets each with 10 genotypes and 48 genomic loci. The cells are sampled from a Dirichlet-multinomial distribution with sample size *m*∈{50,100,200,500,1000} and parameters equal to the true prevalence of each genotype scaled by the concentration coefficient *λ*. The larger the *λ*, the closer the Dirichlet-multinomial distribution approximates the multinomial distribution. At higher values of *λ* the sampled cells better represent the true proportions of genotypes. Estimated values of *λ* for the real datasets are annotated on panel (**b**). We note that OncoNEM did not converge when number of cells exceeded 100 (boxes marked by a *star*). This result suggests that ddClone’s clustering and cellular prevalence estimates are fairly robust to the presence of distorted single cell sampling

Under noise levels corresponding to real datasets (*λ*=1.12, Fig. 3), ddClone_*λ*=1.12_ had a mean cellular prevalence estimation error of 0.09±0.03, significantly outperforming both OncoNEM_*λ*=1.12_ (0.17±0.03) and SCITE_*λ*=10_ (0.18±0.05), while doing slightly better than the second best performing bulk data-only method, PyClone (0.10±0.05). ddClone_*λ*=1.12_ also had high clustering accuracy in this noise regime, with a mean V-measure of 0.77±0.06 relative to 0.74±0.06 for OncoNEM_*λ*=1.12_, 0.71±0.08 for SCITE_*λ*=1.2_, and 0.71±0.10 for PyClone. Clomial had a slightly higher mean V-measure than PyClone (0.78±0.07), but it had a worse cellular prevalence estimation error (0.14±0.04). PhyloWGS had a mean V-measure of 0.73±0.03 and a mean cellular prevalence estimation error of 0.14±0.04.

Under *λ*=10, the moderate sampling distortion noise regime, ddClone_*λ*=10_ significantly outperformed both single cell data-only methods, in terms of cellular prevalence estimation, achieving a mean error of 0.07±0.02 versus OncoNEM_*λ*=10_’s 0.13±0.03 and SCITE_*λ*=10_’s 0.18±0.05. ddClone_*λ*=10_ did comparably well to OncoNEM_*λ*=10_ and SCITE_*λ*=10_ in terms of clustering accuracy, with a mean V-measure of 0.79±0.09 against 0.81±0.03 and 0.75±0.05 respectively.

With perfect, noiseless single cell data (*λ*=*∞*), OncoNEM_*λ*=*∞*_ outperformed SCITE_*λ*=*∞*_ and ddClone_*λ*=*∞*_ both in terms of cellular prevalence estimation, with an average error of 0.04±0.01 against 0.06±0.01 and 0.06±0.01, and in terms of clustering accuracy, with a mean V-measure of 0.90±0.03 versus 0.87±0.09 and 0.86±0.04 respectively.

**Fig. 4:**
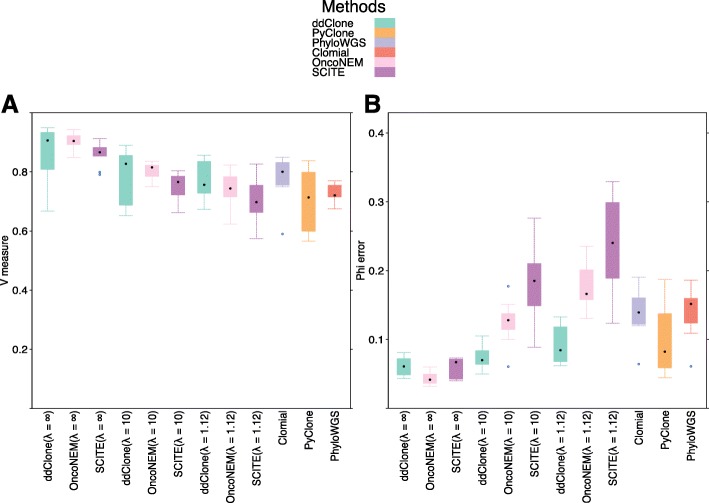
Benchmarking results over simulated data. Performance results for ddClone, single cell-only, and bulk data methods on ten synthetic datasets. ddClone and single cell-only methods were provided with single cells, either (1) 50 cells, sampled from a multinomial distribution with true genotype prevalences as parameters (labelled ddClone(*λ*=*∞*), OncoNEM(*λ*=*∞*), and SCITE(*λ*=*∞*)) in absence of doublet and ADO noise, or (2) 50 cells sampled from a Dirichlet-multinomial distribution with *λ*=10, constituting moderate to small levels of sampling bias (labelled as ddClone(*λ*=10), OncoNEM(*λ*=10), and SCITE(*λ*=10), or (3) 50 cells sampled from a Dirichlet-multinomial distribution with *λ*=1.12, constituting high levels of sampling bias (labelled as ddClone(*λ*=1.12), OncoNEM(*λ*=1.12), and SCITE(*λ*=1.12), where in the case of (2) and (3), 30% of cells are doublets and *r*_ADO_=30*%*. Panel **a** shows V-measure clustering performance. Panel **b** shows the average over loci of the absolute differences between the inferred and true cellular prevalences. This result shows that in the presence of reasonable levels of noise, ddClone performs comparably well in terms of both V-measure and the accuracy of inferred cellular prevalences

These results suggest that in the presence of simultaneous doublets, ADO events, and assortment bias noise, ddClone compares favourably well to other methods (Fig. 4). This is most relevant in the case of improved cellular prevalence estimates, as single cell platforms will likely stay unfit for this type of measurement in the near future due to under-sampling.

### Sensitivity to presence of noise in single cell data

We next directly considered the impact of four types of noise likely to be present in single cell data: ‘assortment bias’, where the quantity of sampled cells are not representative of the underlying tumour, ‘doublets’ and ‘allele drop-outs’ affecting the quality of the signal at a single genomic locus, and ‘genotype loss noise’, where one or more cell genotypes are unavailable (i.e. due to under-sampling) for formulation of the prior.

#### Assortment bias

Here we compare our method to methods that exclusively accept as input single cell sequencing data: OncoNEM [18] and SCITE [20]. In contrast to ddClone, these methods accept cell-mutation data and not a derived genotype-mutation matrix. In order to accommodate this in our experiments, we simulated cells from the genotypes as described below. See Additional file 1 for parameter settings and the derivation of cellular prevalence estimates for these methods. We note that even though ddClone is not designed to work with cell-mutation matrices, in the following simulations we have used this type of data to remove the effects of genotype inference methods (e.g. [16]) on the results. We investigated the effects of sampling bias modelled using the parameter *λ* (see Methods sections). For small values of *λ*, we expect the sampled cells not to be representative of the true tumour content and vice versa. With increasing assortment bias, ddClone performs better than single cell-only methods (Fig. 3), most importantly in *λ* ranges (Methods section) approximating the real datasets. When the sampled cells are accurate representations of the underlying sample, single cell-only methods outperform ddClone as expected, since prevalence estimates map directly to cell counting, without requiring inference.

#### Doublets

**Fig. 5:**
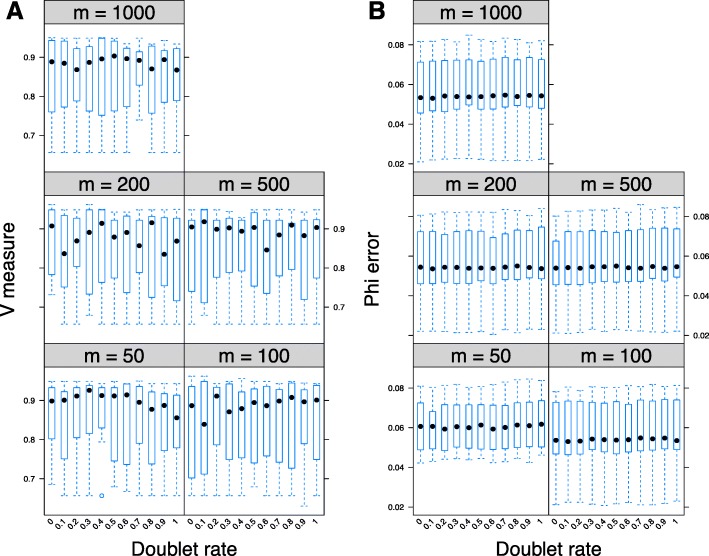
Performance analysis in presence of doublets. Effect of presence of doublets on V-measure index (panel **a**) and mean absolute error of cellular prevalences (panel **b**) across multiple values for the total number of single cells (specified as m on *top of each panel*). Each box plot represents 10 simulated datasets each with 10 genotypes and 48 genomic loci. The cells are sampled from a multinomial distribution with a sample size of m and parameters equal to the true prevalence of each genotype. Progressively increasing the percentage of doublet cells results in minor degrading performance in cellular prevalence estimate. Overall, this result suggests that ddClone’s cellular prevalence estimates are robust to the presence of uncorrected doublet noise

Doublets are one source of noise in single cell sequencing experiments. They occur when two or more cells are trapped together in a single well during the sequencing procedure. As the genotype assigned to a doublet well will be a hybrid of the genotypes of the two or more cells that it contains, we assume that this results in a false positive error where the hybrid genotype will have more mutated genomic loci than the original trapped cells (Methods). We simulated an additional 500 datasets across multiple values of *r*_doublet_, the percentage of doublet events, and multiple values of *m*, the number of sampled single cells, where *m*∈{50,100,200,500,1000} and *r*_doublet_∈(0,1]. ddClone’s cellular prevalence estimates are in general robust to the presence of uncorrected doublet noise (Fig. 5). We reiterate that ddClone is not designed to work with cell-mutation matrices, and the best input to it is the genotype-mutation matrix, for example, as generated by the SCG model. SCG is designed to correct for doublets, and we anticipate that using it would improve ddClone’s performance.

#### Allele drop-outs

**Fig. 6:**
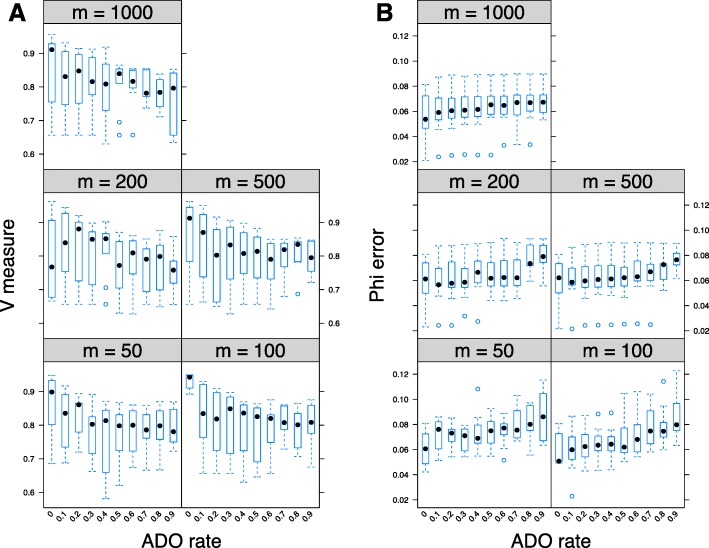
Performance analysis in presence of allele drop-outs. Effect of presence of allele drop-outs (*ADO*) on V-measure index (panel **a**) and mean absolute error of cellular prevalences (panel **b**) across multiple values for the total number of single cells (specified as m on *top of each panel*). Each box plot represents 10 simulated datasets each with 10 genotypes and 48 genomic loci. The cells are sampled from a multinomial distribution with a sample size of m and parameters equal to the true prevalence of each genotype. As expected, progressively increasing the ADO rate results in degrading performance in both clustering and cellular prevalence estimates. The detrimental effect dampens as the number of sampled cells increases

We next investigated the effect of increasing ADO (loci with ADO sit at the extremes of the allele count distribution; details in the Methods section) in ddClone accuracy. Progressively increasing the ADO rate results in degrading performance in both clustering and cellular prevalence estimates (Fig. 6). Unsurprisingly, the detrimental effect dampens as the number of sampled cells increases.

#### Clonal genotype loss

**Fig. 7:**
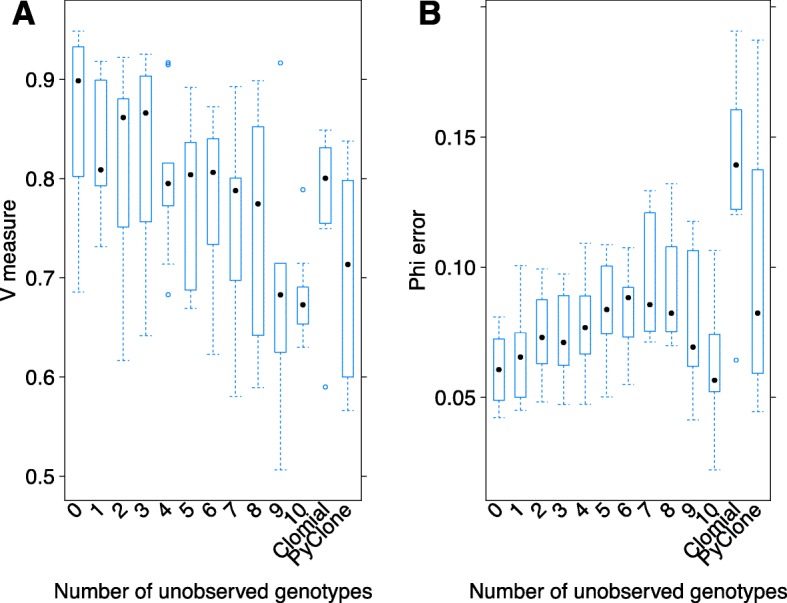
Performance analysis in presence of loss of multiple genotypes. Effect of removing genotypes on V-measure index (panel **a**) and mean absolute error of cellular prevalences (panel **b**). Unsurprisingly, progressively removing more cell genotypes (in increasing order of prevalence) results in monotonically degrading performance However, when as few as approximately half of the genotypes are available to encode in the prior, ddClone still outperforms the naive methods in terms of cellular prevalence estimate

Clonal genotype loss is defined as a lack of inclusion of a population’s genotype in the encoding of the prior. We under-sampled genotypes by systematically ‘hiding’ single cell genotypes from the prior. Unsurprisingly, progressively removing more cell genotypes (in increasing order of their prevalence) results in monotonically degrading performance (Fig. 7). However, when as few as approximately half of the genotypes are available to encode in the prior, ddClone still outperforms the naive methods in terms of cellular prevalence estimation (Figs. 4 and 7). This suggests a degree of robustness in the presence of under-sampling of clones, and that even partial prior information will improve prevalence estimates performance.

### Benchmarking over triple-negative breast cancer patient-derived xenograft data

**Fig. 8:**
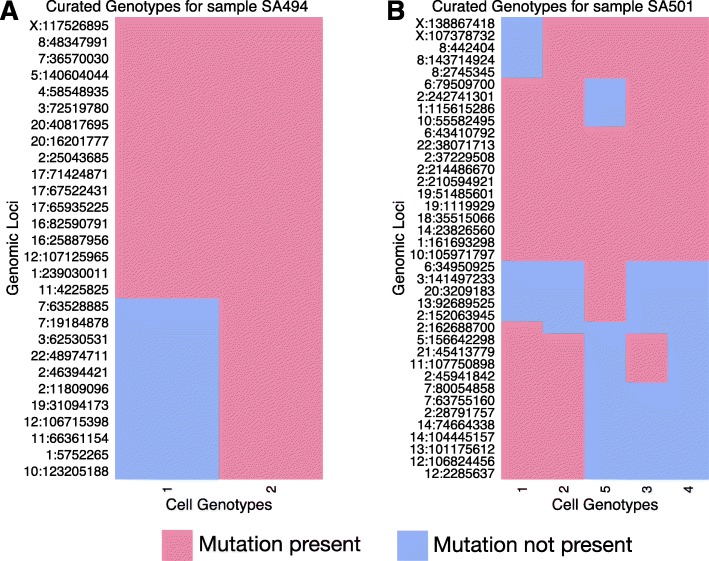
Genotypes curated for the triple-negative breast cancer data. Binary cell genotype matrices for sample SA494 over 28 genomic loci (*left*) and sample SA501 over 38 genomic loci (*right*). These are manually curated from a single cell genotype sequencing experiment [24]. Briefly, MrBayes was used to infer a consensus phylogenetic tree over the single nuclei. Then they were grouped into clades according to high probability branching splits. Finally, each clade was assigned a consensus genotype by taking the mode genotype of the clade at each genomic locus. Colour *red* indicates a mutated locus, while colour *blue* indicates a non-mutated locus

To test our method on a real dataset, we used a subset of samples from a triple-negative breast cancer (TNBC) xenograft study [24], where breast cancer tissues from 55 patients were transplanted into immuno-suppressed mice, resulting in 30 xenograft lines. Over 3 years, these lines were passaged up to 16 generations. Whole genome sequencing was performed over a subset of passages to identify point mutations at specific genomic positions. Deep targeted amplicon sequencing of between 100 to 300 SNV positions per sample was then used to establish the allelic prevalences of these mutations. We chose 210 cells from five timepoints that span two samples for single cell genotyping, and approximately 48 SNV positions were targeted for each timepoint, with some filtration due to poorly performing cells, or loci [24]. A consensus phylogenetic tree over cells was inferred using MrBayes [25]. Figure 8 shows the inferred cell genotype matrix *Δ* for each sample. In each timepoint, we only kept genomic loci that were shared between the bulk and single cell genotype data (Additional file 3).

**Fig. 9:**
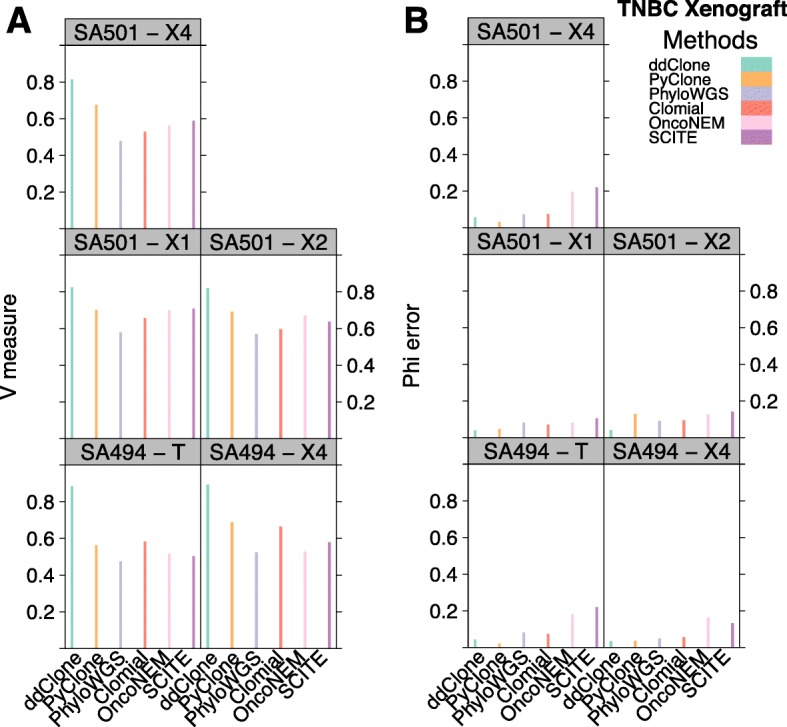
Benchmarking results over TNBC dataset. Performance results for ddClone and existing methods over TNBC SA501 X1, X2, X4, and SA494 T, X4. Panel **a** shows clustering assignment performance. Panel **b** shows cellular prevalence approximation mean absolute error. Evaluated against multi-sample PyClone, ddClone outperforms the second best performing method (PyClone) in terms of V-measure (Wilcoxon rank sum test with *p* value < 0.05) and performs as well (SA494, timepoint T) or better (all the other timepoints) than the second best performing method in terms of accuracy of inferred cellular prevalences

Since the exact clustering configuration and cellular prevalences of the genomic loci in the real dataset are unknown, we used the multi-sample PyClone results over several timepoints as a benchmark (see Additional file 1 for details). PyClone in multi-sample mode borrows statistical strength across all timepoints to give generally more accurate estimates of clonal structure in individual timepoints. We ran our method along with competing methods on each timepoint independently (Additional file 4). By these criteria, ddClone showed better performance than the second best performing method in terms of V-measure (Wilcoxon rank sum test with *p* value < 0.05) and performs comparably well (SA494, timepoint T and SA501, timepoint X4) or better (all the other timepoints) than the second best performing method in terms of accuracy of inferred cellular prevalences (Fig. 9).

ddClone achieved a V-measure of 0.88 and 0.89 for sample SA494 at timepoints T and X4 and 0.82, 0.82, and 0.81 for sample SA501 at timepoints X1, X2, and X4 respectively. The second best performing method, PyClone, achieved a V-measure of 0.56, 0.69, 0.70, 0.69, and 0.67 corresponding to sample SA494 at timepoints T and X4 and sample SA501 at timepoints X1, X2, and X4. Summarizing across samples, ddClone’s clustering was best (mean V-measure = 0.85, SD = 0.04), followed by PyClone (mean V-measure = 0.66, SD = 0.06), Clomial (mean V-measure = 0.61, SD = 0.06), SCITE (mean V-measure = 0.60, SD = 0.08), OncoNEM (mean V-measure = 0.60, SD = 0.08), and finally PhyloWGS (mean V-measure = 0.53, SD = 0.05). Use of the mean cellular prevalence estimation error resulted in a very similar ranking: ddClone (mean = 0.04, SD = 0.01), PyClone (mean = 0.05, SD = 0.04), Clomial (mean = 0.07, SD = 0.01), PhyloWGS (mean = 0.08, SD = 0.02), OncoNEM (mean = 0.15, SD = 0.05), and finally SCITE (mean = 0.16, SD = 0.05).

### Inference of genotypes from multiple spatial samples in ovarian cancer

We next evaluated performance on samples from a high-grade serous ovarian cancer (HGSOvCa) study [26] where 68 tumour samples from seven patients (5–13 samples per patient) including samples from the ovary and omentum were obtained during initial debulking surgery, except for one patient for whom samples from the first and second relapses were also available. Whole genome sequencing of 31 cryopreserved tissues and matched normal blood produced a panel of 3577 to 16,987 somatic genomic aberrations including SNVs and allele-specific absolute copy number variations (CNVs) per patient. To verify existence and allelic counts of these predicted SNVs, 37 formalin-fixed, paraffin-embedded specimens were used in targeted deep sequencing of 300 loci per patient with multiplex PCR amplicons. Single-nucleus sequencing of a total of 1680 cells from three patients was used to determine the co-occurrence of between 43 to 84 SNVs per sample. This data in combination with the single cell genotyper (SCG) model [16] produced the cell genotype matrix *Δ* for each sample. Similar to the xenograft TNBC case study, we only kept genomic loci that were shared between the bulk and single cell genotype data and evaluated the results analogously.

**Fig. 10:**
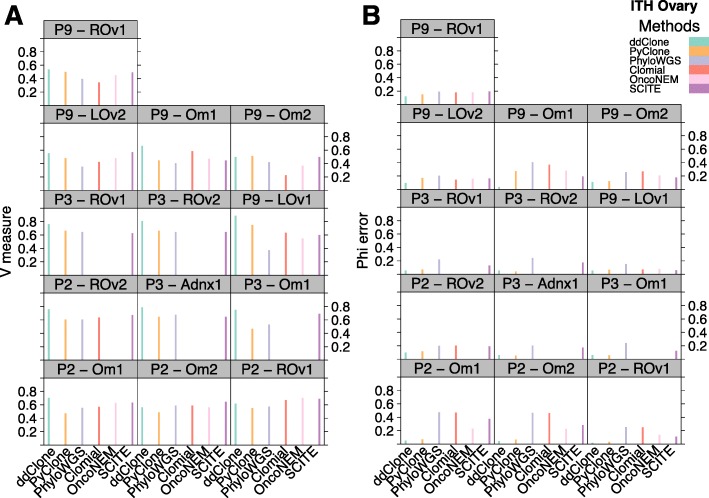
Benchmarking results over HGSOvCa dataset. Performance results for ddClone and existing methods over HGSOvCa data, from three patients: Patient 2 (P2) at sites Om1, Om2, ROv1, ROv2, Patient 3 (P3) at sites Adnx1, Om1, Rov1, Rov2, and Patient 9 (P9) at sites LOv1, LOv2, Om1, Om2, and ROv1. Panel **a** shows clustering assignment performance. Panel **b** shows cellular prevalence approximation mean absolute error. (*Om1*) Omentum sample 1, (*Om2*) Omentum sample 2, (*ROv1*) Right ovary sample 1, (*ROv2*) Right ovary sample 2, (*LOv1*) Left ovary sample 1, (*LOv2*) Left ovary sample 2, (*Adnx1*) Adnexa sample1

Measured against the multi-sample PyClone benchmark, ddClone outperforms all other methods in terms of clustering accuracy with a mean V-measure of 0.68 (SD = 0.12). The next best performing methods are SCITE (mean V-measure = 0.60, SD = 0.08), PyClone (mean V-measure = 0.56, SD = 0.10), OncoNEM (mean V-measure = 0.53, SD = 0.11), PhyloWGS (mean V-measure = 0.52, SD = 0.12), and finally Clomial (mean V-measure = 0.52, SD = 0.15). We note that although Clomial seems to tie with PhyloWGS, it did not converge over 4 out of 13 samples (P3 - Adnx1, P3 Om1, P3 - ROv1, and P3 ROv2). Similarly, OncoNEM did not converge over 5 out of 13 samples (P2 - ROv2, P3 - Adnx1, P3 - Om1, P3 - ROv1, and P3 - ROv2). This ranking is very similar in terms of the cellular prevalence metric where ddClone has the lowest cellular prevalence estimation error (mean = 0.07, SD = 0.03), followed by PyClone (mean = 0.10, SD = 0.07). OncoNEM ties SCITE with a mean cellular prevalence error equal to 0.19 (SD = 0.06 and SD = 0.08 respectively). Then comes PhyloWGS (mean = 0.27, SD = 0.11) and finally Clomial (mean = 0.27, SD = 0.14). These results suggest that using ddClone over single datasource-only methods may help avoid catastrophic estimation errors best exemplified in the Omentum site 1 in Patient 9 (P9 - Om1) where ddClone has a cellular prevalence estimation error less than one-fifth that of the second best performing method, SCITE (Fig. 10).

### Investigating mutation clusters in a patient with acute lymphoblastic leukemia

Here we analyse a dataset consisting in targeted sequencing of a panel of mutations (mostly SNVs) in 1479 single tumour cells from six patients with acute lymphoblastic leukemia (ALL) [12]. The genomic loci were assumed to be highly diploid. To confirm mutations in the single cell samples, the authors performed resequencing of the bulk samples over an average of 46 loci (between 10 to 105) for each patient.

**Fig. 11:**
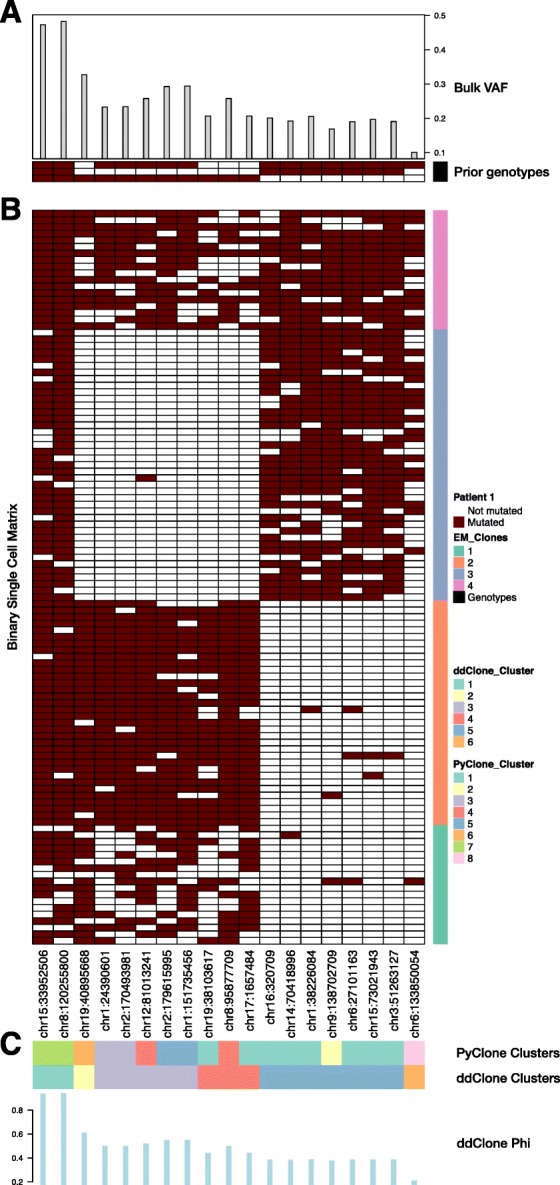
Analysis results of an acute lymphoblastic leukemia (*ALL*) dataset [12]. Analysis results of a patient with ALL (Patient 1) [12]. The variant allele frequencies (*VAFs*) from the bulk data (panel **a**, *top*) along with the consensus genotypes estimated from the binary cell matrix (panel A, bottom). These two constitute the input to the ddClone model. We note that the binary cell matrix **b** is displayed here for comparison and is not an input to ddClone. This binary cell matrix was used in [12] to cluster the cells into clones (*vertical bar* at the *right side of the figure*) and consensus genotypes (*bottom part* of panel **a**). ddClone clusters mutations into 6 groups (panel **c**, *top*) and estimates cellular prevalence (*Φ*) for each (panel **c**, *bottom*). ddClone’s estimated *Φ* are highly correlated with the corrected bulk VAFs (*R* ^2^=0.98, also see Additional file 1), suggesting that it does not introduce unreasonable structure in the data. Furthermore, when there is evidence in the bulk, it can override its prior and split clusters as necessary. For instance, even though locus chr19:40895668 has the same prior genotype as loci in cluster 4, its VAF in the bulk data is 1.5 times that of the mean of loci in cluster 4. This hints at a finer structure in cluster 4, and ddClone has automatically assigned chr19:40895668 to a separate cluster

Figure 11 shows ddClone’s analysis on one of the patients in this study (Patient 1). Four clones were reported in this dataset, one of which was labelled a doublet (Fig. 11, clone number 4) and was removed from subsequent analyses. The authors then extracted consensus genotypes for these clones (Fig. 11, panel A, bottom). ddClone finds six clusters. While single cell genotypes support a merger of clusters 4 and 2, ddClone splits them in two, placing locus chr19:40895668 in a separate cluster. This split is supported by the bulk data where the variant allele frequency (VAF) of chr19:40895668 is about 1.5 times that of the mean VAF of cluster 4 (0.33 and 0.22 respectively). Conversely, loci chr17:1657484 and chr1:38226084 have similar bulk VAFs (0.21 and 0.21 respectively), but since they have different prior genotypes, ddClone assigns them to separate clusters (clusters 4 and 5 respectively). PyClone assigns these two mutations to one cluster. We find similar instances in other patients in this dataset (see Additional file 1).

Due to the lack of multiple samples from within a patient, we were unable to use the same method we used to establish benchmark as in the other real datasets. Despite this, we confirm that ddClone’s estimated cellular prevalences are highly correlated with the reported bulk VAFs (*R* ^2^=0.85 across all patients), suggesting that ddClone does not introduce unreasonable structure in the results (Additional file 1).

### ddClone avoids co-clustering of mutations from distinct clones with shared cellular prevalences

Methods that cluster mutations based only on cellular prevalences are prone to grouping together mutations that belong to separate unique clones, if such clones happen to exist in similar cellular prevalences. Co-occurrence patterns from single cell data can be used to distinguish such clones. We define mutually exclusive mutations (MEMs) as a pair of mutations that never co-occur in clones inferred from single cell genotype analysis. The MEMs correspond to pair of mutations with a Jaccard distance of one (see Methods). PyClone, the second best performing method in terms of clustering, erroneously merges multiple MEMs in 8 out of 13 samples across three patients in the HGSOvCa data (Additional file 5). The numbers of pairs of MEMs erroneously merged by single-sample PyClone in each of the 8 samples are 13, 140, 259, 103, 169, 2, 14, and 1 respectively. Even multi-sample PyClone fails in correctly clustering MEMs in 9 out of 13 samples in the HGSOvCa data, although for markedly fewer mutations. The numbers of pairs of MEMs erroneously merged by multi-sample PyClone in each of the 9 samples are 5, 5, 5, 5, 2, 2, 2, 2, and 2 respectively. In contrast, ddClone only merged MEMs in 2 out of 13 samples (1 pair in the first sample and 2 pairs in the second sample) in the HGSOvCa data.

One pair of MEMs, 15:26990805 (SNV at chromosome 15, coordinate 26990805) and 5:38686543 (SNV at chromosome 5, coordinate 38686543) from Patient 3 in Omentum sample 1, had assigned cellular prevalences of 0.47 and 0.48 by PyClone, 0.43 and 0.46 by ddClone, and 0.41 and 0.41 by multi-sample PyClone respectively. PyClone and multi-sample PyClone both merged these MEMs; however, ddClone, while estimating a cellular prevalence in agreement with multi-sample PyClone (mean absolute difference of 0.03), separated them into different clusters. See Additional file 5 for a complete list of MEMs. In the TNBC xenograft data, PyClone erroneously merged 6 MEMs in 1 out of 5 samples. Neither multi-sample PyClone nor ddClone merged any MEMs. Another example is loci 17:1657484 and 1:38226084 in Patient 1 in the ALL dataset. They have similar bulk VAFs (both equal to 0.21) but different prior genotypes, and ddClone assigns them to separate clusters while PyClone co-clusters them. Taken together, results on real data suggest a marked advantage of using ddClone as measured by clustering accuracy. We note that the gains on prevalence error were more modest. We suggest this underscores the importance of single cell data to resolve mutation clustering as a reflection of genotype, while bulk data likely provides an accurate representation of mutation prevalence. Thus the ddClone approach can leverage the strengths of both measurement types and provide an overall improvement in the parameters of interest.

### ddClone overrides its prior in presence of evidence in the bulk data

ddClone is provided with a prior genotype-mutation matrix. When this prior encodes identical genotypes for two genomic loci, ddClone is very likely to cluster the pair together. However, if there is evidence in the bulk data suggesting that the mutations do not belong to a cluster, i.e. their bulk VAFs corrected for CNA are too dissimilar, we expect the model to *override its prior* and assign those genomic loci to separate clusters. We define prior overriding mutations (POMs) as a pair of mutations that have identical prior genotype, but are clustered separately by ddClone. The TNBC xenograft dataset had on average 41 (ranging from 32 to 61) POM pairs. For instance, in sample SA501, timepoint X1, 20:3209183 and 2:152063945 were a POM pair with a corrected bulk VAF of 6. On average about 10 (from 0 to 27) POM pairs were in the HGSOvCa data, including genomic loci 9:35546540 and X:154158018 from Patient 2, Omentum site 2 with a corrected bulk VAF of 1.56. In the ALL dataset, in Patient 1, loci chr19:40895668 and chr17:1657484 had identical prior genotypes, but a corrected bulk VAF ratio of 1.4, and ddClone put them into separate clusters. In this dataset, Patients 1 to 5 had 3, 4, 105, 320, and 1264 such pairs, with an average corrected bulk VAF ratio of 1.36±0.13, 1.61±0.25, 1.72±0.61, 1.40±0.39, and 1.69±1.19 respectively. There were no such pairs in Patient 6.

## Conclusions

The ddClone approach presented here exemplifies the combined statistical strength of orthogonally derived observations for inference of clonal populations from NGS sequencing. Single cell sequencing methods are continually improving; however, they will likely always be limited by the effect of small DNA inputs and sparsely sampled cell populations. Bulk methods, on the other hand, will require computational deconvolution approaches to disentangle the unobserved underlying clonal constituents used to generate a measurement of interest. Here we show that bulk and single cell measurements when fused together with joint statistical inference can overcome the limitations of both methods, leading to more accurate inference. Single cell sequencing experiments typically generate a bulk template as a control sample, and so statistical integration can be ubiquitously applied. In particular, we show how ddClone resolves clonally mutually exclusive mutations which would otherwise be co-clustered in bulk, therefore underestimating the number of clones present in a sample of interest. We note that samples analysed by ddClone from the ovarian cancer study were heavily intermixed, as reported in [26], representing a situation where multiple clones co-existed in different anatomic sites at relatively equal prevalence. This is similar to what might be observed in haematological malignancies where relatively less anatomic isolation of clones is the default model for clonality and thus clones are likely to co-exist at equal prevalence [12]. Failure to resolve clones in these scenarios could lead to poor and spurious biological interpretation and underestimation of tumour complexity. Multiple samples where clonal prevalences vary would lead to more accurate inference as demonstrated by [2]; however, we show in the single sample scenario that ddClone can overcome under-clustering of mutations that arises from multiple clones co-occurring at near equal prevalences.

While the ddClone presents an advance in statistical integration, several limitations remain. As investigators continue to dissect longitudinal clonal dynamics through temporal sampling, extensions to leverage statistical signals across multiple samples will be necessary. Furthermore, we expect the method will generalize well to different single cell platforms offering longer reads with phased mutations. However, considering more mutations will come at a computational cost that may not scale to whole genome dimensions. This may limit the utility of ddClone in the case of whole genome analysis. In addition, we showed with theoretical and simulated ‘clean’ single cell data that single cell-only methods outperform ddClone. This is expected and reflects, in the context of future potential for accurate single cell methods, the need for bulk observations to infer prevalence of clones may diminish.

Analogously, there are some scenarios in which bulk data may be a biased representation of the underlying tumour, for instance, due to sampling from spatially separated regions of the tumour [26]. This may suggest that investigators should take caution in matching samples from single cell and bulk data.

We emphasize that multi-sample PyClone does not constitute ground truth. For example, we observe some erroneous clustering of mutations based on VAFs in its results. Nevertheless, previous research demonstrates that using samples from multiple regions or timepoints improves the accuracy of the clonal structure inference methods [8, 9, 27] since statistical strength can be borrowed across multiple measurements. In this context, we use multi-sample analysis as a convenient benchmark against which we quantitatively assess performance using single sample data. This may be suboptimal, and thus our study illuminates the need to create ground truth datasets either through extensive orthogonal measurement or through engineered admixtures of related cell populations in defined proportions.

We focused our work on point mutations in this report, but other clonal marks such as structural variations and epigenetic markers can be used to infer clonal composition and dynamics. Extensions to the model for features with different statistical properties will be required to integrate non-point-mutation features of the genome. The use of Jaccard index to summarize the prior genotypes in our model may be suboptimal, due to different noise levels, among other reasons. We implemented an augmented Jaccard index taking this asymmetry into account. While for the majority of datasets it has marginal effect, it improves the performance of ddClone in one of the real datasets analysed here. Continued improvement of summary statistics, including for example phylogenetic models, to encode prior knowledge should lead to further increases in accuracy.

Finally, the model we have proposed is unidirectional, encoding single cell data as a Bayesian prior and bulk data with a likelihood model. Future improvements may be realized by implementing a bi-directional inference framework which iteratively improves predictions from bulk data informed by single cell and single cell data informed from bulk data. These limitations represent open problems for future work stimulated by our contribution here. We anticipate that our work here lays a foundation upon which complementary bulk and single cell measurements in cancer can be statistically integrated to sharpen the investigator’s view of clonal dynamics. We contend this is an important step towards ultimately realizing quantitative fitness properties leading to a deeper understanding of cancer progression and morbidity in patients.

## Methods

### Concepts and definitions

**Fig. 12:**
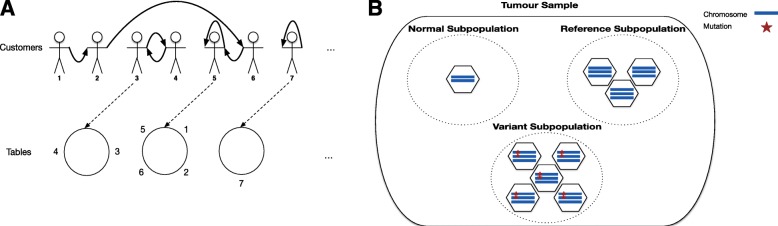
Hypothesized sitting arrangement in ddCRP/subpopulation assumptions in the bulk data. **a** Induced table sitting *T*(*C*) by a particular customer connection configuration *C*. *Bold arrows* show customer connections and *dotted arrows* point to equivalent table sittings. Since customer 7 only has a self-loop, the corresponding table has only one customer. **b** Our assumption about clonal architecture in the tumour with respect to a particular genomic locus. In this example, normal subpopulation represents a collection of un-mutated diploid cells. Reference subpopulation comprises cells that have a copy number amplification event, but no single nucleotide mutations. Variant subpopulation is a collection of cells that have an SNV at the particular genomic locus

Given (1) variant allele counts and (2) copy number at each genomic locus, (3) tumour cellularity, and (4) single cell genotype data, our method infers (a) cellular prevalences and (b) cluster assignments for those genomic loci. We review these notations below. Variant allele counts. We assume that at each genomic locus *i*, a total of *d*_*i*_ reads map to locus *i*, out of which *b*_*i*_ reads harbour the variant allele. Variant allelic prevalence. The expected fraction of reads, *ξ*, that harbour the variant allele. However, this quantity is not observed directly; rather, we observe, for each locus of interest, the number of variant reads divided by the total number of reads in all cells. Copy number at each genomic locus. Copy number variations influence the allelic prevalence *ξ*. An example of this influence is shown in Fig. 12 b, where $\xi = \frac {2 \times 5 }{2 \times 1 + 3 \times 3 + 3 \times 5} = \frac {5}{13}$. Tumour cellularity, *t*. The fraction of cancer cells in the sample. Hence the fraction of normal cells would be 1−*t*. We assume that tumour cellularity is estimated independently from our model. Cell genotype data. Let *M* denote the number of cell genotypes in the tumour sample and *N* be the number of genomic loci in our model. Cell genotype data is modelled as a binary matrix *Δ*∈{0,1}^*M*×*N*^ with rows corresponding to cell genotypes and columns to genomic loci. *Δ*_*m*,*n*_=1 if the genotype *m* is mutated at locus *n*. We assume in this work that cell genotype data are derived from single cell sequencing studies.

The desired outputs are cluster assignments of genomic loci and their cellular prevalences. Cellular prevalence *ϕ*_*i*_ for a particular genomic locus *i* is defined as the fraction of cells in the sample that harbour a mutation at that genomic locus. For example, in Fig. 12b cellular prevalence for the depicted genomic locus is $\frac {5}{9}$. Thus 1−*ϕ*_*i*_, the fraction of cancer cells from the reference population, is $1- \frac {5}{9} = \frac {3}{9}$. We define the clonal prevalence of a genotype to be the fraction of cells in the tumour sample harbouring that genotype.

### Notation

Let *X*={*x*_1_,*x*_2_,…,*x*_*N*_} be the set of the *N* genomic loci of interest, indexed by *ϖ*={1,2,…,*N*}.

We adopt the notation *j*:*i* for $j \le i, j,i \in \mathbb {N}$ to denote {*j*,*j*+1,*j*+2,…,*i*}, a subset of successive integers.

We define a clustering of *X* as a partition *T* of its index set *ϖ*, that is, *T*={*T*_1_,*T*_2_,…,*T*_*K*_} such that ⊔_*k*∈1:*K*_ *T*_*k*_=*ϖ* where *K* is the number of partitions, ⊔ denotes the disjoint union operator, and each subset *T*_*k*_ is called a cluster.

We define *x*_*A*_ for *A*⊂*ϖ* to be {*x*_*i*_|*i*∈*A*}. For example, $x_{T_{k}}$ is the set of data points in cluster *T*_*k*_ and *x*_*i*:*j*_={*x*_*i*_,*x*_*i*+1_,*x*_*i*+2_,…,*x*_*j*_}.

Furthermore, let $T(.) \colon \mathbb {N} \to \mathbb {N}$ map data point indices to their clusters, that is, *T*(*i*)=*k* iff *i*∈*T*_*k*_.

#### Partitions of a graph

Let $\mathbb {G}(\mathcal {V}, \mathbb {E}) $ denote an undirected graph $\mathbb {G}$ where $\mathcal {V}$ is the set of vertices and $\mathbb {E}$ is the set edges, i.e. a set of unordered pairs $\{u, v\} \subset \mathcal {V}$. The set of edges $\mathbb {E}$ induces a partitioning on $\mathcal {V}$, where each connected component of $\mathcal {V}$ corresponds to a cluster. With a slight abuse of notation, let $T(\mathbb {E}) = T(\mathbb {G}(\mathcal {V}, \mathbb {E}))$ denote this partitioning and $T^{k}_{\mathbb {E}}$ denote its *k*-th cluster.

A directed graph $\mathcal {G}(\mathcal {V}, \mathcal {E})$ consists in a set of vertices $\mathcal {V}$ and a set of directed edges $\mathcal {E}$ where each edge is an ordered pair of vertices. For a directed graph $\mathcal {G}$, we define its underlying undirected graph $U(\mathcal {G})$ to be the graph obtained by replacing all directed edges in $\mathcal {G}$ with undirected ones. Let $T(\mathcal {E})$ be the partitioning induced by $U(\mathcal {G})$, the underlying undirected graph of $\mathcal {G}$. Throughout this document the $\mathcal {G}$ corresponding to $\mathcal {E}$ is always apparent from the context, with $\mathcal {V}$ always being the set of our data points. Let $T_{\mathcal {E}}\colon \mathbb {N} \to \mathbb {N}$ map vertex indices to their clusters, that is, $T_{\mathcal {E}}(i) = k $ iff $i \in T^{k}_{\mathcal {E}}$.

### Traditional CRP

ddCRP can be explained through an alternative representation of the Chinese restaurant process (CRP). We follow the notation in [21]. In the traditional CRP, customers enter a Chinese restaurant and opt to sit at a table where the probability of joining a table is proportional to the number of customers already sitting at that table. Customers may also choose to sit at a new table with probability proportional to *α*, a model parameter. In the Chinese restaurant metaphor, customers represent the genomic loci and tables represent clusters [28].

Let *z*_*i*_ denote the table assignment for customer *i* and assume that customers 1:*i*−1 have occupied tables 1:*K*, and let *n*_*k*_ be the number of customers sitting at table *k*. The customer sitting configuration induces a partitioning of customer indices. The CRP draws *z*_*i*_ as in Eq. (1).

1 $$  p\left(z_{i} = k | z_{1:(i-1)}, \alpha\right) \propto \left\{ \begin{array}{ll} n_{k} & \text{for}~k \le K \\ \alpha & \text{for}~k = K + 1 \end{array} \right.  $$

### Alternative representation of traditional CRP

Traditional CRP can equivalently be viewed as customers joining other customers instead of joining other tables. Let *c*_*i*_ denote the customer index with whom customer *i* is sitting and *C*=*c*_1:*N*_.

This defines a directed graph $\mathcal {G}(\mathcal {V}, \mathcal {E})$ with $\mathcal {V}$ the set of customer indices and $\mathcal {E}$ the set of ordered pairs (*i*,*c*_*i*_).

As described above, this induces $T_{\mathcal {E}} = T(C)$, a partitioning of customer indices. Each cluster corresponds to a table in the traditional representation. Figure 12 a shows an example *C* and its corresponding *T*(*C*).

In a generalization of this model, the probability for a customer *i* to connect to a customer *j* is proportional to a function of the distance between them. The distance matrix *D* encodes our knowledge about the data points’ dissimilarity from a secondary source. In this work, this distance matrix is computed from the cell genotypes derived from single cell genotyping experiments. The non-increasing decay function *f* takes non-negative finite values. This is summarized in Eq. 2.

2 $$  p(c_{i} = j | D, \alpha) \propto \left\{ \begin{array}{ll} f\left(d_{i,j}\right) & \text{for}~i \neq j \\ \alpha & \text{for}~i = j \end{array} \right.  $$

This defines the ddCRP model. We note that picking a constant decay function *f*(*x*)=1 reduces ddCRP to traditional CRP, since in that case, Eq. (2) is identical to Eq. (1).

### The ddClone model

We assign each genomic locus to a customer. Throughout this document, we use cell genotype data from single cell genotyping studies to compute the distance between genomic loci. We note that this is not a requirement of the model, and other sources could be used to define dissimilarity between genomic loci.

#### Distance matrix

We have used the Jaccard distance to form the distance matrix *D*∈ [ 0,1]^*N*×*N*^ between genomic loci. The Jaccard distance is computed as 1−JaccardIndex that is:

3 $$ \text{JaccardDist}(A, B) = 1 - \frac{|A \cap B|}{|A \cup B|} = 1 - \frac{\sum_{i=1}^{M} (A_{i} \times B_{i})}{\sum_{i=1}^{M} (A_{i} + B_{i})}  $$

where *A*_*M*×1_ and *B*_*M*×1_ are binary column vectors, each representing a genomic locus. Intuitively, this assigns a higher distance to genomic loci that co-occur less often in the single cell genotypes and vice versa. We note that our use of the Jaccard index to compute distances between genomic loci is related to distance-based phylogenetic inference methods [29].

As the Jaccard index is agnostic to the different FN and FP noise rates inherent in the single cell data, we have proposed and investigated an augmented (modified) Jaccard distance (MJD). The results show that while over simulated data, MJD has a marginal effect on ddClone’s performance, using MJD substantially improves performance over one of the real datasets. See Additional file 1 for the formulation and more details.

Let *λ*={*s*,*α*,*a*} be the collection of hyperparameters in our model. For brevity, we first assume that these hyperparameters are fixed, and in Additional file 1 discuss their resampling scheme.

#### Bulk population assumptions

Similar to PyClone, we make the simplifying assumption that the clonal population in the bulk data, with respect to a specific mutation, comprises three subpopulations: the normal, the reference, and the variant subpopulations. Figure 12b illustrates this assumption. To avoid confusion with the cell genotype states coming from the single cell sequencing study, we refer to the assumed copy number of the subpopulations in the bulk data as locus genotypes. This data is usually not available directly from the bulk data and has to be inferred or accounted for in the inference procedure.

#### Locus genotype state priors

Let $\psi _{i} = \left (g^{i}_{N}, g^{i}_{R}, g^{i}_{V}\right) \in (\mathbb {N}_{0} \times \mathbb {N}_{0})^{3}$ represent the assumed locus genotype state at each genomic locus *i* in the bulk data where $\mathbb {N}_{0} = \mathbb {N} \cup \{0\}$.

Let $g^{i}_{N}$ represent the normal locus genotype *N*, $g^{i}_{R}$ represent the reference locus genotype *R*, and $g^{i}_{V}$ represent the variant locus genotype *V*. Each $g^{i}_{S}$ is a pair of non-negative integers that denote the copy number for the locus genotype *S*∈{*N*,*R*,*V*} at the genomic locus *i*. For example, $g^{i}_{N} = (2,3)$ means that the normal locus genotype in the bulk tumour sample has two copies of the reference allele and three copies of the variant allele at genomic locus *i*. Here (0,0) denotes a homozygous deletion. For $g \in \mathcal {G} = \mathbb {N}_{0} \times \mathbb {N}_{0}$, let $\zeta \colon \mathcal {G} \to \mathbb {N}_{0}$ be the total copy number of locus genotype *g*. We define *μ*(*g*), the probability of sampling a variant allele from a subpopulation with locus genotype *g*, as follows:

$$ \mu(g) = \left\{\begin{array}{ll} \epsilon & \text{for} b(g) = 0 \\ 1 - \epsilon & \text{for} b(g) = \zeta(g) \\ \frac{b(g)}{\zeta(g)} & \text{otherwise} \\ \end{array} \right. $$

where *ε* is the sequencing error probability, the probability of observing a variant allele when sequencing a true reference allele.

To capture the effects of locus genotypes, cellular prevalence, and tumour cellularity, we define *ξ*(*ψ*,*ϕ*,*t*) as follows:

$$\begin{aligned} {}\xi (\psi, \phi, t) = \frac{(1-t)\zeta(g_{N})}{Z} \mu(g_{N}) + \frac{t(1-\phi)\zeta(g_{R})}{Z} \mu (g_{R}) + \\ \frac{t \phi \zeta(g_{V})}{Z} \mu (g_{V}) \end{aligned} $$

where *Z*=(1−*t*)*ζ*(*g*_*N*_)+*t*(1−*ϕ*)*ζ*(*g*_*R*_)+*t* *ϕ* *ζ*(*g*_*V*_) is the normalizing constant.

To compute the likelihood, we sum over possible values of *ψ*_*i*_. Since the discrete space of *Ψ* values quickly becomes intractable, we only consider a limited number of locus genotypes. This is done by defining an informative prior *π*_*i*_ over *ψ*_*i*_ (more details are given in Additional file 1).

#### The likelihood function

Given the priors over locus genotypes, the emission likelihood for one locus is:

4 $$  {} p\left(b_{i} | \phi_{i}, d_{i}, \pi_{i}, t\right) = \sum_{\psi_{i} \in \mathcal{G}^{3}} p\left(b_{i} | \phi_{i}, d_{i}, \psi_{i}, t\right) p\left(\psi_{i} | \pi_{i}\right)  $$

To address overdispersion, we have modelled the conditional distribution of variant allele counts *b*_*i*_ with a beta-binomial distribution, characterized in terms of mean and precision as follows:

5 $$  {}p(b | d,m,s) = {d \choose b} \frac{B(b+sm, d-b + s(1-m))}{B(sm, s(1-m))}  $$

where *B* is the beta function. To reflect our assumptions over the sample sub-population structure, we set the mean value to a function of locus genotypes, cellular prevalence, and cellularity for each data point, that is, *m*=*ξ*(*ψ* ^*n*^,*ϕ* ^*n*^,*t*). To reduce the number of parameters, all loci share the same precision *s*.

### Synthetic data simulation

#### Single cell instantiation

To simulate cells, we first sample observed prevalences $\Phi = \{\Phi _{1}^{\text {observed}}, \Phi _{2}^{\text {observed}}, \ldots, \Phi _{M}^{\text {observed}}\}$ for each genotype from a Dirichlet distribution *Φ*_observed_∼Dir(*λ* *Φ*), where *Φ*={*Φ*_1_,*Φ*_2_,…,*Φ*_*M*_} are the true prevalences for genotypes 1 to M. We then simulate *m* cells from a multinomial distribution with parameters *Φ*_observed_, i.e. (*n*_1_,*n*_2_,…,*n*_*M*_)∼Mult(*Φ*_observed_) where *n*_*i*_ is the number of cells that have genotype *i*. This process is equivalent to sampling the cells from a Dirichlet-multinomial distribution, that is, (*n*_1_,*n*_2_,…,*n*_*M*_)∼Dirichlet-multinomial(*λ* *Φ*). The larger the *λ* is, the closer are the two vectors *Φ*_observed_ and *Φ*. In fact, as the value of *λ* grows, the Dirichlet-multinomial distribution progressively better approximates the multinomial distribution. For each dataset, we represent the average error between true and observed prevalences by $e_{\Phi } = \frac {1}{M} \sum _{1}^{M} |\Phi _{i} - \Phi _{i}^{\text {observed}}| $, the average absolute difference between true and observed genotype prevalences. We measure the discrepancy between the true and the observed prevalences by the number of absent genotypes in the samples of cells and by *e*_*Φ*_, the average error between true and observed prevalences.

For *λ*=0.01, on average only about 1 out of 10 genotypes is observed in the sampled cells and *e*_*Φ*_=0.17. In contrast, when *λ*=1000, on average, more than 9 out of 10 genotypes are observed and observed prevalences closely resemble the true genotype prevalences (*e*_*Φ*_=0.008).

#### Modelling doublet noise

Assume *K* cells *c*_1_,*c*_2_,…,*c*_*K*_ with genotypes $\Delta _{c_{1}}, \Delta _{c_{2}}, \ldots, \Delta _{c_{K}}$ are trapped in a well *w*_*d*_, where $\Delta _{c_{i}}$ correspond to rows in the binary genotype matrix *Δ* as defined in the Methods section. We define the reported genotype for *w*_*d*_ as the logical OR between genotypes of its constituent cells, i.e. $ \Delta _{W_{d}} = \Delta _{c_{1}} \text {OR} \Delta _{c_{2}} \text {OR} \ldots \text {OR} \Delta _{c_{K}}$. In this study we assume that, for a doublet, exactly two cells are trapped in a well simultaneously (*K*=2).

For a fixed value of *r*_doublet_, we first sample *m* cells as the original set. Second we sample an extra *r*_doublet_∗*m* cells to act as co-trapped cells. Finally, we randomly pick *r*_doublet_∗*m* of the original set and combine each with one of the cells from the co-trapped cells by recording the logical OR of their respective genotypes. These constitute the doublets. Algorithm 1 shows the pseudo code for simulating doublets.



In Algorithm 1, sampleCells(*Δ*,*m*) is a method that, given a genotype matrix *Δ*, returns an array *X* of size *m*, where the *i*-th item *X*[*i*] is a row in the genotype matrix *Δ*.

#### Modelling allele drop-out noise

To simulate the effect of ADOs, we first pick m cells from a multinomial distribution with parameters equal to the true prevalence of each genotype, that is, (*n*_1_,*n*_2_,…,*n*_*M*_)∼Mult(*Φ*), where *n*_*i*_ is the number of cells that have genotype *i*, $\sum _{i = 1}^{M} = m$, and *Φ* is the true prevalence of each genotype. This results in a binary cell-genotype matrix *G*∈{0,1}^*m*,*M*^ with rows corresponding to sampled cells and columns corresponding to genomic loci where *G*_*i*,*j*_=1 if cell *i* is mutated at locus *j*. We assume that ADO affects a cell by turning a mutated locus into an unmutated one and causing a false negative error. When an unmutated locus is affected, it mimics a deletion and does not alter the genotype matrix. At a fixed ADO rate, *r*_ADO_, we randomly pick *r*_ADO_ of the mutated loci across all sampled cells and set their value to zero. This constitutes the modified binary matrix *G* that we use as input to ddClone. Algorithm 2 shows the pseudo code for simulating allele drop-out noise.



### Inference

We use a Gibbs sampler to draw samples from the posterior distribution of the model. We initialize the sampler such that all customers are in their own clusters. Let *c*_−*i*_ be the customer connection configuration with customer *i*’s outgoing connection removed. Let *x*_*i*_=(*b*_*i*_,*d*_*i*_) denote the observed data, namely, variant and total allele counts.

The full conditional distribution of *c*_*i*_ is:

6 $$ p\left(c_{i} | c_{-i}, x_{1:N}, \lambda\right) \propto p\left(c_{i} | \lambda\right)p\left(x_{1:N} | c_{i}, c_{-i}, \lambda\right)  $$

where *p*(*c*_*i*_|*λ*) is the same as Eq. (2) and *λ* is the set of all hyperparameters. Let $x_{T_{k}}$ be the set of customers in cluster *T*_*k*_ or, equivalently, the set of customers sitting at table *k*, then the likelihood term factors in:

7 $$ p(x_{1:N} | c_{-i}, c_{i} = j, \lambda) = \prod_{T_{k} \in T(C)} p\left(x_{T_{k}} | \lambda\right)  $$

where *T*(*C*) is the partitioning induced by current customer connection configuration *C*. The term $p(x_{T_{k}} | \lambda)$ further expands as:

8 $$ p\left(x_{T_{k}} | \lambda\right) = \int \left(\prod_{i \in T_{k}} p\left(x_{i} | \theta, \lambda\right)\right)p(\theta | \lambda)d\theta  $$

where the likelihood *p*(*x*_*i*_|*θ*,*λ*)=*p*(*b*_*i*_|*ϕ*_*i*_,*d*_*i*_,*π*_*i*_,*t*) is the same as Eq. (4).

Since our prior over cellular prevalences *ϕ*_*i*_ is non-conjugate to the likelihood, we resort to a cached version of Griddy Gibbs method [30] to compute the above integral. At the end of each iteration (i.e. when all customers are reassigned), we sample *ϕ*_*k*_ for each cluster *k* as follows:

9 $$ {}\phi_{k} \sim p\left(\phi_{k} |x_{T_{k}}, \pi_{T_{k}}, t, \lambda\right) \propto p\left(\phi_{T_{k}} | \lambda\right) p\left(x_{T_{k}} | \phi_{T_{k}}, \lambda, \pi_{T_{k}}, t\right)  $$

where $p(\phi _{T_{k}} | \lambda)$ is the probability density function of a uniform distribution.

### Approximating *λ* in real datasets

First, we computed, for simulated datasets with various values of *λ*, the concordance between bulk and single cell data as measured by the coefficient of determination (*R* ^2^), that is, how well mutation cellular prevalences (*ϕ*) estimated from the bulk data correspond to that estimated from the single cell data.

We then measured the observed concordance between mutation cellular prevalences as estimated from bulk data by multi-sample PyClone (for TNBC xenograft and HGSOvCa datasets) or corrected bulk VAFs (for the ALL dataset) and single cell data. Lastly, we compared at what value for *λ*, the *R* ^2^ value in the simulated dataset matched the *R* ^2^ value of each real dataset. The estimated *λ* values are 1.13±0.31, 2.00±0.21, and 2.24±0.21 for the HGSOvCa, TNBC, and ALL datasets respectively. For the ALL dataset, in computing the coefficient of determination, we set aside the outlier Patient 5 which had an *R* ^2^=0.08. We note that since single cell data in the real dataset are affected by sources of noise other than sampling distortion, including doublets and ADOs, the above procedure overestimates *λ*.

### Clustering summarization

To cluster genomic loci we first compute the posterior similarity matrix and then maximize the PEAR index to compute a point estimate [31] as implemented in the R package mcclust [32]. We estimate the cellular prevalence for each genomic locus as the mean of after burn-in Markov chain Monte Carlo (MCMC) samples.

### Computational complexity

Computing the distance matrix takes $\mathcal {O} (N^{2}M)$ where *N* and *M* are the rows and columns of the input matrix to ddClone. In the intended use of ddClone, the input matrix would be the binary genotype matrix *Δ*, in which case *N* is the number of genotypes and *M* is the number of genomic loci. Computing the clustering result takes $\mathcal {O} (M^{2})$. The complete analysis with 10,000 MCMC iterations on a machine with 40x cores of Intel Xeon 2.20GHz CPU and 500GB of RAM, for a dataset of 37 genomic loci, takes about 6 hours (365.9±47.32 minutes) to finish (averaged on 4 samples from Patient 2 in the HGSOvCa dataset).

## Additional files

Additional file 1 Supplementary information. A PDF file that contains supplementary figures, details of the mathematical derivation of the ddClone model, and the description and algorithms to simulate data from the Generalized Dollo model. (PDF 2200 kb)

Additional file 2 Simulated data inputs. An excel file containing bulk allele counts and single cell genotypes generated from the Generalized Dollo model used as input to ddClone and existing methods in simulation studies. These data were used to assess the performance of all methods considered in this study and resulted in Fig. 3. (XLSX 84 kb)

Additional file 3 Real data inputs. An excel file containing bulk data allele counts and single cell genotypes from both TNBC xenograft and ITH HGSOvCa datasets used as input to ddClone and existing methods. These data were used to assess the performance of all methods considered in this study and resulted in Figs. 9 and 10. (XLSX 67 kb)

Additional file 4 Real data benchmarks. An excel file containing the multi-sample PyClone results for both TNBC xenograft and ITH HGSOvCa datasets used as benchmark to assess the performance of ddClone and existing methods. The performance metrics reported in Figs. 9 and 10 are measured against these results. (XLSX 32 kb)

Additional file 5 List of mutually exclusive mutations (MEMs). A zip archive of five txt files that list the MEMs (see the main text) found in TNBC xenograft and ITH HGSOvCa datasets as well as the MEMs merged by PyClone and multi-sample PyClone. (ZIP 7 kb)
