# Supplementary material for: ddClone: joint statistical inference of clonal populations from single cell and bulk tumour sequencing data
Source: Genome Biol. 2017 Mar 1;18:44. doi: 10.1186/s13059-017-1169-3 (PMC5333399; doi:10.1186/s13059-017-1169-3)
Supplement: Additional file 1 — Supplementary information. A PDF file that contains supplementary figures, details of the mathematical derivation of the ddClone model, and the description and algorithms to simulate data from the Generalized Dollo model. (PDF 2200 kb) [file 13059_2017_1169_MOESM1_ESM.pdf]

Joint statistical inference of clonal populations from single cell and bulk tumour sequencing data

Supplementary Materials

Sohrab Salehi, Adi Steif, Andrew Roth, Samuel Aparicio, Alexandre Bouchard-Côté, and Sohrab P. Shah

December 25, 2016

Contents

1 Supplementary figures **4**

2 The ddClone model **52**

2.1 Simplified generative model 53

2.2 Generative process for ddCRP mixture modelling 54

2.3 The Parental Copy Number (PCN) prior 55

2.4 Resampling hyperparameters 57

2.5 The modified Jaccard distance 58

3 Simulation **59**

3.1 Simulating genotypes 59

3.2 Simulating single cell data 62

3.3 Simulating bulk data 63

3.4 Parameters for synthetic data generation 64

3.5 Simulated genotypes from the GD model 64

4 Triple-negative breast cancer xenograft data **64**

4.1 Establishing the benchmark 65

5 Discarded data and potential effects on inference **66**

6 Sensitivity analysis **66**

6.1 Sensitivity to presence of doublets and sampling distortion noise 66

6.2 Sensitivity to a and noise 67

6.3 Sensitivity to the initial value of hyperparameter a 67

7 Data requirements for the method to function well **68**

7.1 Bulk data 68

7.2 Single cell data - number of cells 68

7.3 Single cell data - depth 68

8 Convergence diagnostics **69**

9 Parameter setting in method comparison experiments **69**

1 Supplementary figures

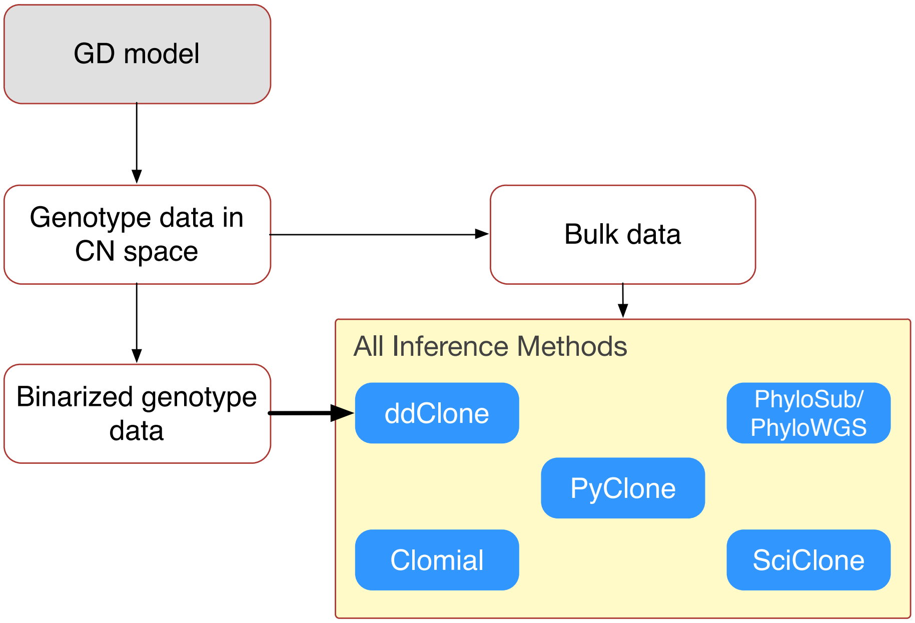

Figure S1: High-level data simulation workflow. First, the GD model is used to generate cell genotype data in copy number space (Δ^{CN}, i.e., number of reference and variant alleles for each cell genotype at each locus). Second, the cell genotype data is converted into bulk data. This is given as input to all the methods tested in this work to be used to infer the clustering assignment and cellular prevalences of genomic loci. Third, the cell genotype data in CN space is converted into a cell binary genotype matrix and supplied only to our method, ddClone, whereby it is used to construct an informed prior over the partitions of genomic loci (the bold arrow).

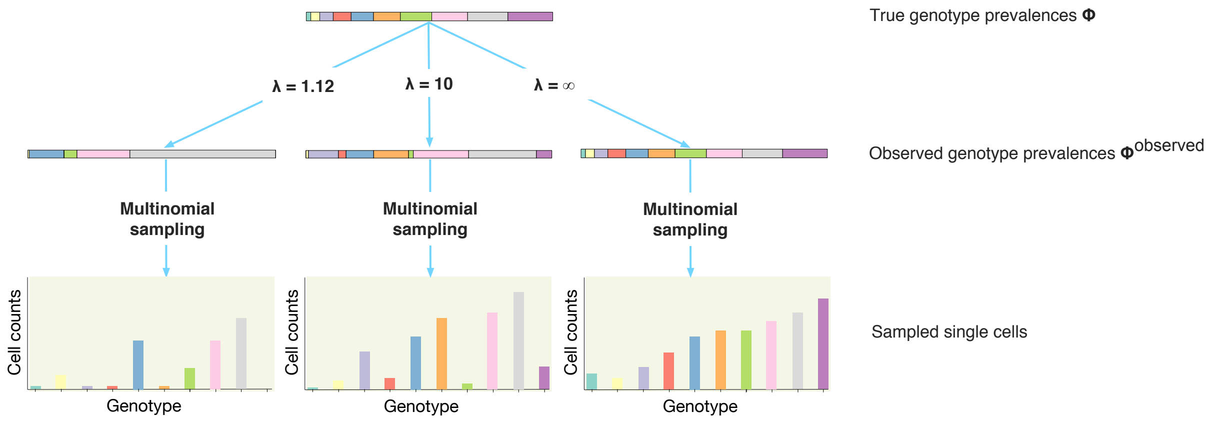

Figure S2: High-level single cell instantiation workflow for 3 different values of assortment bias. To simulate cells, we first sample observed prevalences $\Phi = \{\Phi_1^{\text{observed}}, \Phi_2^{\text{observed}}, ..., \Phi_M^{\text{observed}}\}$ for each genotype from a Dirichlet distribution $\Phi_{\text{observed}} \sim \text{Dir}(\lambda\Phi)$, where $\Phi = \{\Phi_1, \Phi_2, ..., \Phi_M\}$ are the true prevalences for genotypes 1 to M. We then simulate m cells from a multinomial distribution with parameters Φ_{observed}, i.e., $(n_1, n_2, ..., n_M) \sim \text{Mult}(\Phi_{\text{observed}})$ where n_i is the number of cells that have genotype i. This process is equivalent to sampling the cells from a Dirichlet-multinomial distribution, that is, $(n_1, n_2, ..., n_M) \sim$ Dirichlet-multinomial$(\lambda\Phi)$. The larger the λ is, the closer are the two vectors Φ_{observed} and Φ. In fact as the value of λ grows, the Dirichlet-multinomial distribution progressively better approximates the Multinomial distribution.

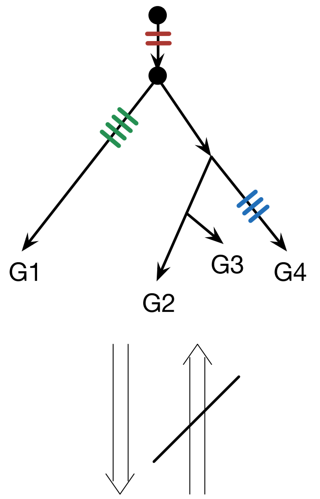

	G1	G4
**Mut 1**	x	
**Mut 2**	x	
**Mut 3**	x	
**Mut 4**	x	
**Mut 5**		x
**Mut 4**		x
**Mut 7**		x
**Mut 8**	x	x
**Mut 9**	x	x

Figure S3: A hypothetical phylogenetic tree with genotypes at leaves (top). The green and blue bars on the tree denote mutations that have happened together. A subset of the corresponding mutation co-occurrence patterns (bottom). Note that the bottom matrix shows a transposed version of the genotype matrix. While it always holds that if mutations are gained at the same site on the phylogenetic tree, then they will co-appear in the genotype matrix (the top-to-bottom arrow), the opposite is not always true (the bottom-to-top arrow). For instance, if all the mutations were ancestral, and G1 were to lose mutations 5 to 7, and similarly, G4 were to lose mutations 1 to 4, we would still observe the same genotype matrix as in this figure, but the underlying phylogenetic tree would be completely different, comprising a trunk. We are making the simplifying assumption that if mutations co-occur in a genotype matrix, then they have co-occurred in the underlying phylogenetic tree as well.

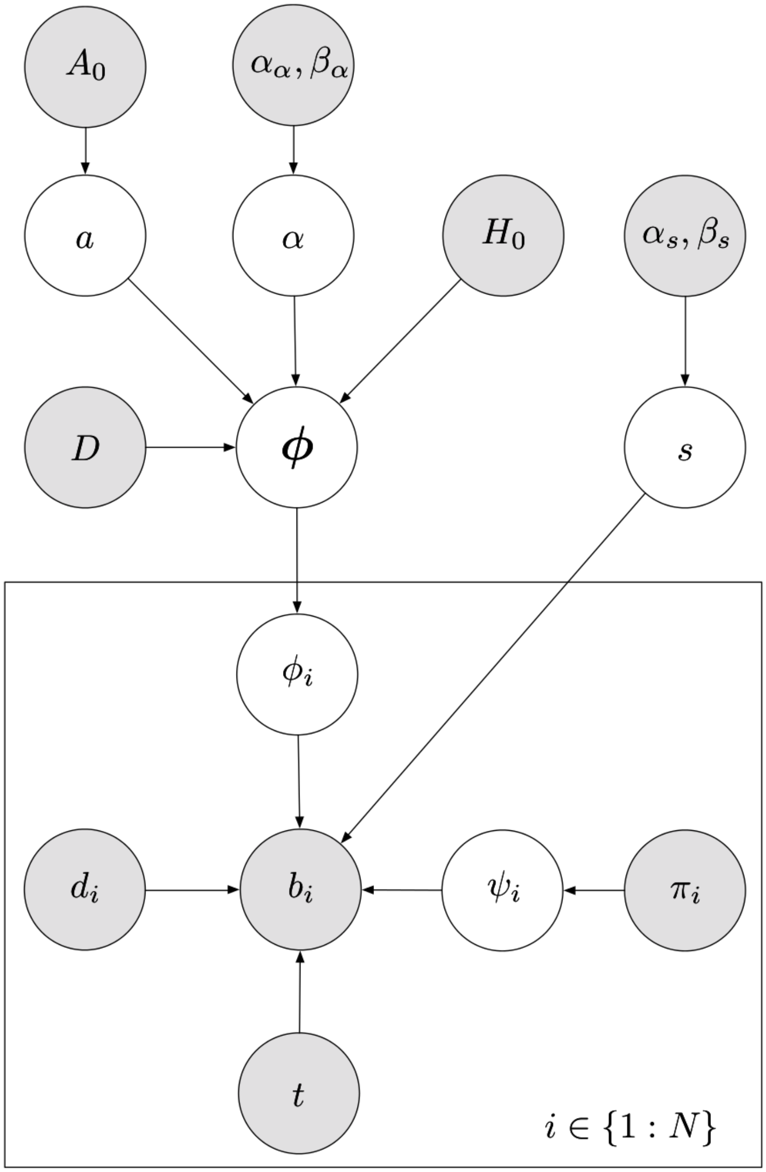

Figure S4: In the graphical model, the shaded nodes are observed and the rest of the nodes are not observed. In the inference step, the unobserved nodes will be inferred via Gibbs sampling. In particular, we are interested in inferring ϕ_i-s, the cellular prevalences for genomic loci and the induced clustering by the ddCRP.

$$\alpha \sim \text{Gamma}(a_\alpha, b_\alpha)$$

$$H_0 = \text{Uniform}([0,1])$$

$$A_0 = \text{Exp}(\lambda_0)$$

$$a \sim A_0$$

$$D = \{d_{i,j}\}, d_{i,j} = \text{JaccardDist}(i,j), i,j \in \{1:N\}$$

$$f_a = \exp(-d_{i,j}/a)$$

$$\boldsymbol{\phi} | f_a, D, H_0, \alpha \sim \text{ddCRP}(f_a, D, H_0, \alpha)$$

$$\psi_i | \pi_i \sim \text{Categorical}(\pi_i)$$

$$s | a_s, b_s \sim \text{Gamma}(a_s, b_s)$$

$$b_i | d_i, \psi_i, \phi_i, t, s \sim \text{BetaBinomial}(d_i, \xi(\psi_i, \phi_i, t), s)$$

$$\xi(\psi, \phi, t) = \frac{(1-t)\zeta(g_N)}{Z}\mu(g_N) + \frac{t(1-\phi)\zeta(g_R)}{Z}\mu(g_R) + \frac{t\phi\zeta(g_V)}{Z}\mu(g_V)$$

$$Z = (1-t)\zeta(g_N) + t(1-\phi)\zeta(g_R) + t\phi\zeta(g_V)$$

Figure S5: The distributional assumptions on the ddClone model. These random variables are described in more detail in table S1

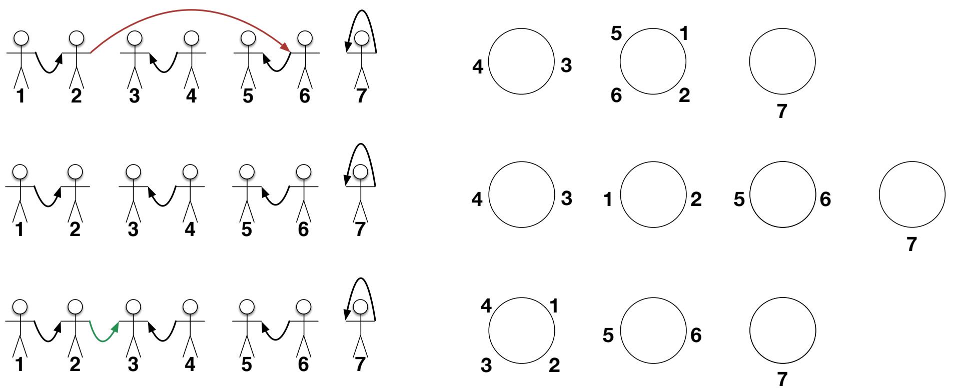

Figure S6: Possible moves taken by the sampler. Left column shows customer connections and right column shows induced table configuration at each step. Consider the following example showing resampling of the second customer. We first remove the outgoing connection of the customer, i.e., $c_2 = 6$ (top row, the red arrow). When this connection is removed, the second table is split into two tables, with customers one and two sitting at one table and customers five and six sitting at a new table (middle row). Customer three is picked as the new connection for customer two, i.e., $c_i^{\text{new}} = 3$, and this causes their respective tables to merge (bottom row, the green arrow).

figureS51JaccardDistRealData.pdf

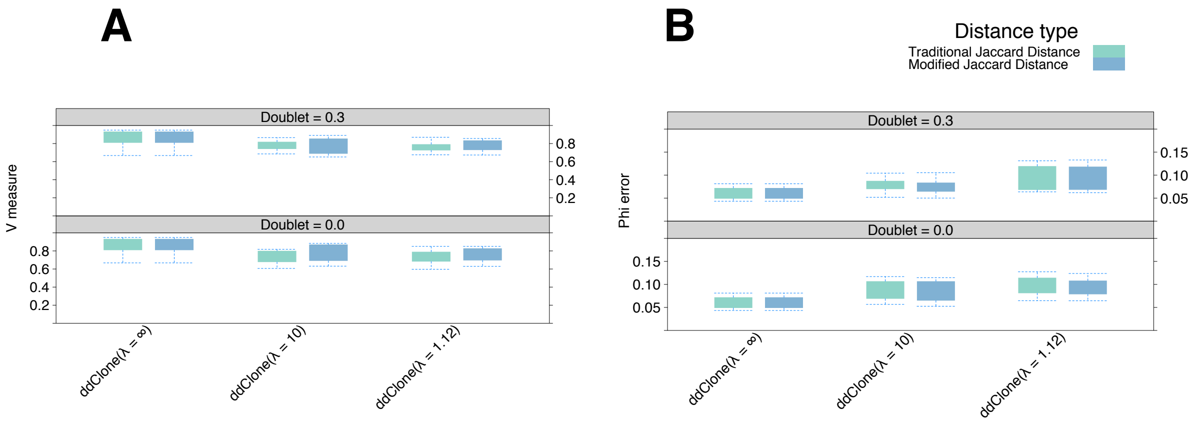

Figure S7: Comparing traditional and modified Jaccard distance over 10 simulated datasets in presence of ADO rate of 0.3. The data is identical to that in Figure 3 of the manuscript (Doublet = 0.3) and a configuration without doublets (Doublet = 0.0). The doublet rate is included at the top of each panel.

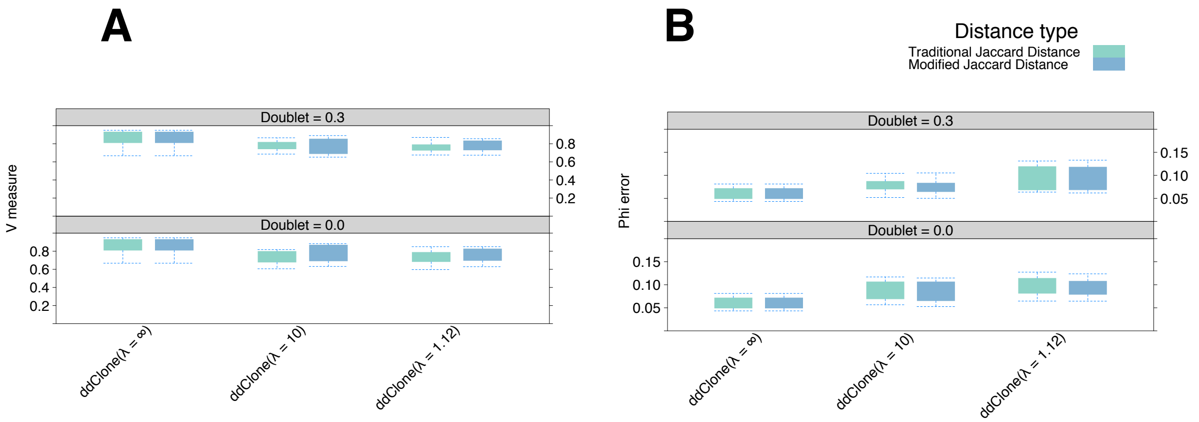

Figure S8: Comparing traditional and modified Jaccard distance over real datasets, suggesting small improvements with respect to both clustering and cellular prevalence values. ddClone with traditional and standard Jaccard distance had very similar results over the CLL data where compared to one another, the clusterings on average had a V measure of 0.81 and cellular prevalence estimation difference of only 0.010.

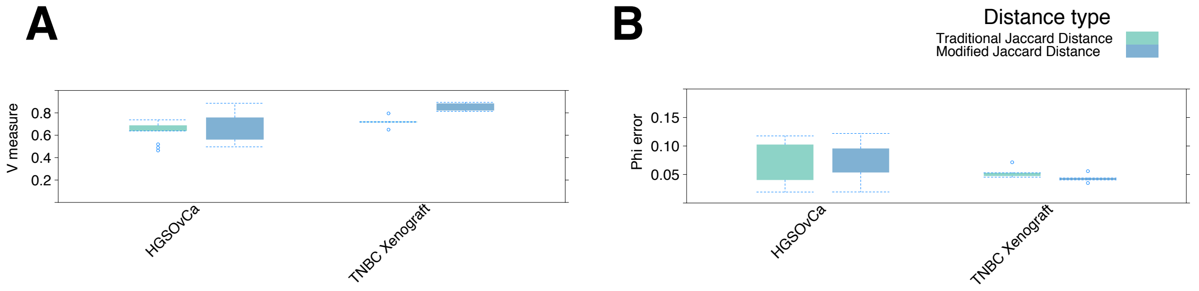

Figure S9: Possible moves taken by the sampler.

	(0,0)	(1,0)	(2,0)
(0,0)	0	0	0
(1,0)	0	−1	1
(2,0)	0	1	−1

	(0,0)	(0,1)	(0,2)	(1,0)	(1,1)	(1,2)	(2,0)	(2,1)	(2,2)
(0,0)	0	0	0	0	0	0	0	0	0
(0,1)	0	−1	1	0	0	0	0	0	0
(0,2)	0	1	−1	0	0	0	0	0	0
(1,0)	0	0	0	−1	0	0	1	0	0
(1,1)	0	1	0	1	−4	1	0	1	0
(1,2)	0	0	1	0	1	−3	0	0	1
(2,0)	0	0	0	1	0	0	−1	0	0
(2,1)	0	0	0	0	1	0	1	−3	1
(2,2)	0	0	0	0	0	1	0	1	−2

Figure S10: Rate matrices for CTMC used on $\tau_{-p_{\mathrm{SNV}}}$ (left) and $\tau_{p_{\mathrm{SNV}}}$ (right). States represent are pairs representing reference and variant allele copy numbers. In this example, maximum allowed copy number for both reference and variant alleles is 2. States to which transition is possible are annotated green. Note that in both rate matrices, the first row and column (representing transitioning from and to the complete deletion state) are all zero. This means that it is not possible to reach complete deletion in our model.

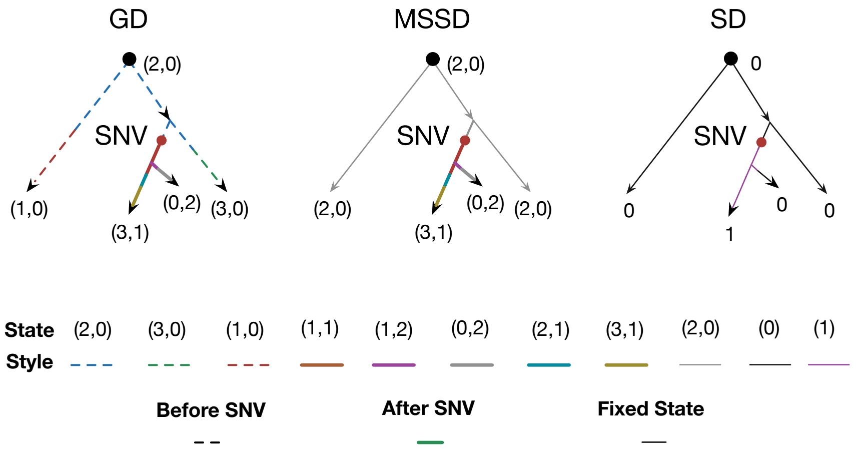

Figure S11: An instance of Generalized Dollo, Multi state stochastic Dollo and Stochastic Dollo models over a rooted phylogenetic tree for a single genomic locus side by side. We assume that a SNV has happened at the red dot on the tree. Dashed lines represent the GD model's run over the subtree before the SNV has happened. The thick solid lines represent the process after the SNV has happened. The thin solid lines represent a fixed state, i.e., the process can only handle a fixed state before the SNV gain event. The numbers and colours represent the state of the process (CTMC) at that point. GD can model multiple states on branches where SNV does not appear, while MSSD is forced to be in a fixed state in those positions. Hence the space of problems that GD models is a superset of that of SD.

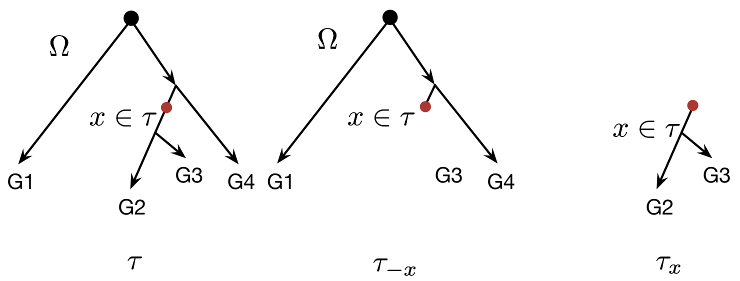

Figure S12: A rooted tree topology τ with root node Ω and SNV event at point x (left). $G1$, $G2$, $G3$, and $G4$ represent genotypes. τ_{-x} is the subtree pruned at x (middle). Subtree rooted at x is denoted by τ_x(right). To simulate from the Generalized Dollo model, for a specific genomic locus i, we first pick the SNV position on the tree, then simulate a CTMC on the pruned tree, and simulate another CTMC on the subtree.

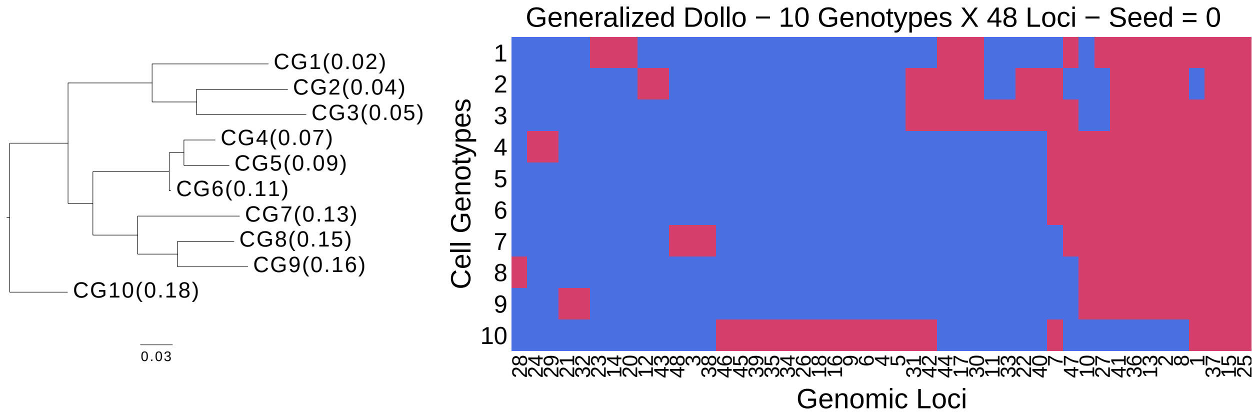

Figure S13: Transposed binarized simulated cell genotypes Δ from Generalized Dollo process over a fixed phylogeny. The original cell genotype matrix Δ^{CN} is in copy number space. We binarize it by setting entries with non zero variant allele copy number to one (coloured red) and setting entries with variant allele copy number of zero to zero (coloured blue). The clonal prevalence of each genotype is in parenthesis.

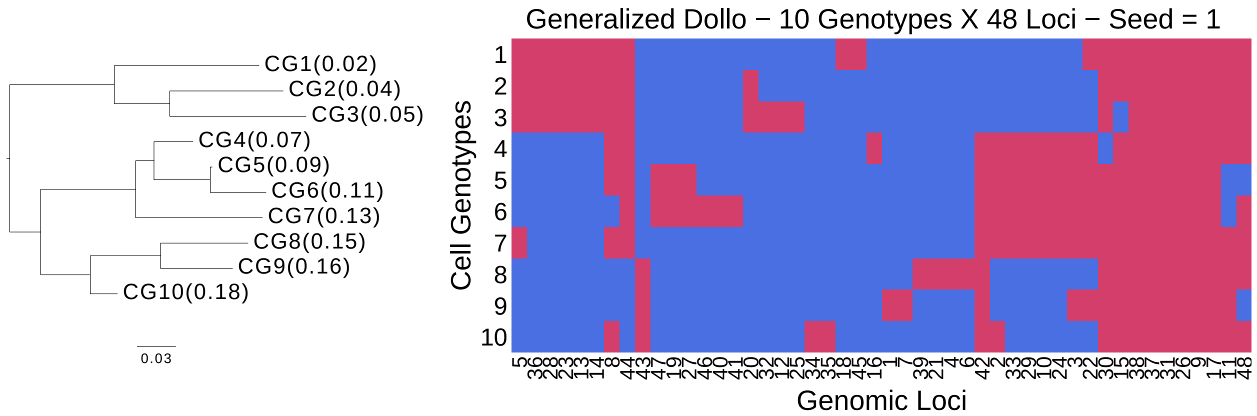

Figure S14: Transposed binarized simulated cell genotypes Δ from Generalized Dollo process over a fixed phylogeny. The original cell genotype matrix Δ^{CN} is in copy number space. We binarize it by setting entries with non zero variant allele copy number to one (coloured red) and setting entries with variant allele copy number of zero to zero (coloured blue). The clonal prevalence of each genotype is in parenthesis.

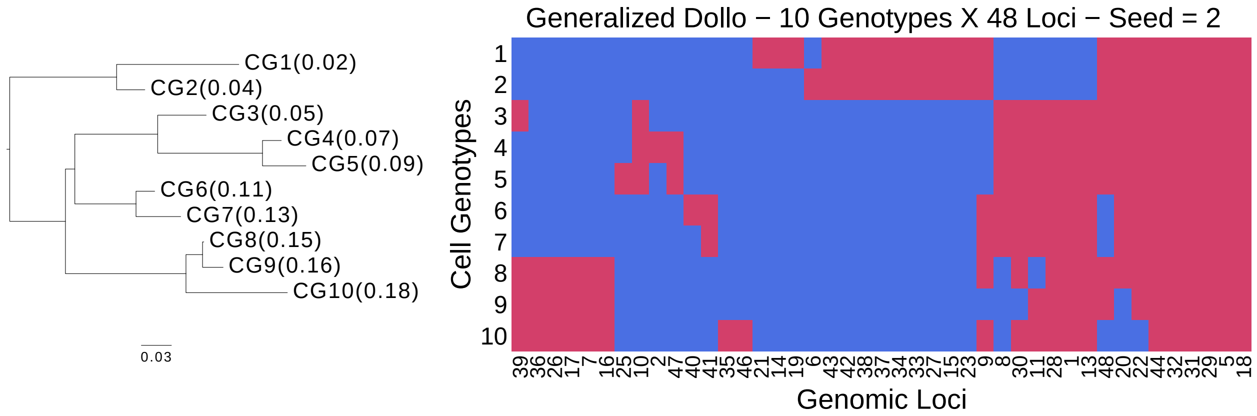

Figure S15: Transposed binarized simulated cell genotypes Δ from Generalized Dollo process over a fixed phylogeny. The original cell genotype matrix Δ^{CN} is in copy number space. We binarize it by setting entries with non zero variant allele copy number to one (coloured red) and setting entries with variant allele copy number of zero to zero (coloured blue). The clonal prevalence of each genotype is in parenthesis.

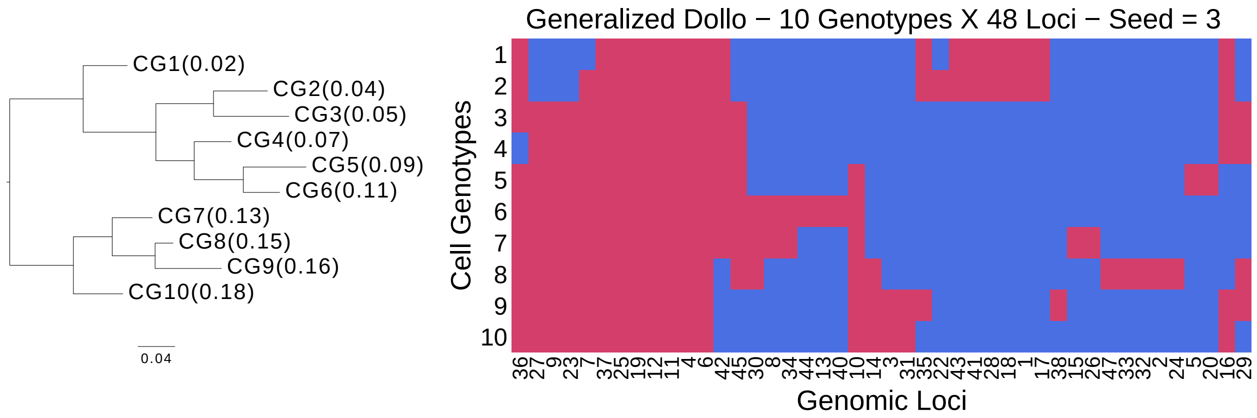

Figure S16: Transposed binarized simulated cell genotypes Δ from Generalized Dollo process over a fixed phylogeny. The original cell genotype matrix Δ^{CN} is in copy number space. We binarize it by setting entries with non zero variant allele copy number to one (coloured red) and setting entries with variant allele copy number of zero to zero (coloured blue). The clonal prevalence of each genotype is in parenthesis.

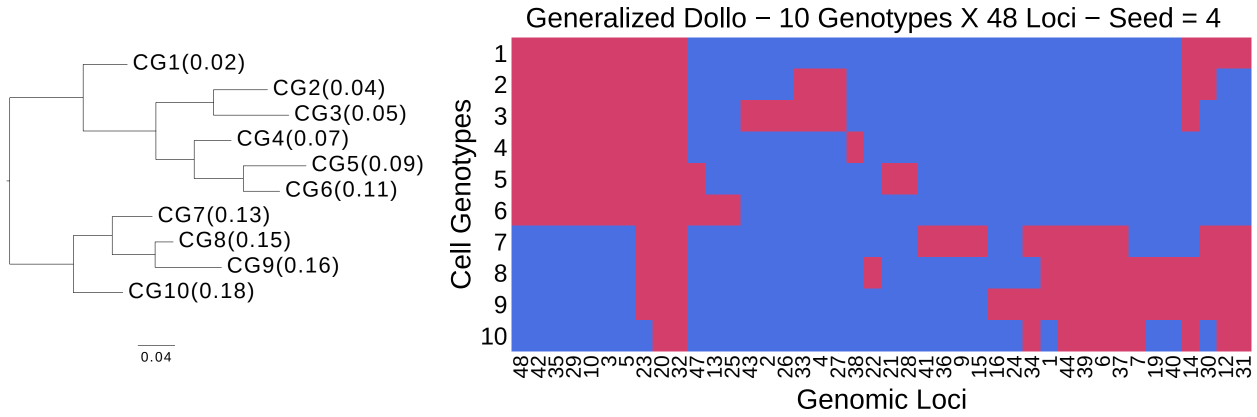

Figure S17: Transposed binarized simulated cell genotypes Δ from Generalized Dollo process over a fixed phylogeny. The original cell genotype matrix Δ^{CN} is in copy number space. We binarize it by setting entries with non zero variant allele copy number to one (coloured red) and setting entries with variant allele copy number of zero to zero (coloured blue). The clonal prevalence of each genotype is in parenthesis.

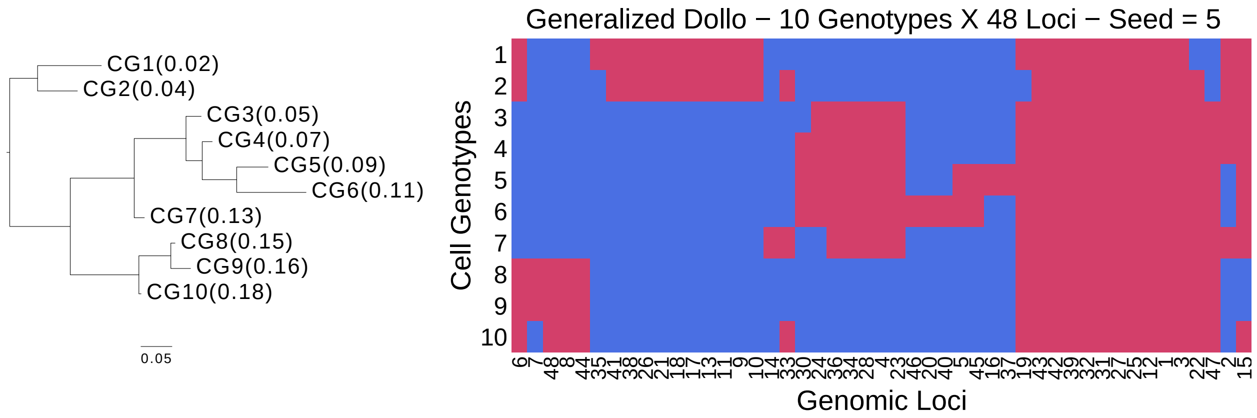

Figure S18: Transposed binarized simulated cell genotypes Δ from Generalized Dollo process over a fixed phylogeny. The original cell genotype matrix Δ^{CN} is in copy number space. We binarize it by setting entries with non zero variant allele copy number to one (coloured red) and setting entries with variant allele copy number of zero to zero (coloured blue). The clonal prevalence of each genotype is in parenthesis.

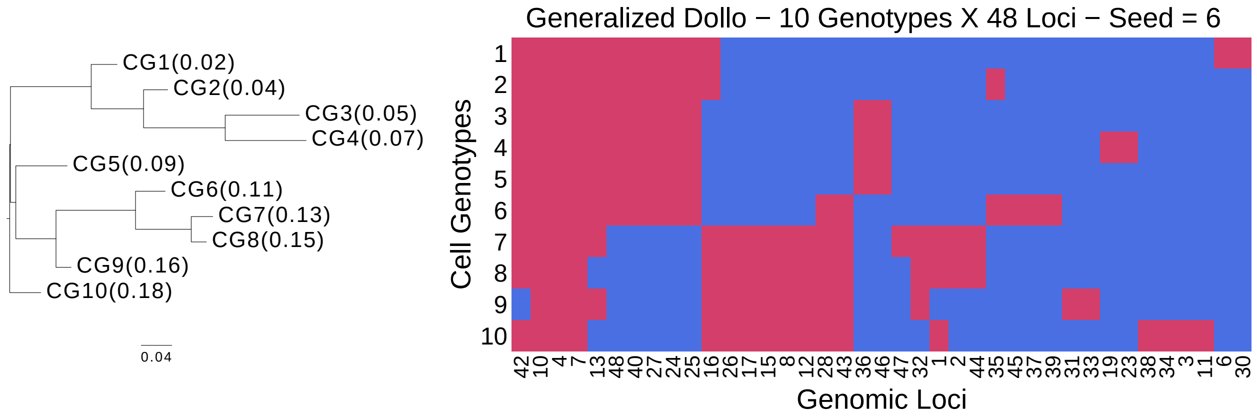

Figure S19: Transposed binarized simulated cell genotypes Δ from Generalized Dollo process over a fixed phylogeny. The original cell genotype matrix Δ^{CN} is in copy number space. We binarize it by setting entries with non zero variant allele copy number to one (coloured red) and setting entries with variant allele copy number of zero to zero (coloured blue). The clonal prevalence of each genotype is in parenthesis.

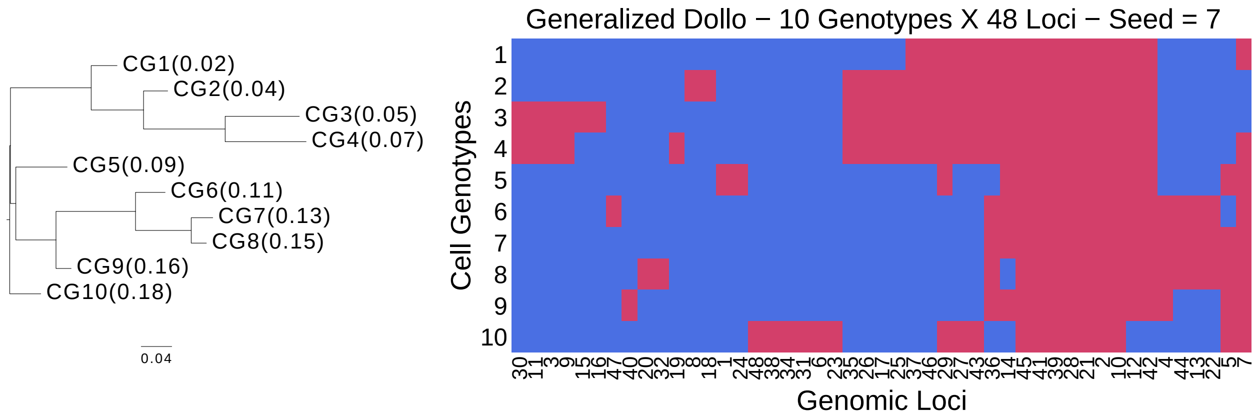

Figure S20: Transposed binarized simulated cell genotypes Δ from Generalized Dollo process over a fixed phylogeny. The original cell genotype matrix Δ^{CN} is in copy number space. We binarize it by setting entries with non zero variant allele copy number to one (coloured red) and setting entries with variant allele copy number of zero to zero (coloured blue). The clonal prevalence of each genotype is in parenthesis.

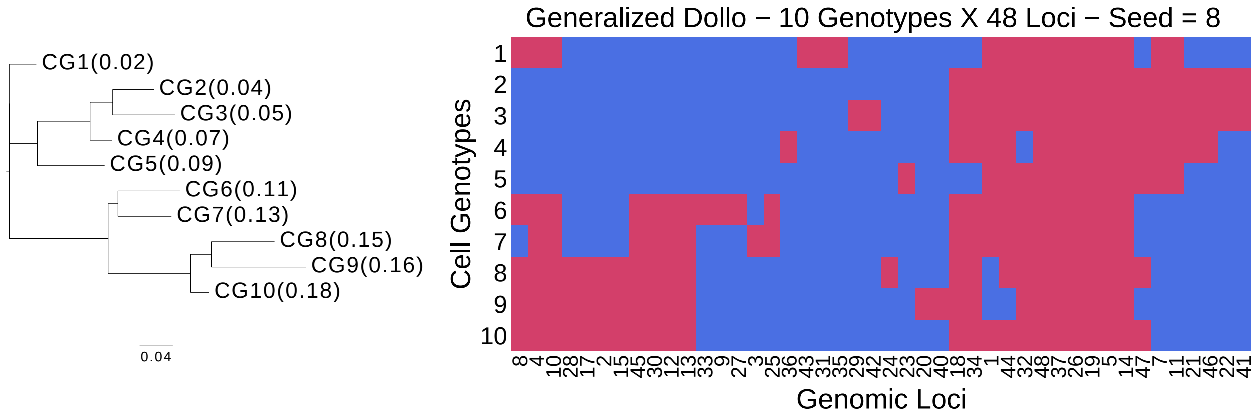

Figure S21: Transposed binarized simulated cell genotypes Δ from Generalized Dollo process over a fixed phylogeny. The original cell genotype matrix Δ^{CN} is in copy number space. We binarize it by setting entries with non zero variant allele copy number to one (coloured red) and setting entries with variant allele copy number of zero to zero (coloured blue). The clonal prevalence of each genotype is in parenthesis.

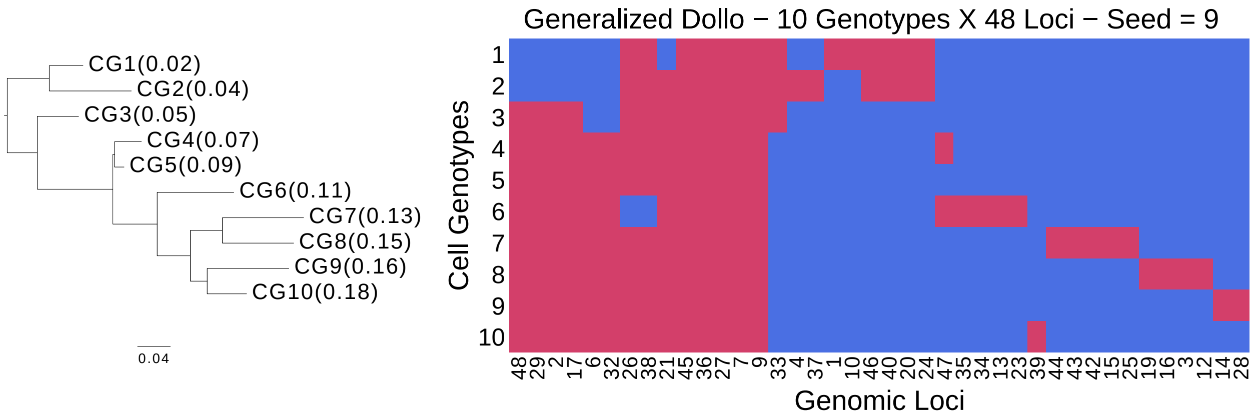

Figure S22: Transposed binarized simulated cell genotypes Δ from Generalized Dollo process over a fixed phylogeny. The original cell genotype matrix Δ^{CN} is in copy number space. We binarize it by setting entries with non zero variant allele copy number to one (coloured red) and setting entries with variant allele copy number of zero to zero (coloured blue). The clonal prevalence of each genotype is in parenthesis.

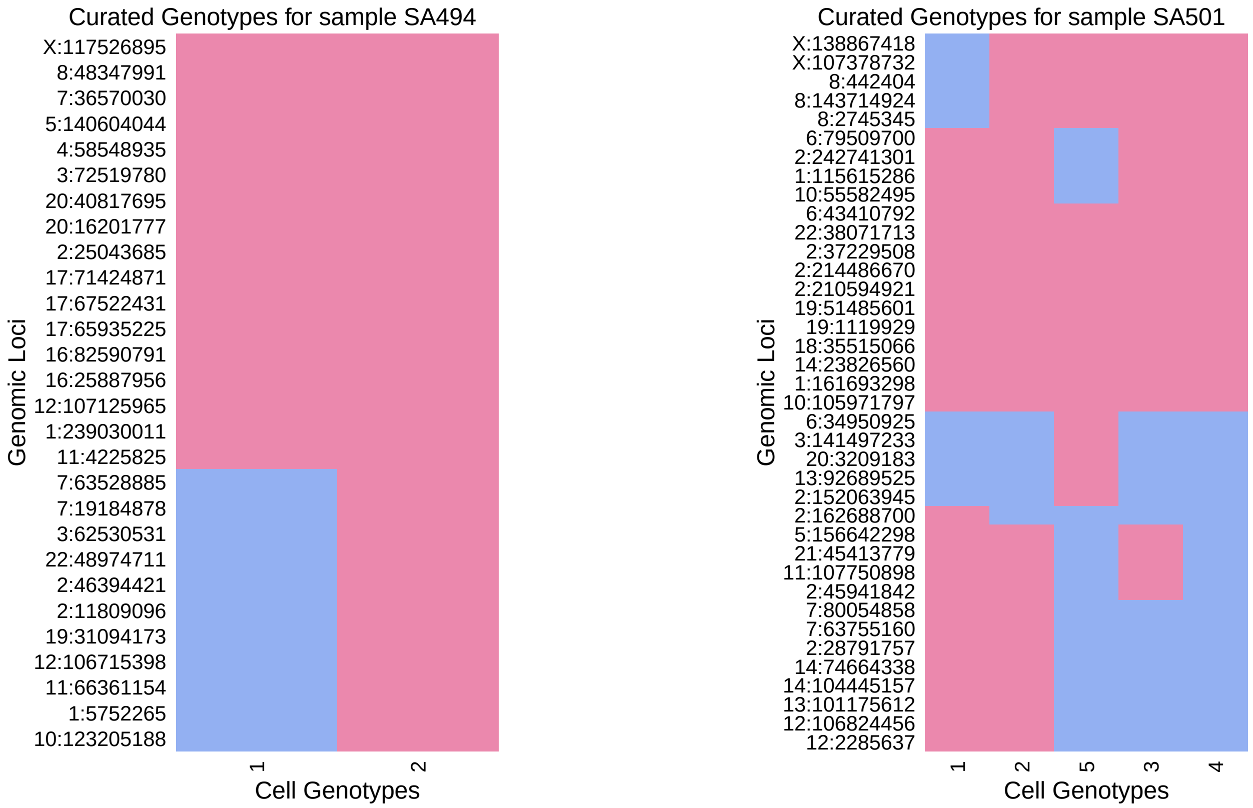

Figure S23: Binary cell genotype matrices for sample SA494 over 29 genomic loci (left) and sample SA501 over 38 genomic loci (right). These are manually curated from a single cell genotype sequencing experiment [1]. Briefly, MrBayes was used to infer a consensus phylogenetic tree over the single nuclei. Then they were grouped into clades according to high probability branching splits. Finally, each clade was assigned a consensus cell genotype by taking the mode cell genotype of the clade at each genomic locus.

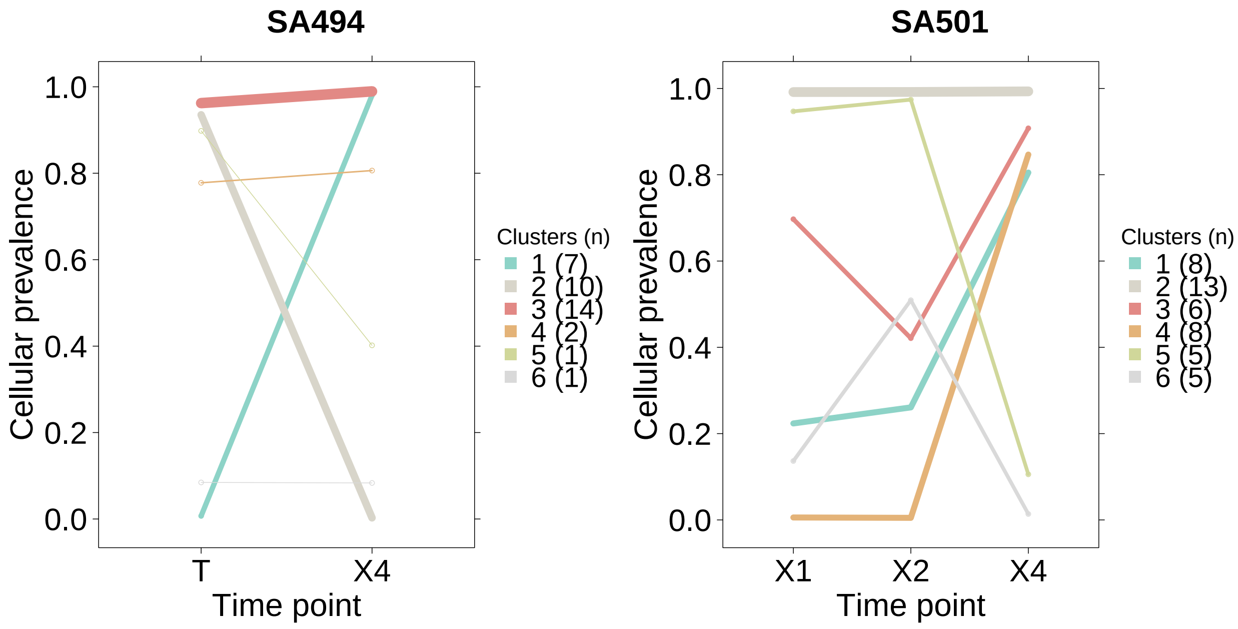

Figure S24: Clustering result for multi-sample PyClone over timepoints `SA501 X1, X2, X4`, and `SA494 T, X4` for genomic loci that overlap with those sequenced in the single genotype analysis. The number of mutations assigned to each cluster is shown in parenthesis.

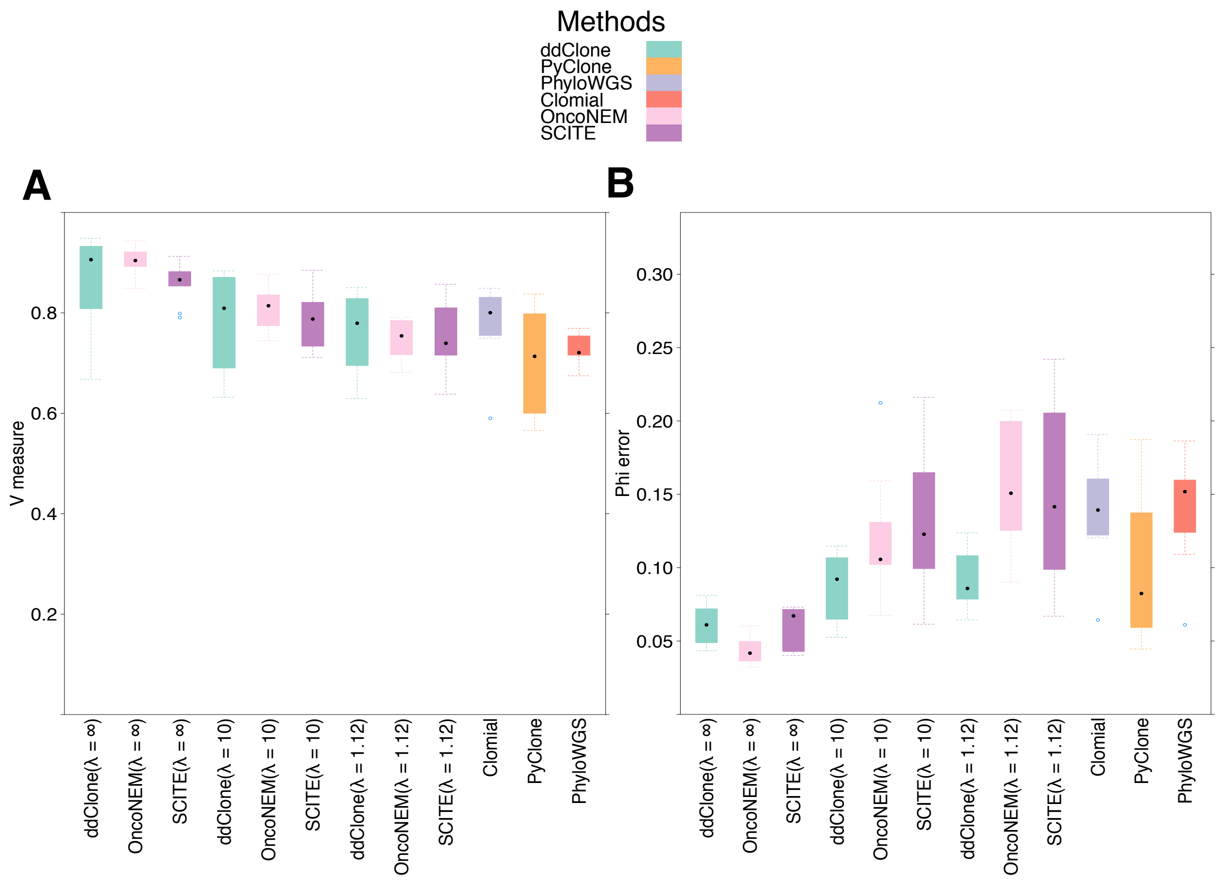

Figure S25: Benchmarking over simulated data with NO doublets. The rest of the parameters are identical to that of Figure 3 in the main manuscript. This result corroborates our previous results, that is, the ddClone model performs better or comparably well in presence of reasonable levels of noise. Panel A shows V-measure clustering performance. Panel B shows the average over loci of the absolute differences between the inferred and true cellular prevalences.

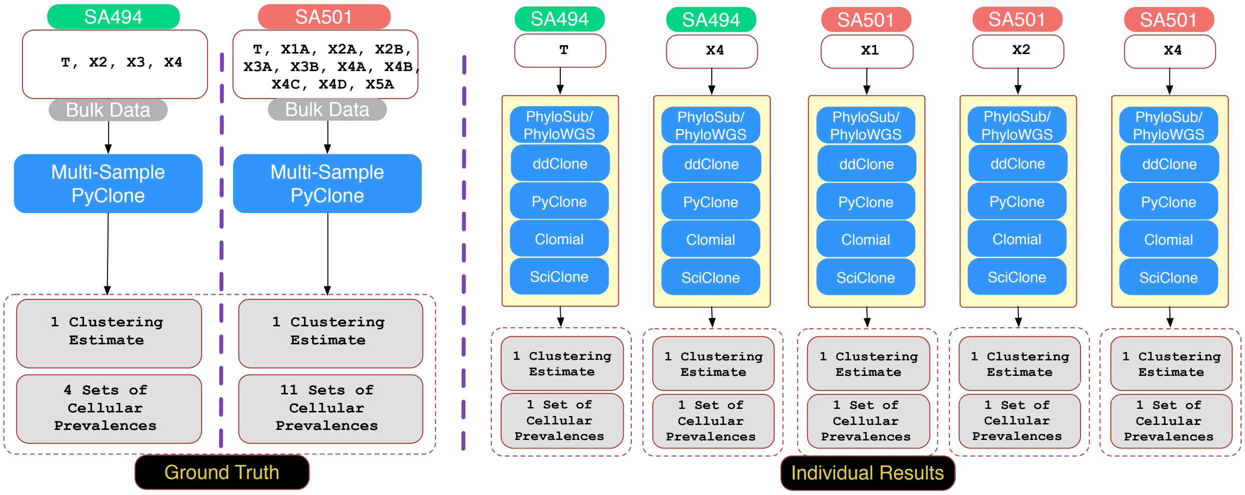

Figure S26: To establish the benchmark in the Xenograft dataset, PyClone was run in multi-sample mode once over 4 timepoints in sample SA494 and once over 11 timepoints in sample SA591 (left) Then each method was run over individual timepoints (right). ddClone was provided with the matching single-cell genomic data. For evaluation, each individual run was compared against the corresponding sample from the benchmark.

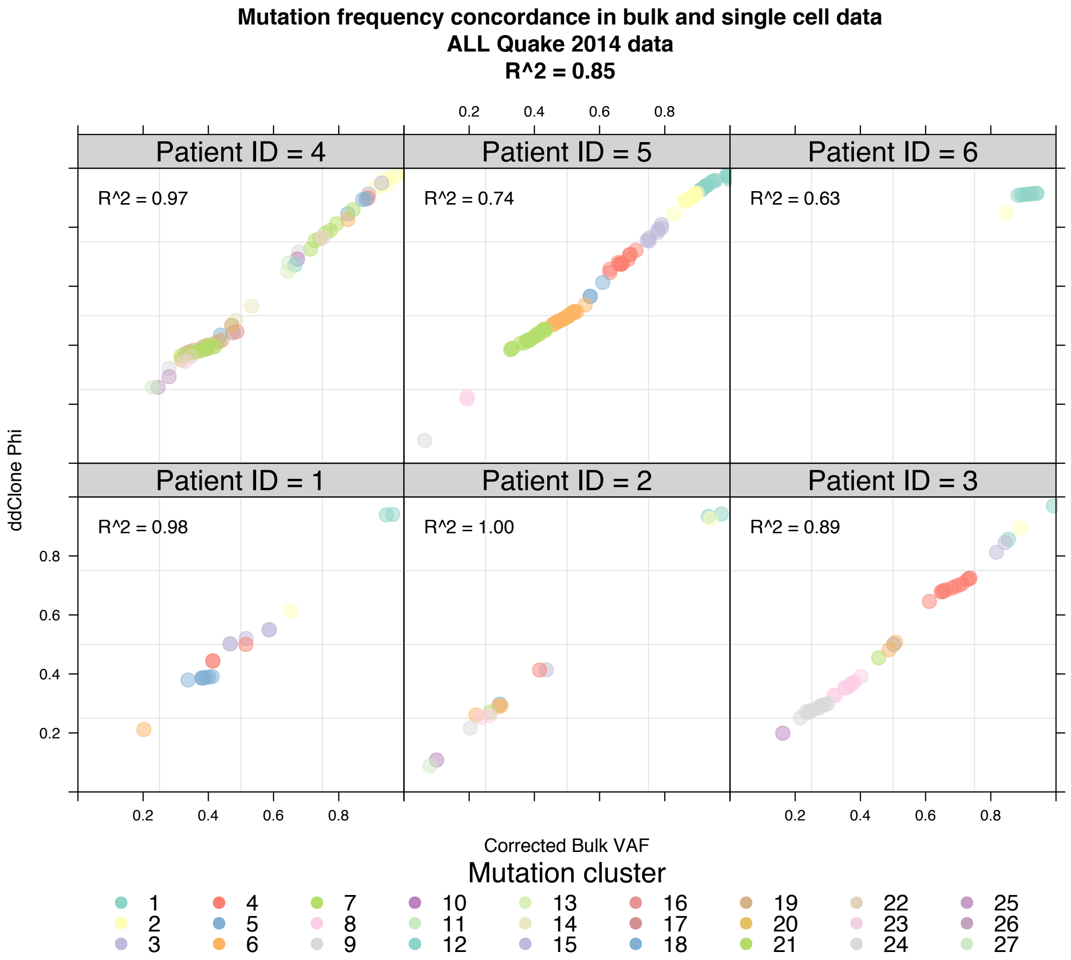

Figure S27: Concordance between mutation cellular prevalences estimated by ddClone and the corrected bulk VAF (multiplied by 2 for assuming diploidy)

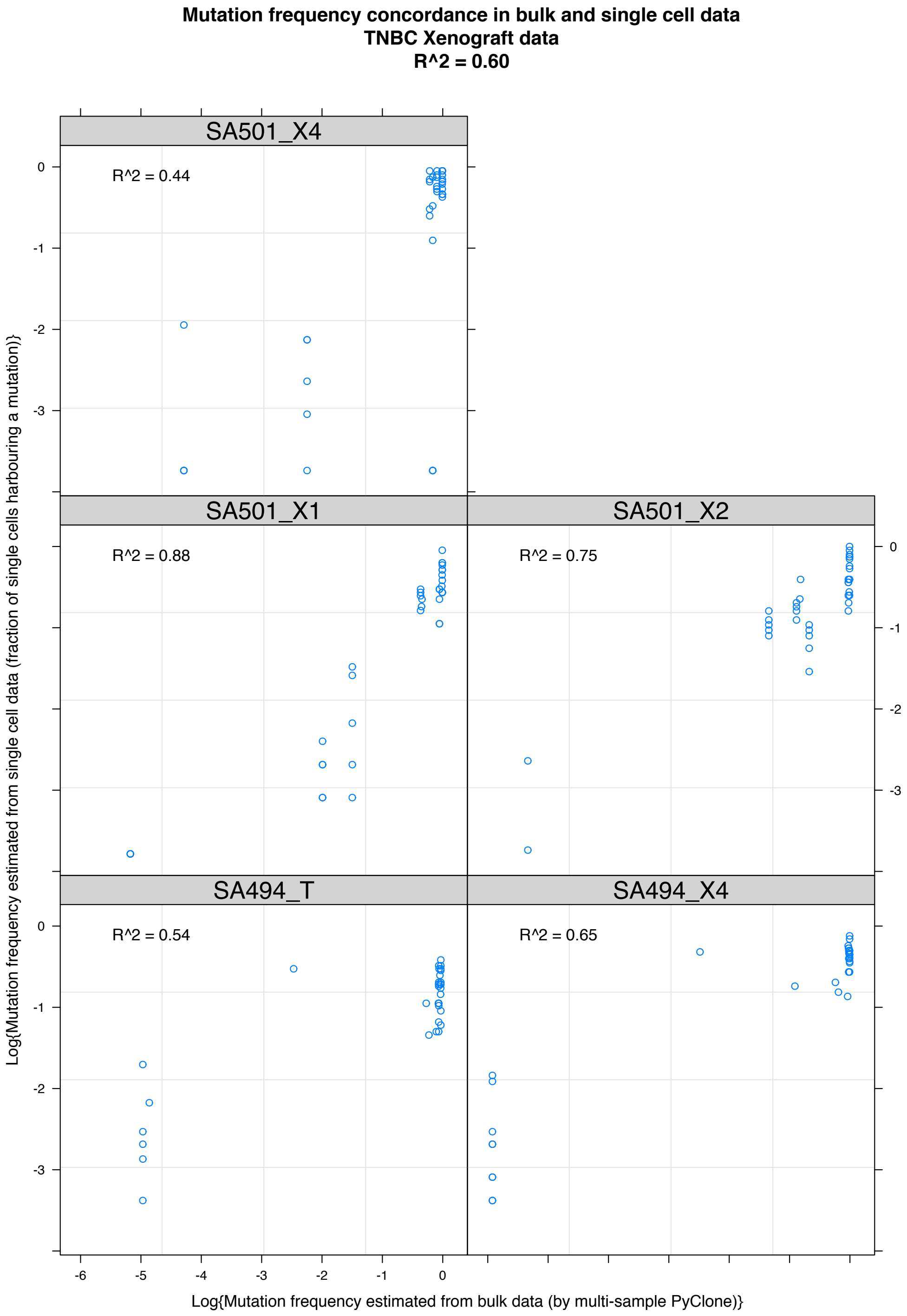

Figure S28: The R^2 above the plot is for pooling all mutations across all timepoints together. R^2 values for individual timepoints are shown on the upper left corner of their respective panels. The values range from 0.44 in SA501X4 to 0.88 in SA501X1.

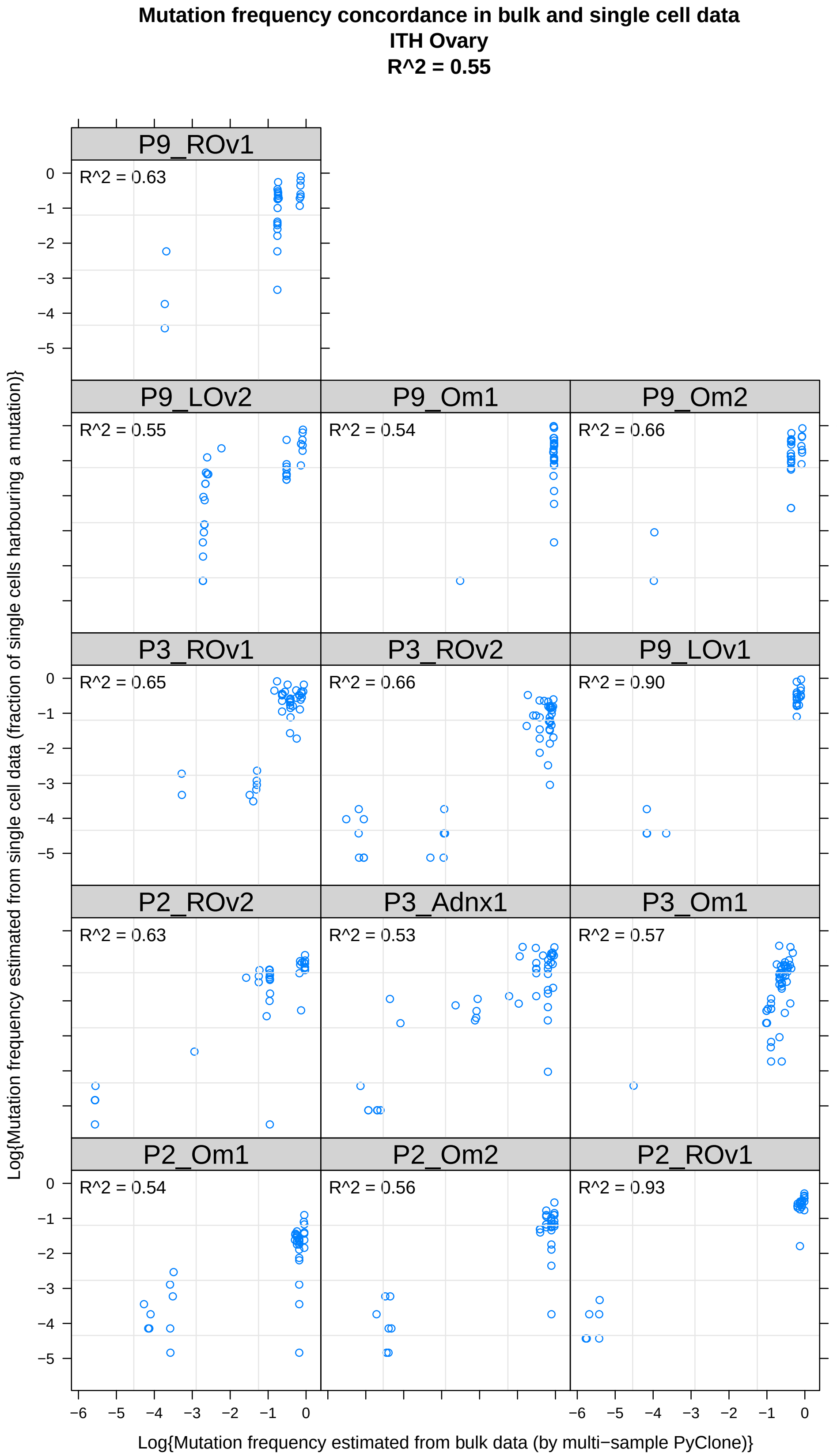

Figure S29: The R^2 above the plot is for pooling all mutations across all timepoints together. R^2 values for individual timepoints are shown on the upper left corner of their respective panels. The values range from 0.53 in P3Adnx1 to 0.93 in P2ROv1.

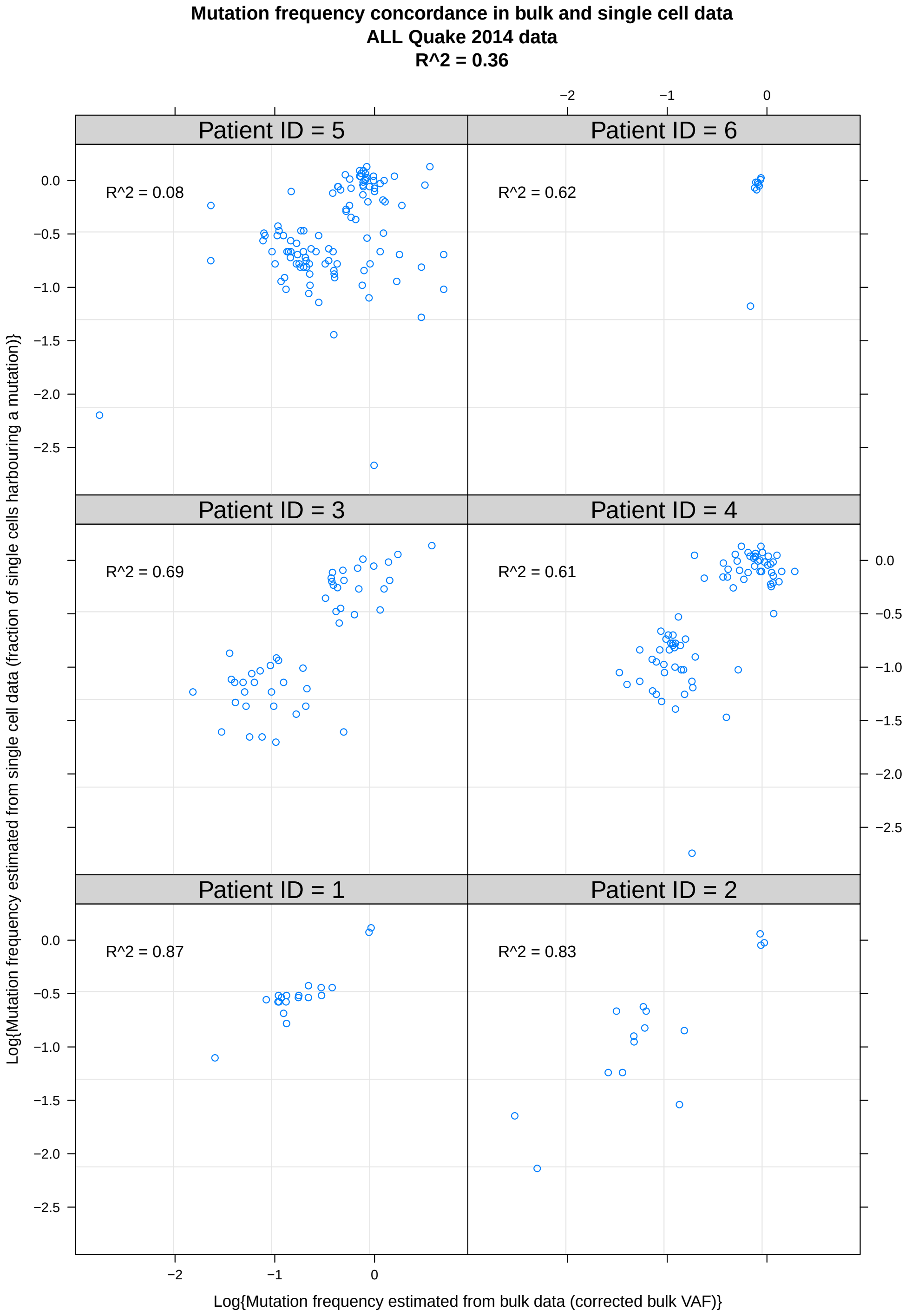

Figure S30: The R^2 above the plot is for pooling all mutations across all timepoints together. R^2 values for individual timepoints are shown on the upper left corner of their respective panels. The values range from 0.08 in Patient 5 to 0.87 in Patient 1.

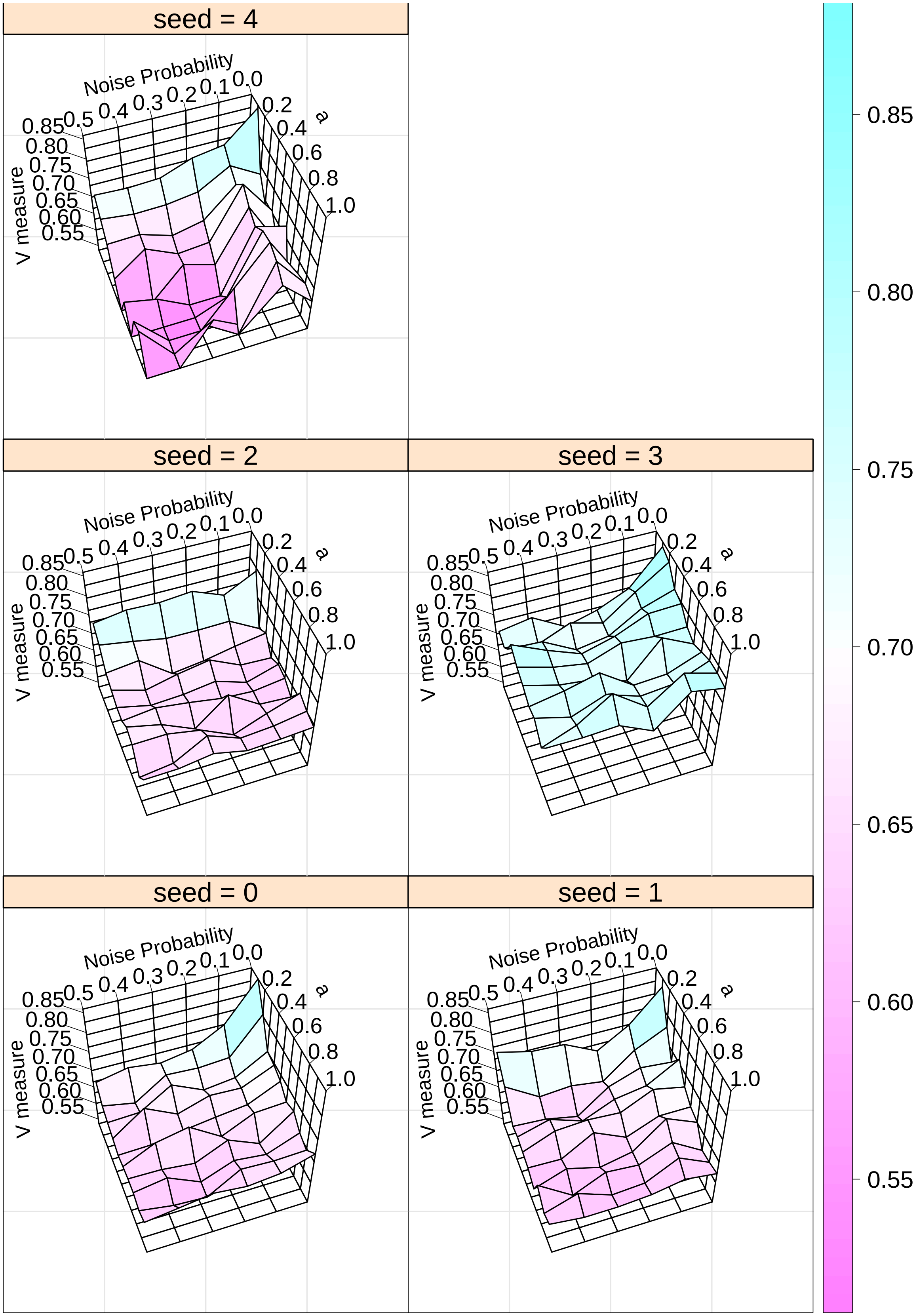

(a) V measure Index

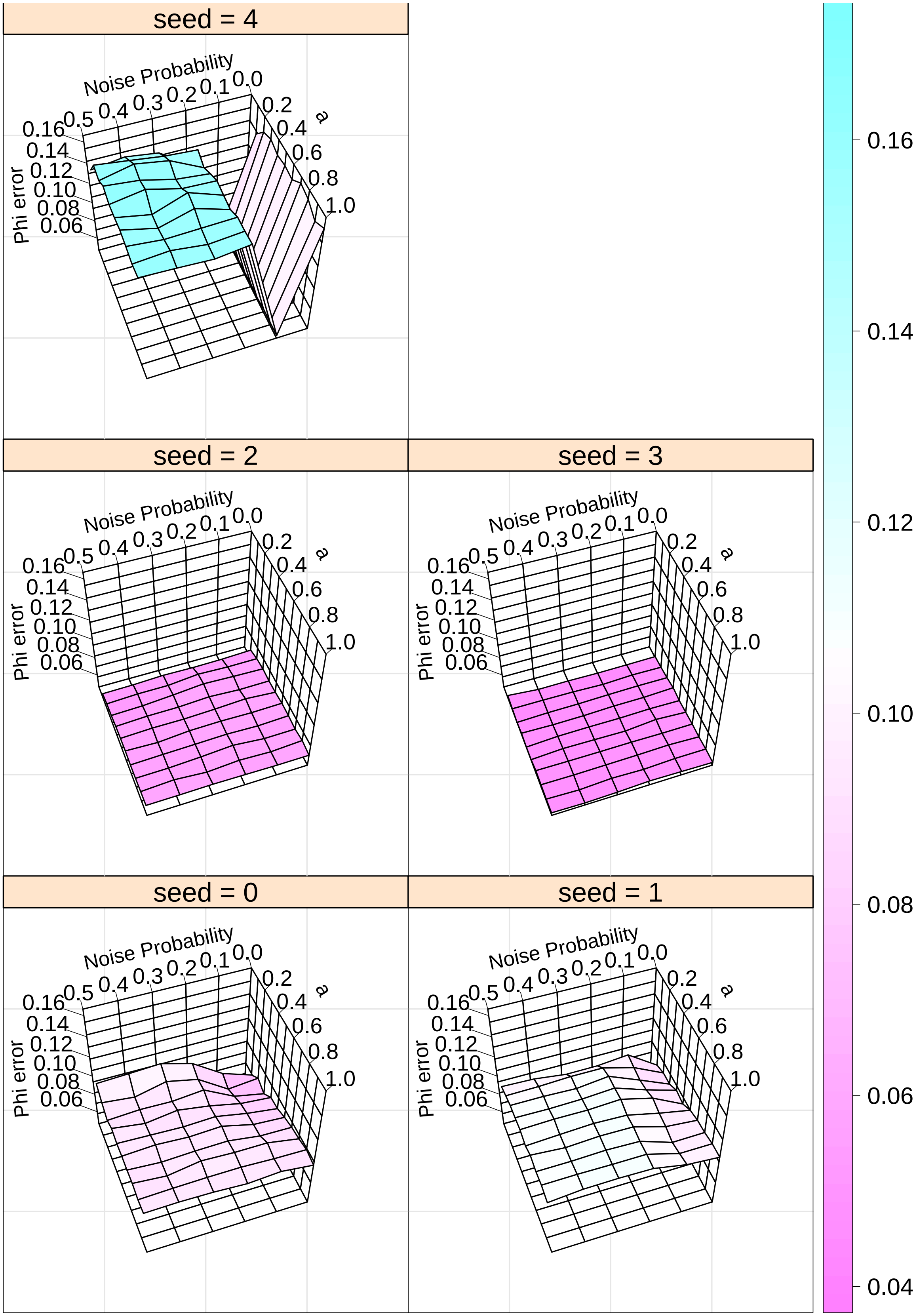

(b) Cellular prevalence error

Figure S31: Effect of adding random point noise and varying decay parameter a on V measure index (a) and mean absolute error of cellular prevalence estimates (b) for the five simulated datasets. Beta-Binomial precision parameter s and hyperparameter α are fixed at 1000 and 1 respectively. We note that V measure index is more sensitive to changes in value of a than the level of point noise. Heat map colours represent values in the vertical axis and are included to aid the eyes.

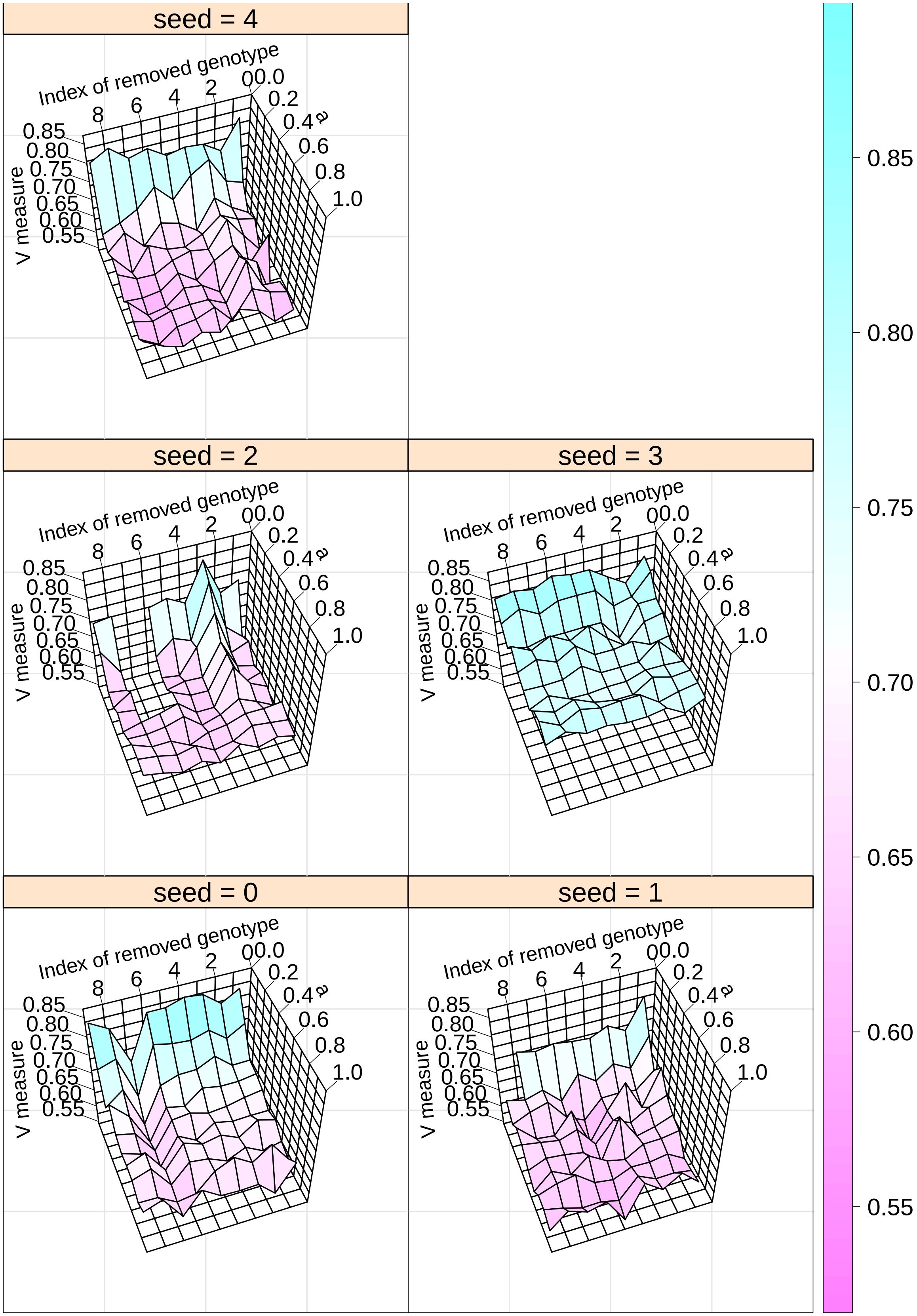

(a) V measure Index

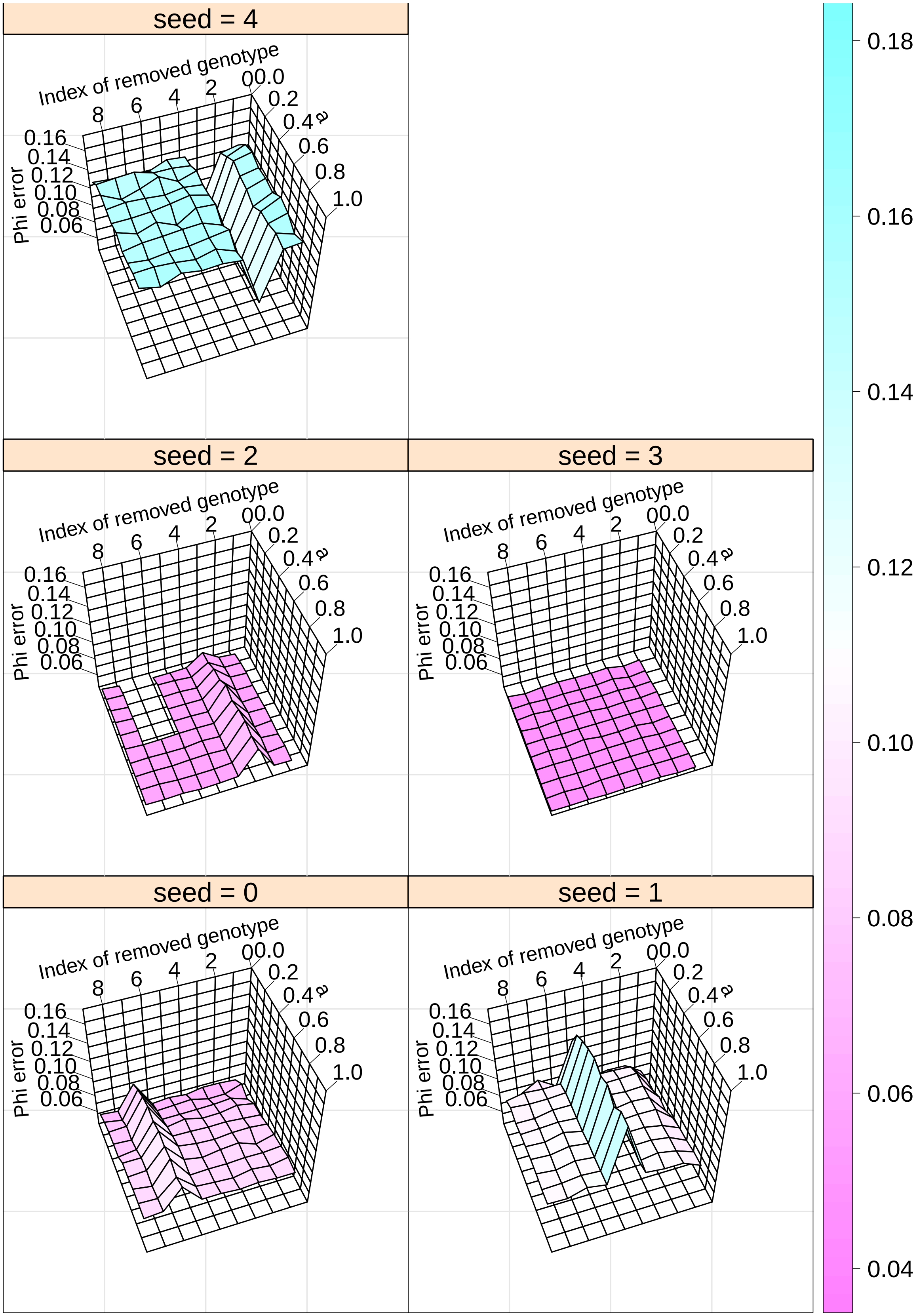

(b) Cellular prevalence error

Figure S32: Effect of removing single genotypes and varying hyperparameter a on V measure index (a) and mean absolute error of cellular prevalence estimates (b) for five simulated datasets. Genotypes are sorted in decreasing order of prevalence from right to left. Genotype 1 is the least prevalent and genotype 9 is the most prevalent. Beta-Binomial precision parameter s and hyperparameter α are fixed at 1000 and 1 respectively. We note that V measure index is more sensitive to changes in value of a than removal of single genotypes. Heat map colours represent values in the vertical axis and are included to aid the eyes.

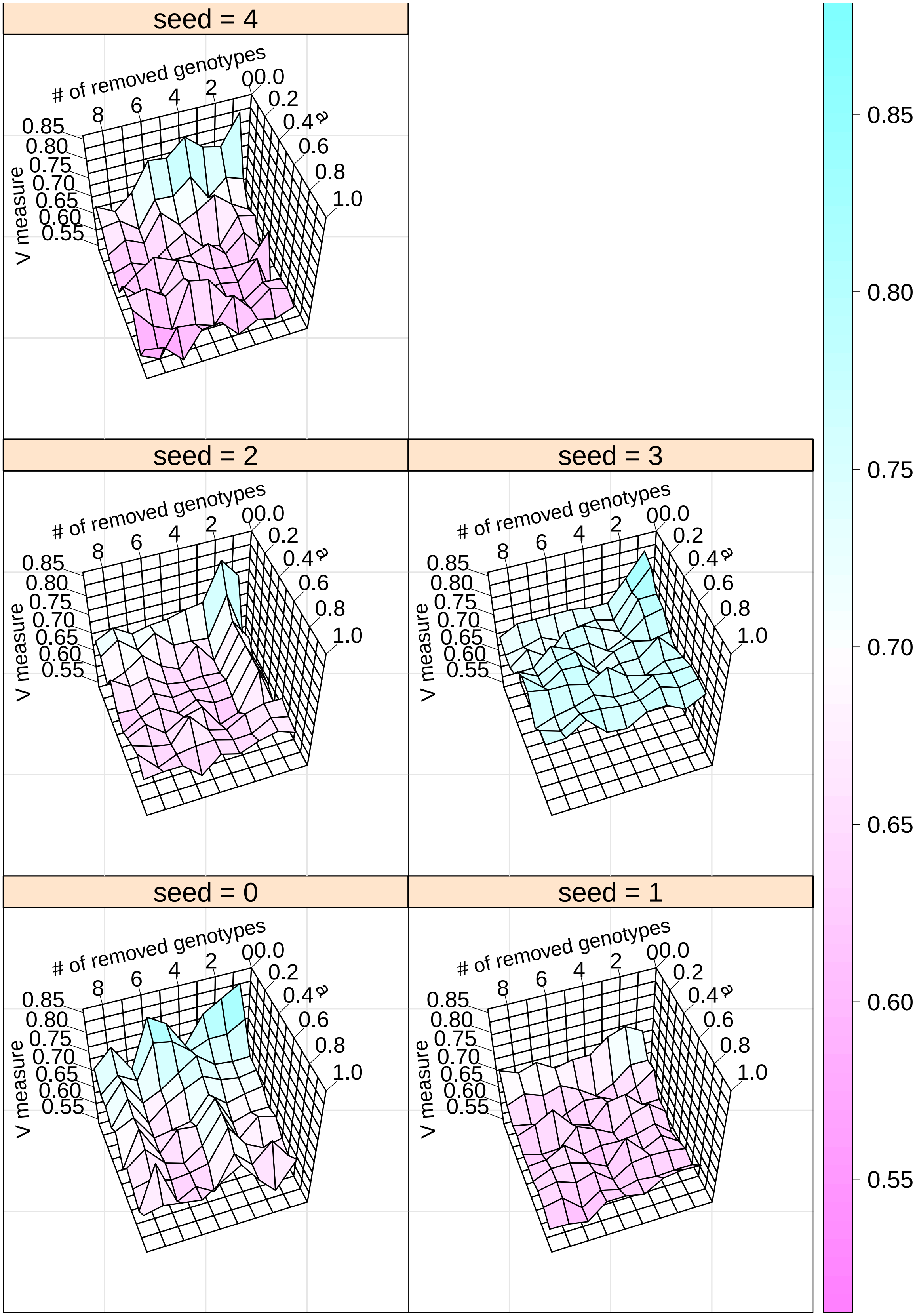

(a) V measure Index

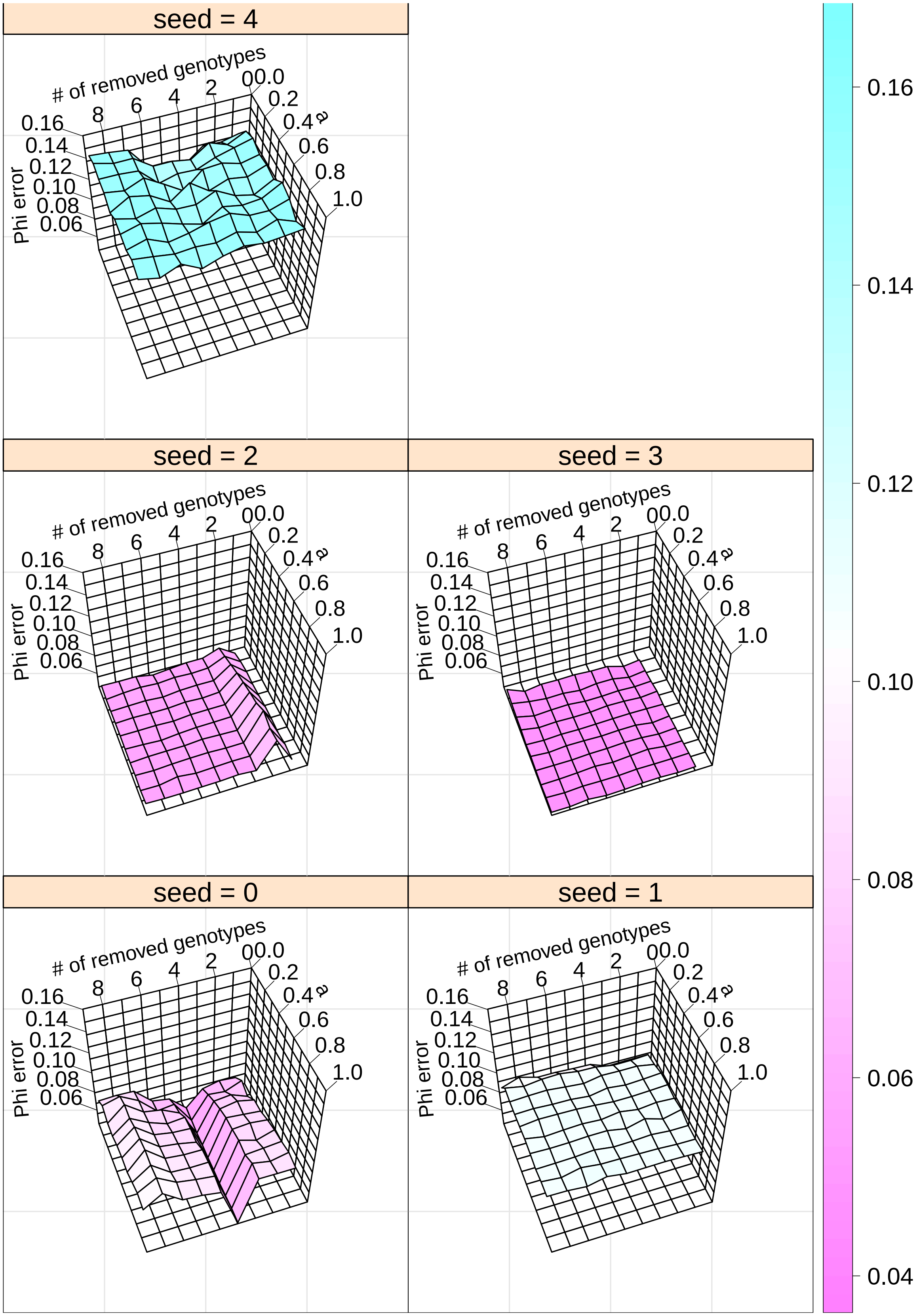

(b) Cellular prevalence error

Figure S33: Effect of removing progressively more genotypes and varying decay parameter a on V measure index (a) and mean absolute error of cellular prevalence estimates (b) for five simulated datasets. Beta-Binomial precision parameter s and hyperparameter α are fixed at 1000 and 1 respectively. We note that V measure index is more sensitive to changes in value of a than removal of multiple low prevalence genotypes. Heat map colours represent values in the vertical axis and are included to aid the eyes.

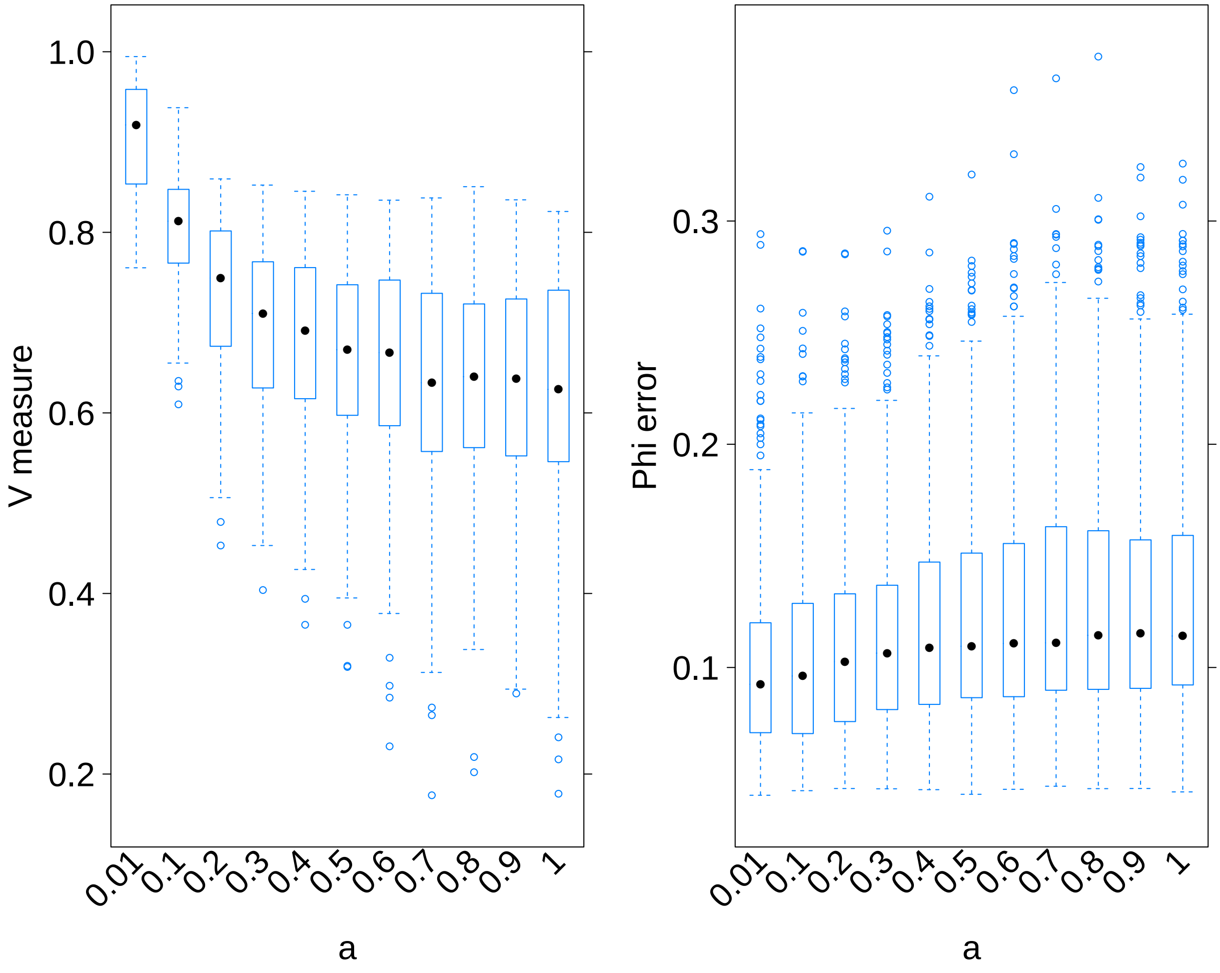

Figure S34: Performance over 10 synthetic datasets. Hyperparameter a is fixed at the specific value for each inference run. This result suggests that performance declines with increasing values of hyperparameter a.

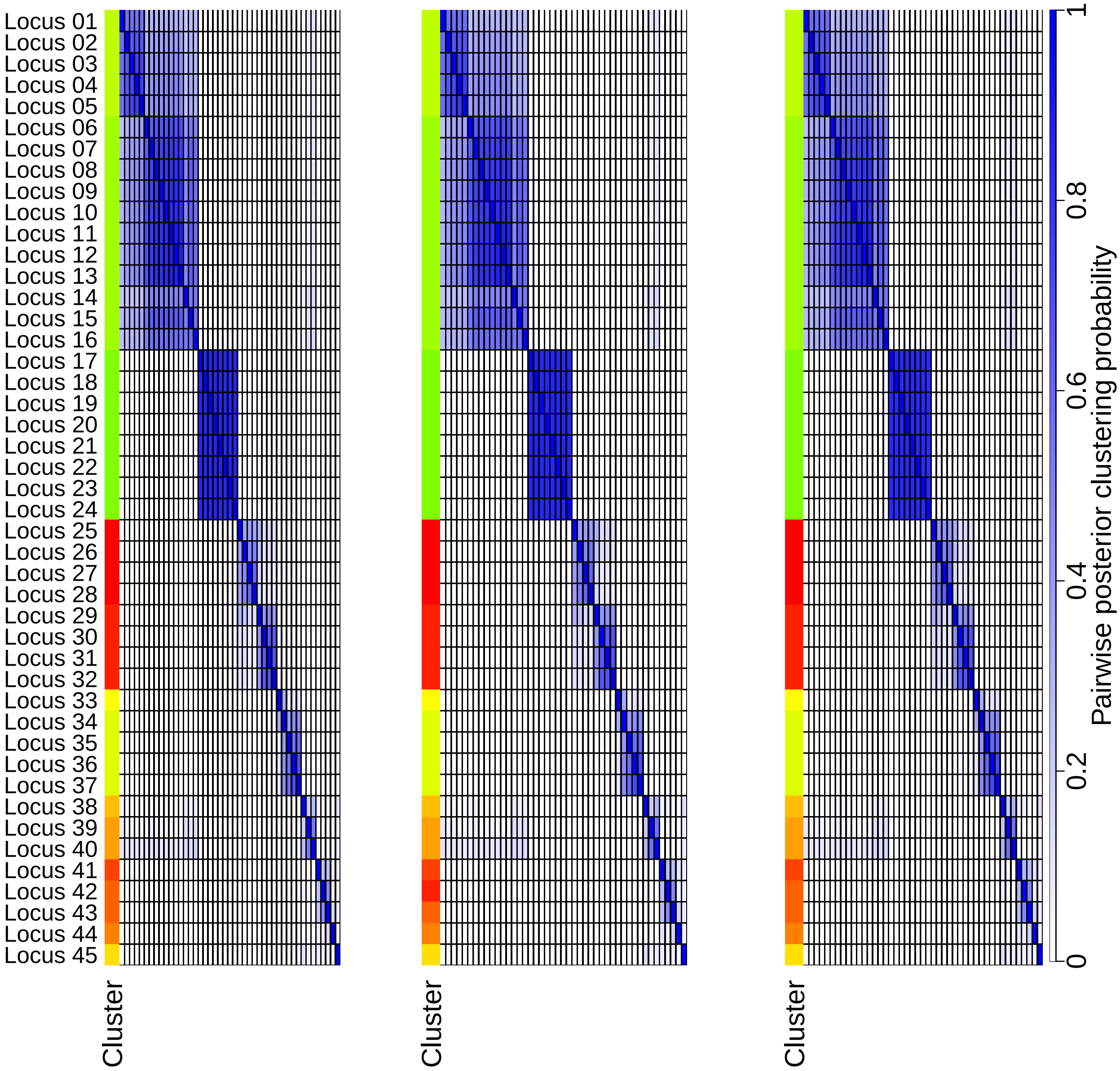

Figure S35: 3 independent MCMC chain, each ran for 100,000 iterations over the Xenograft sample SA501 timepoint X1.

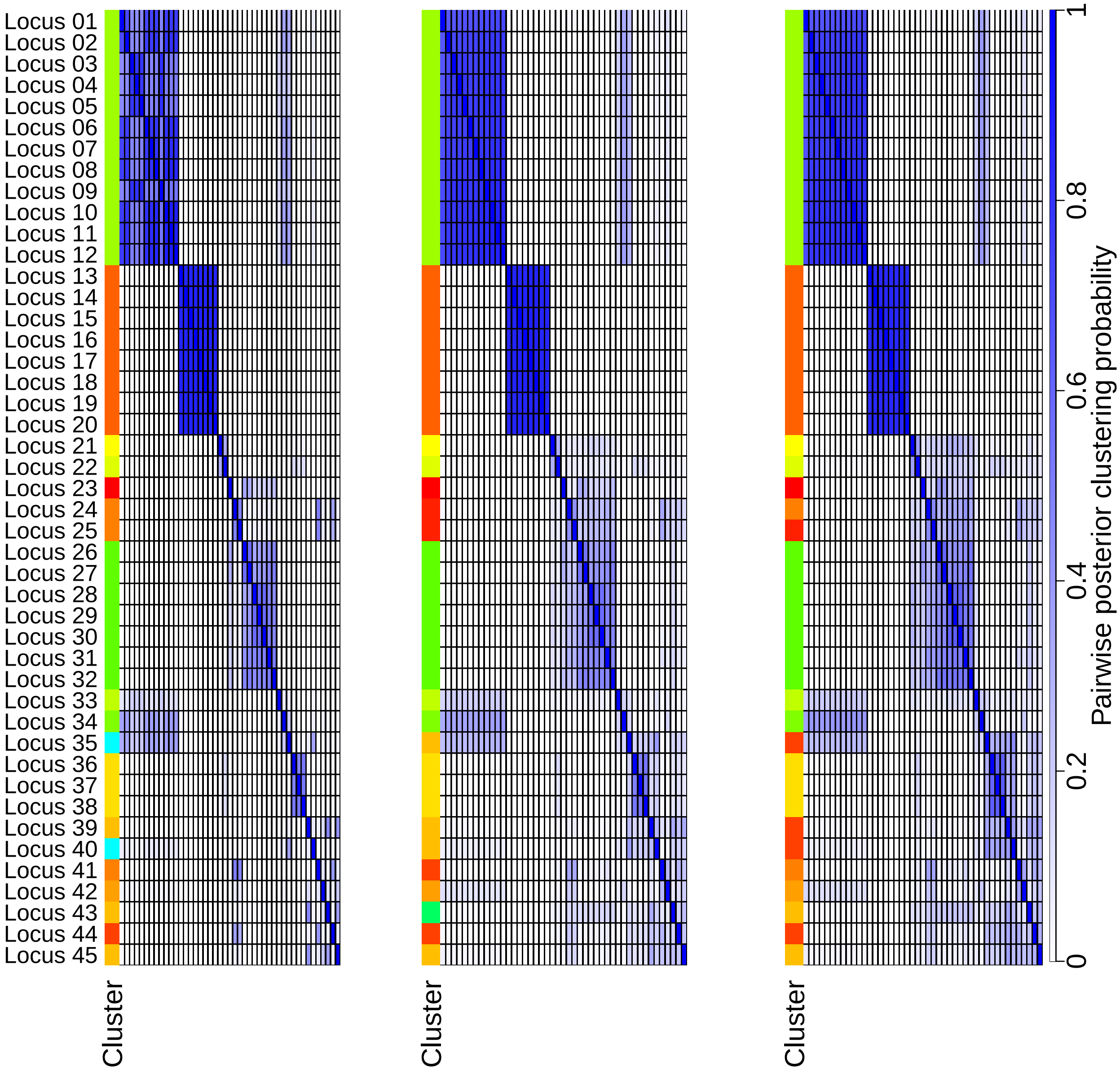

Figure S36: 3 independent MCMC chain, each ran for 100,000 iterations over the Xenograft sample SA501 timepoint X2.

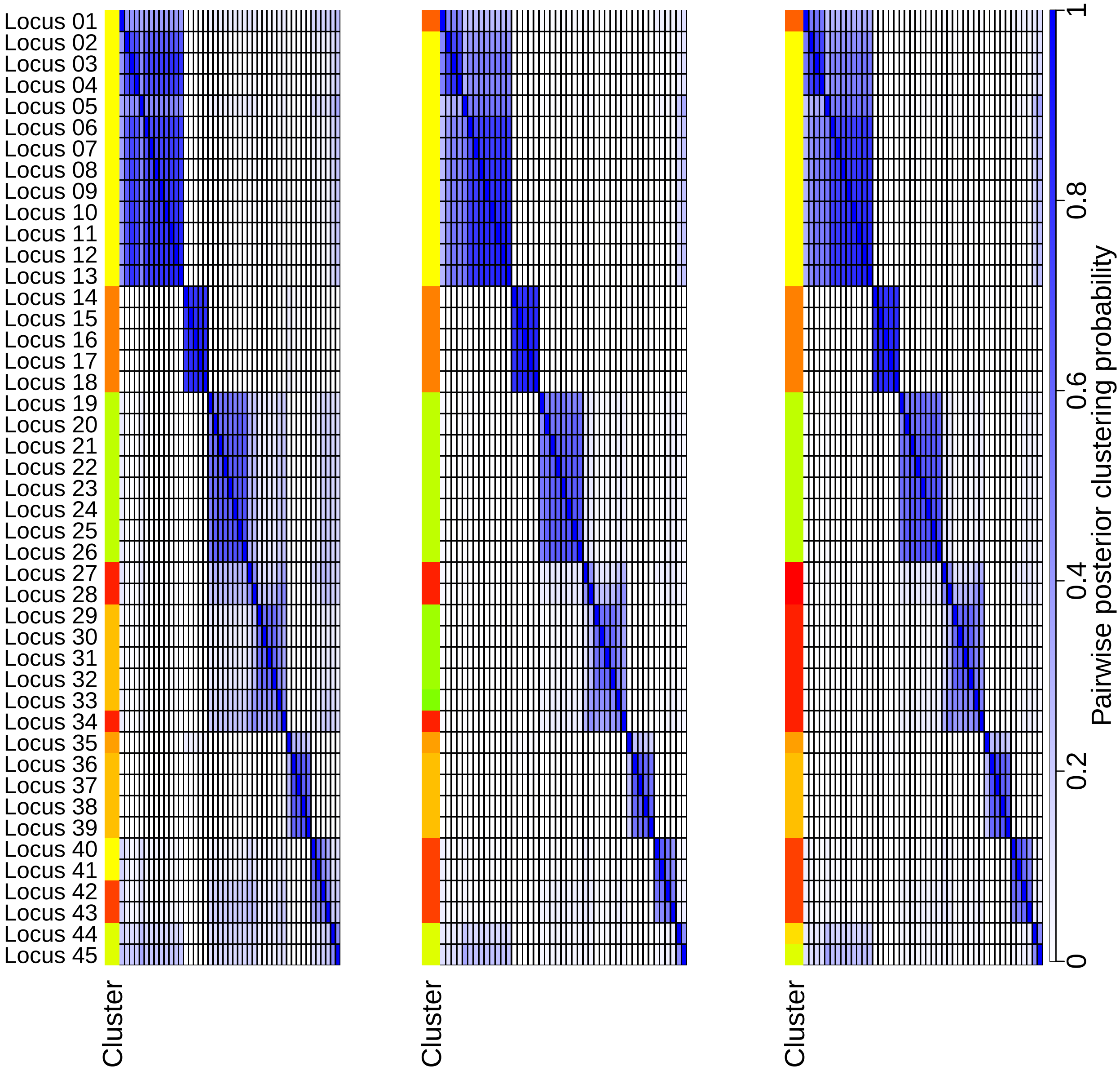

Figure S37: 3 independent MCMC chain, each ran for 100,000 iterations over the Xenograft sample SA501 timepoint X4.

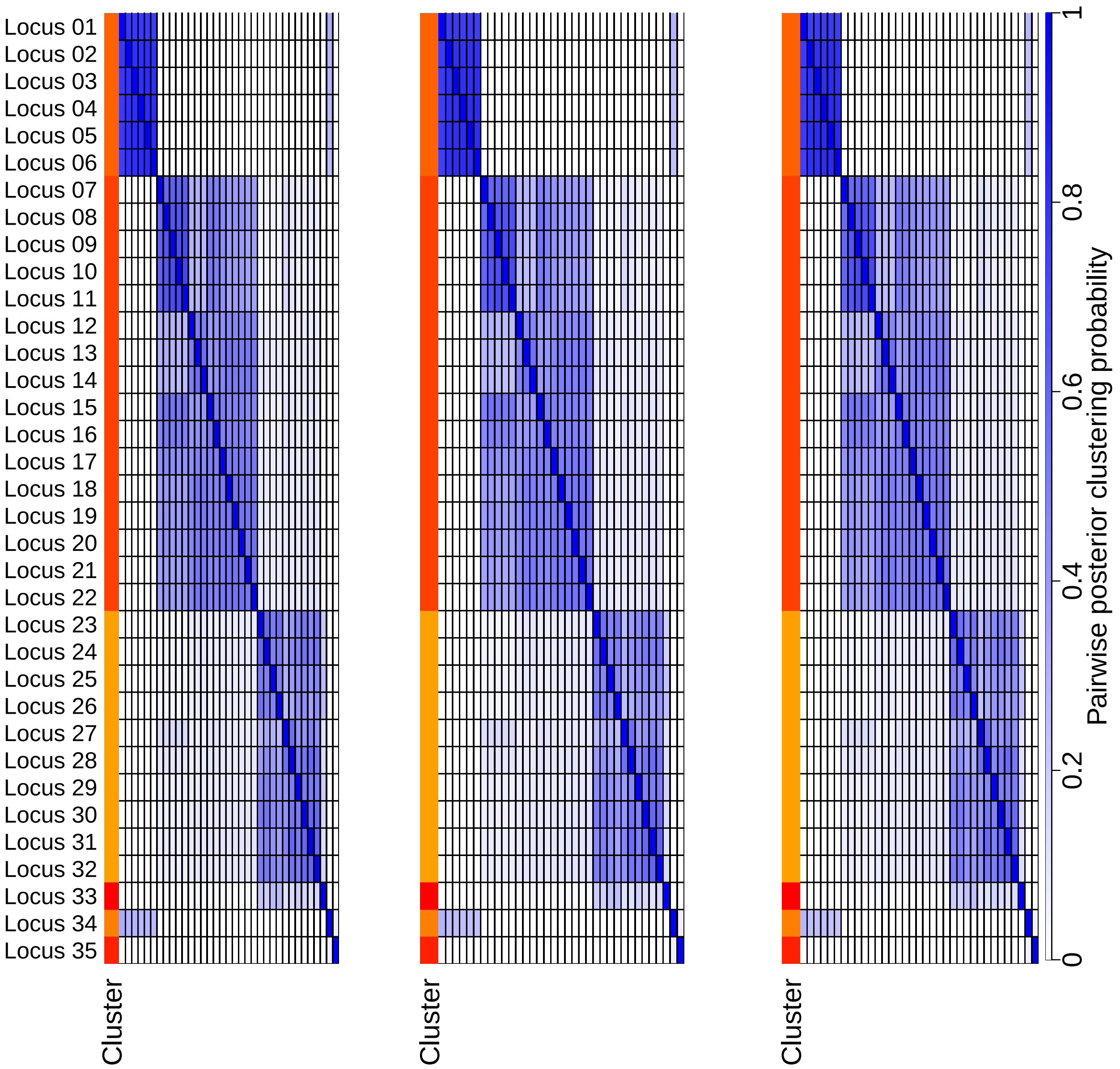

Figure S38: 3 independent MCMC chain, each ran for 100,000 iterations over the Xenograft sample SA494 timepoint T.

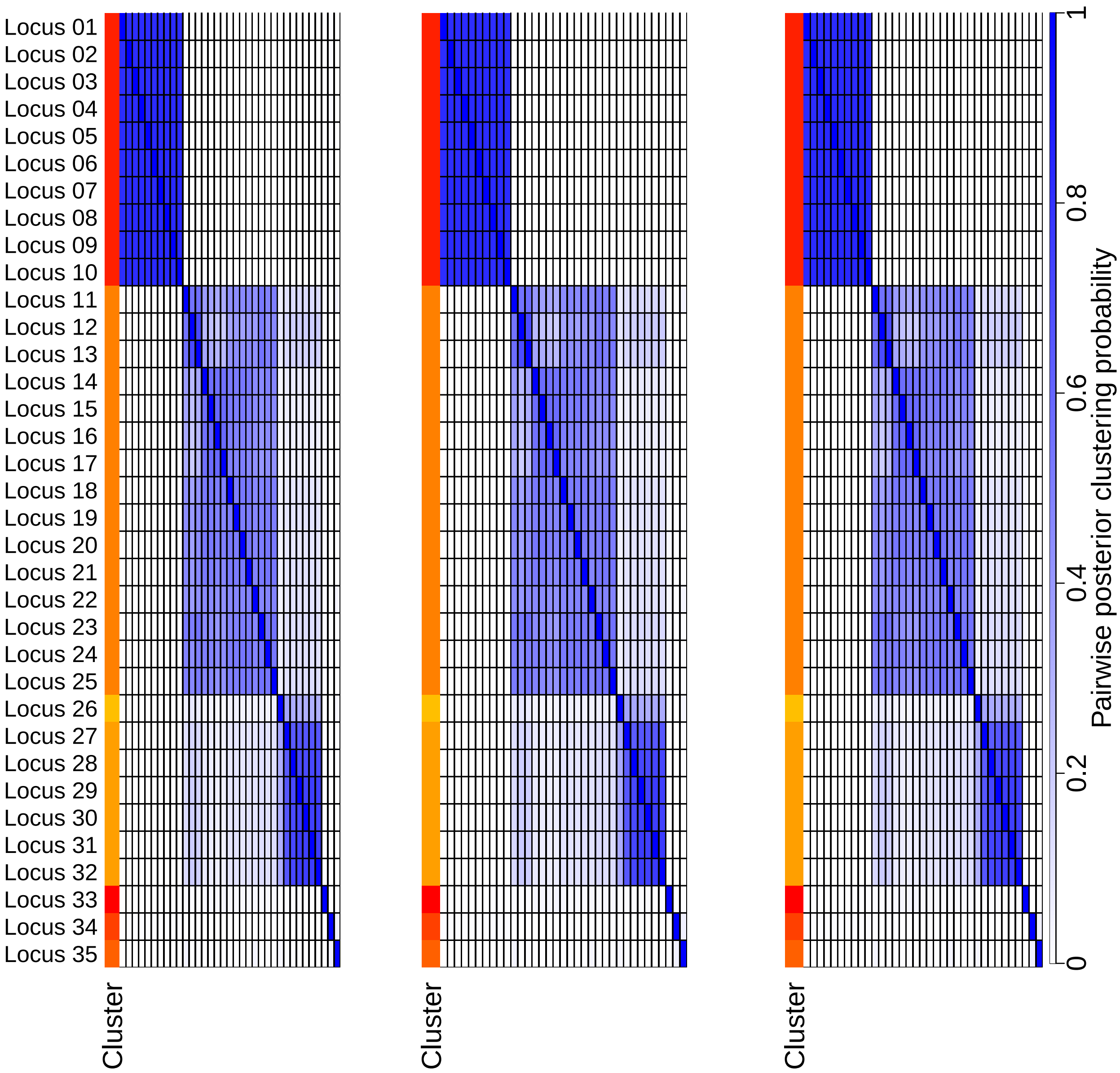

Figure S39: 3 independent MCMC chain, each ran for 100,000 iterations over the Xenograft sample SA494 timepoint X4.

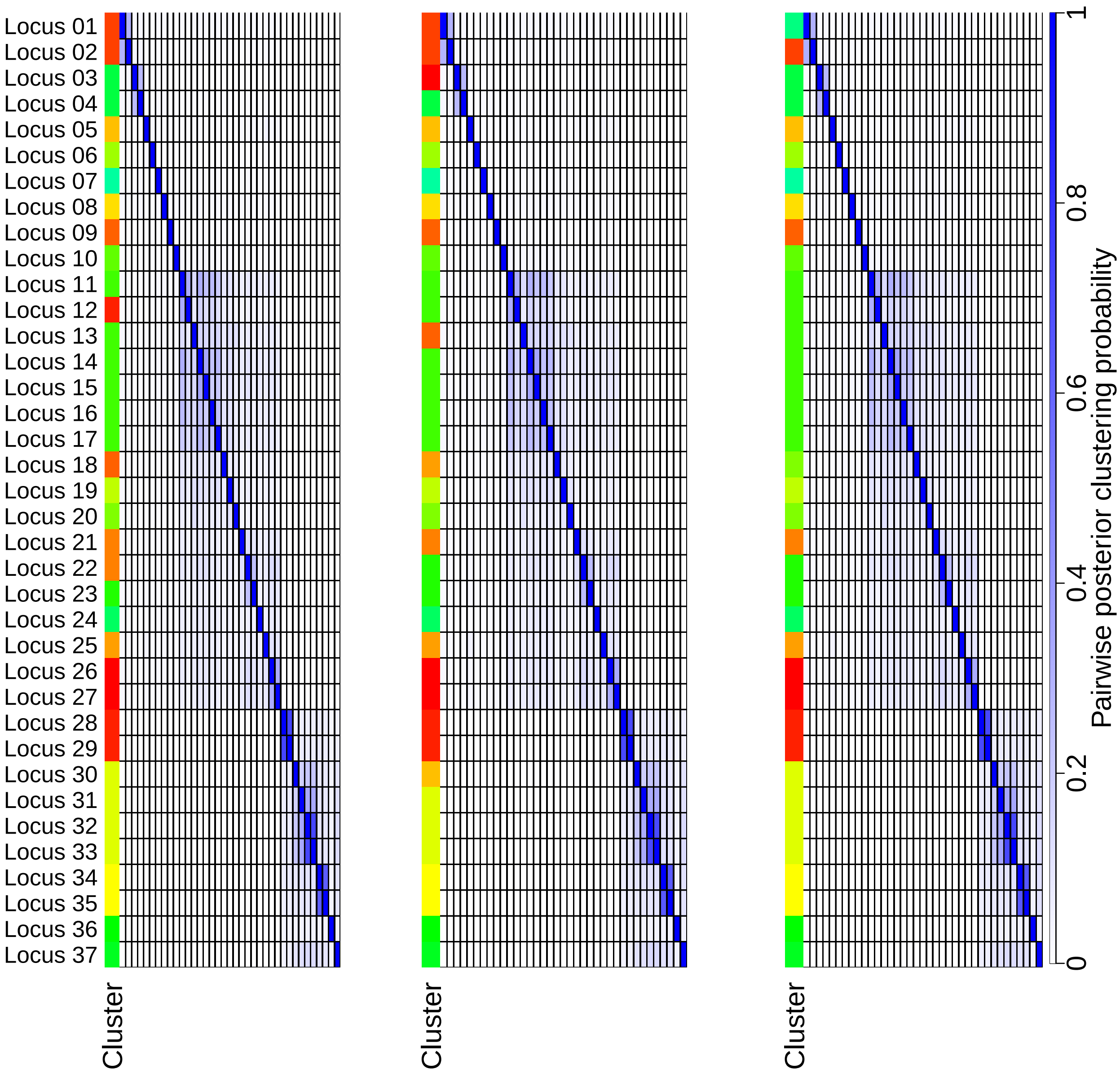

Figure S40: 3 independent MCMC chain, each ran for 100,000 iterations over the ITH Ovary patient P2 site omentum-site-1.

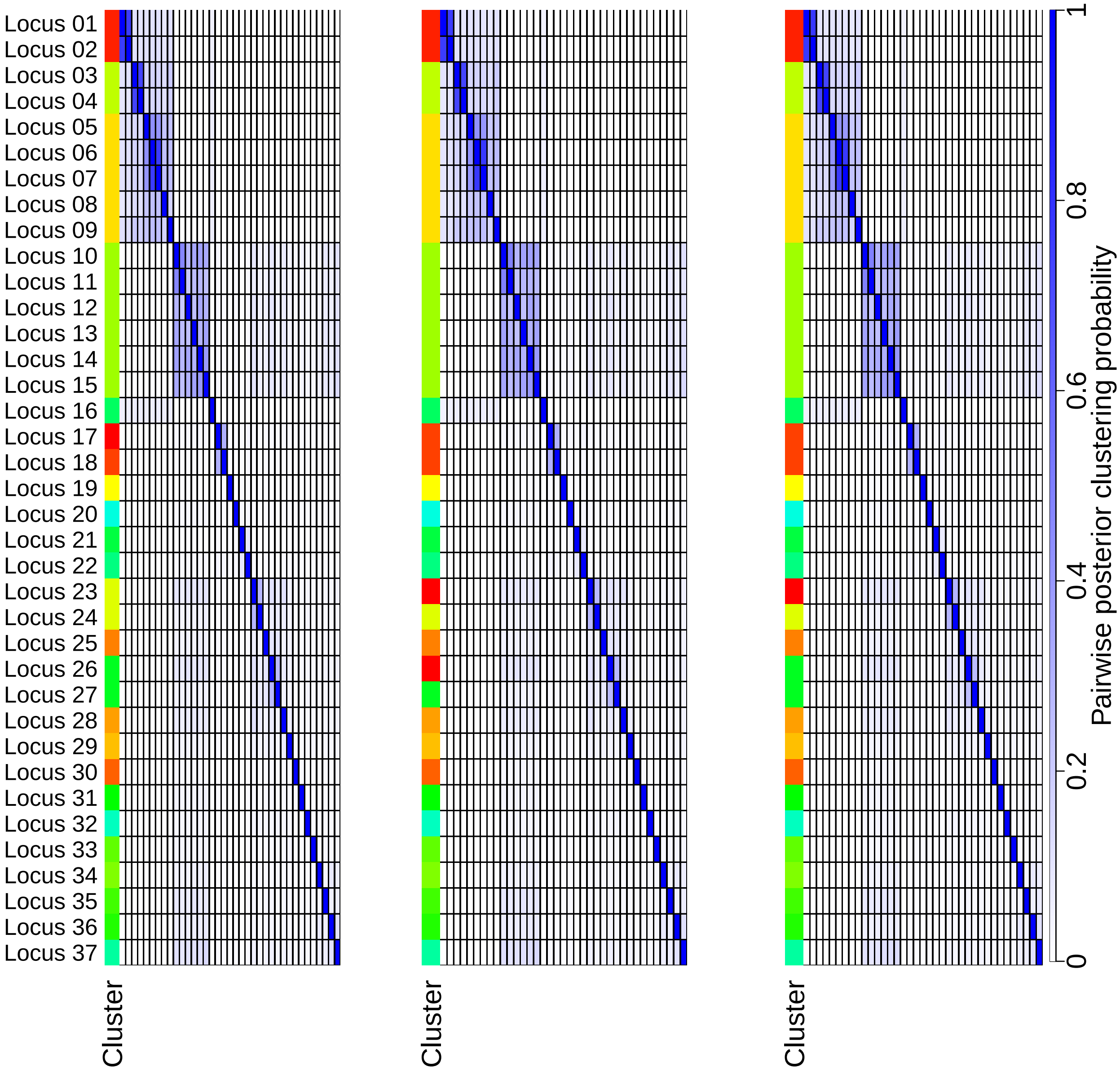

Figure S41: 3 independent MCMC chain, each ran for 100,000 iterations over the ITH Ovary patient P2 site omentum-site-2.

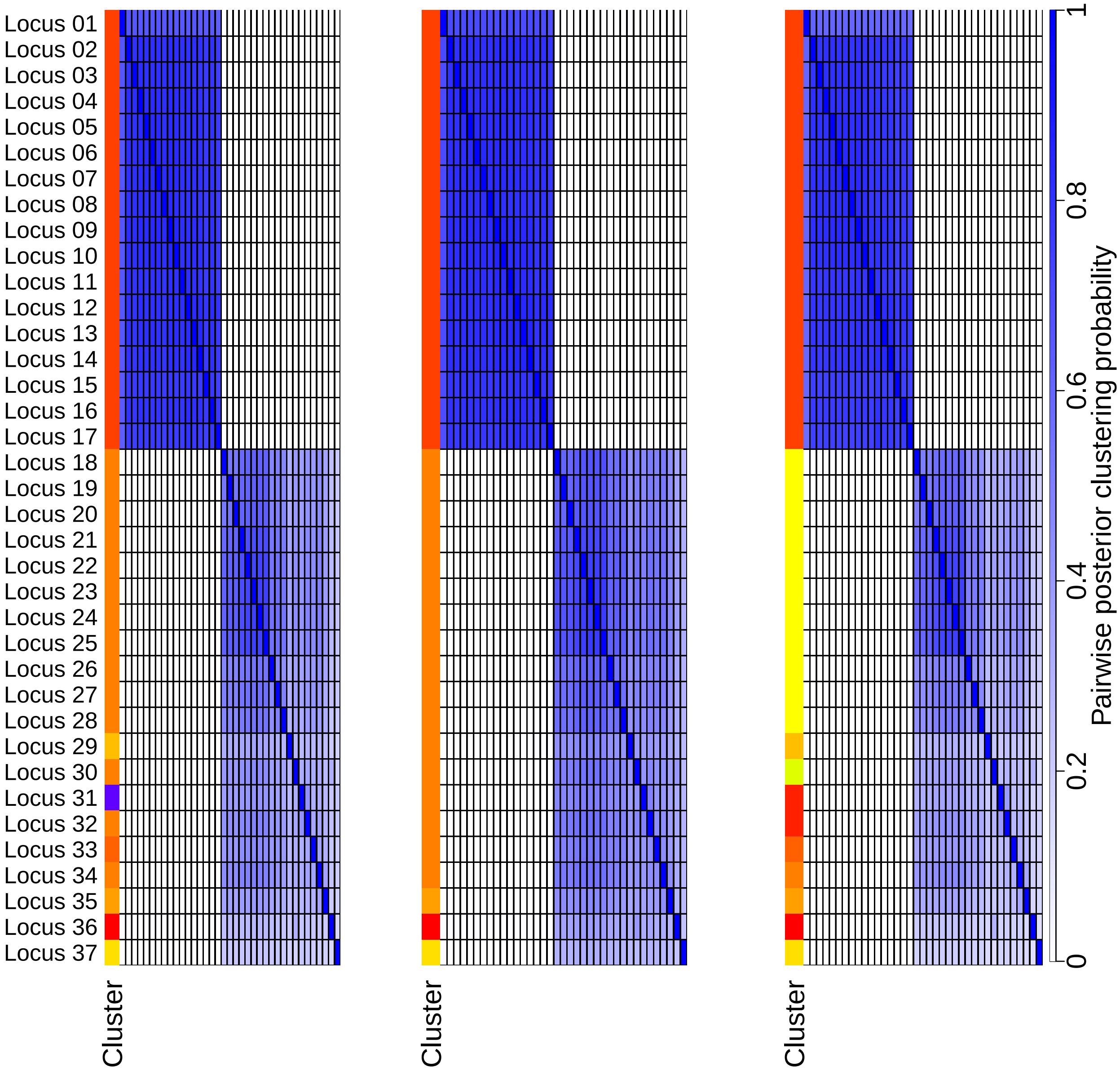

Figure S42: 3 independent MCMC chain, each ran for 100,000 iterations over the ITH Ovary patient P2 site right-ovary-site-1.

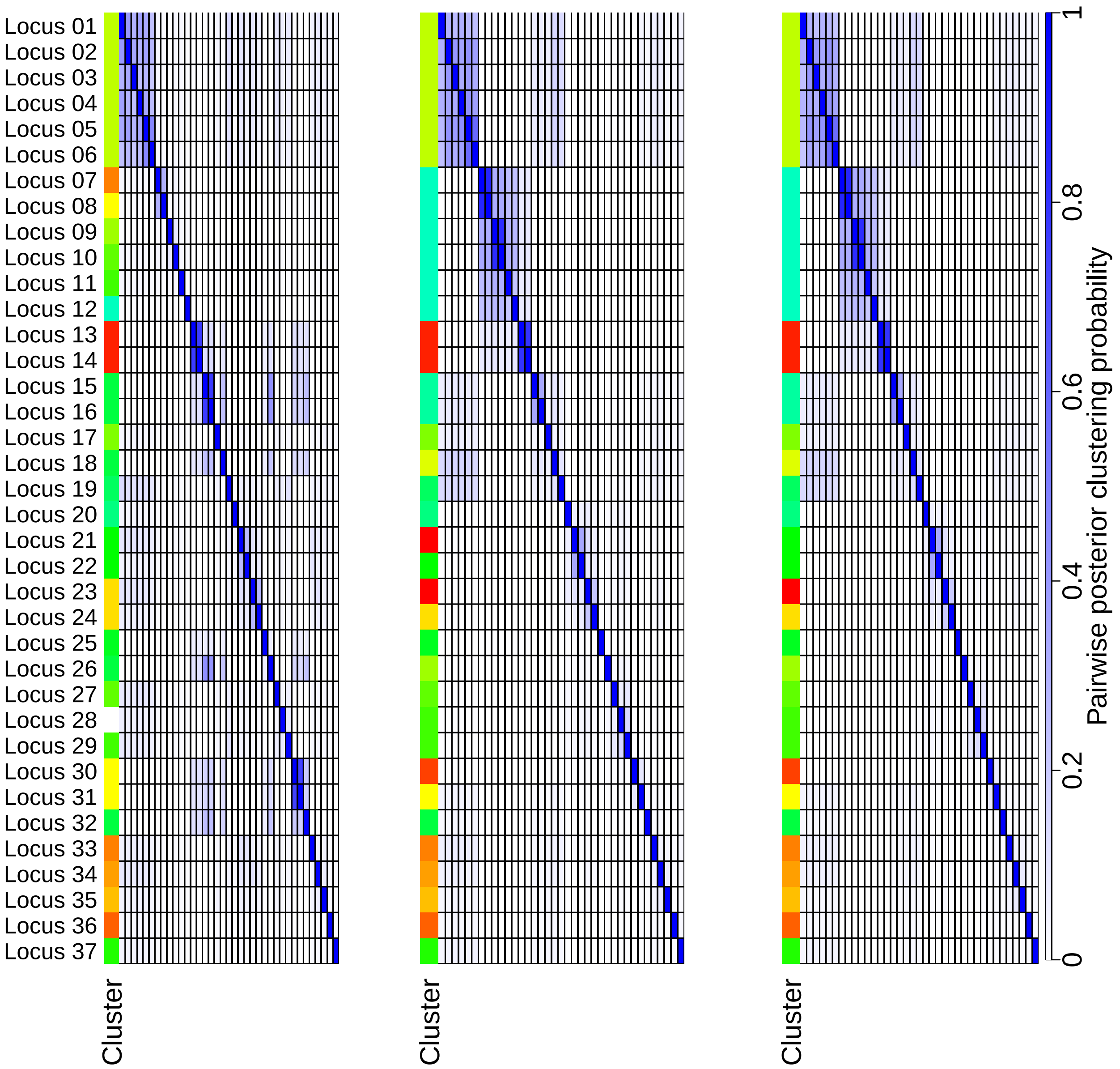

Figure S43: 3 independent MCMC chain, each ran for 100,000 iterations over the ITH Ovary patient P2 site right-ovary-site-2.

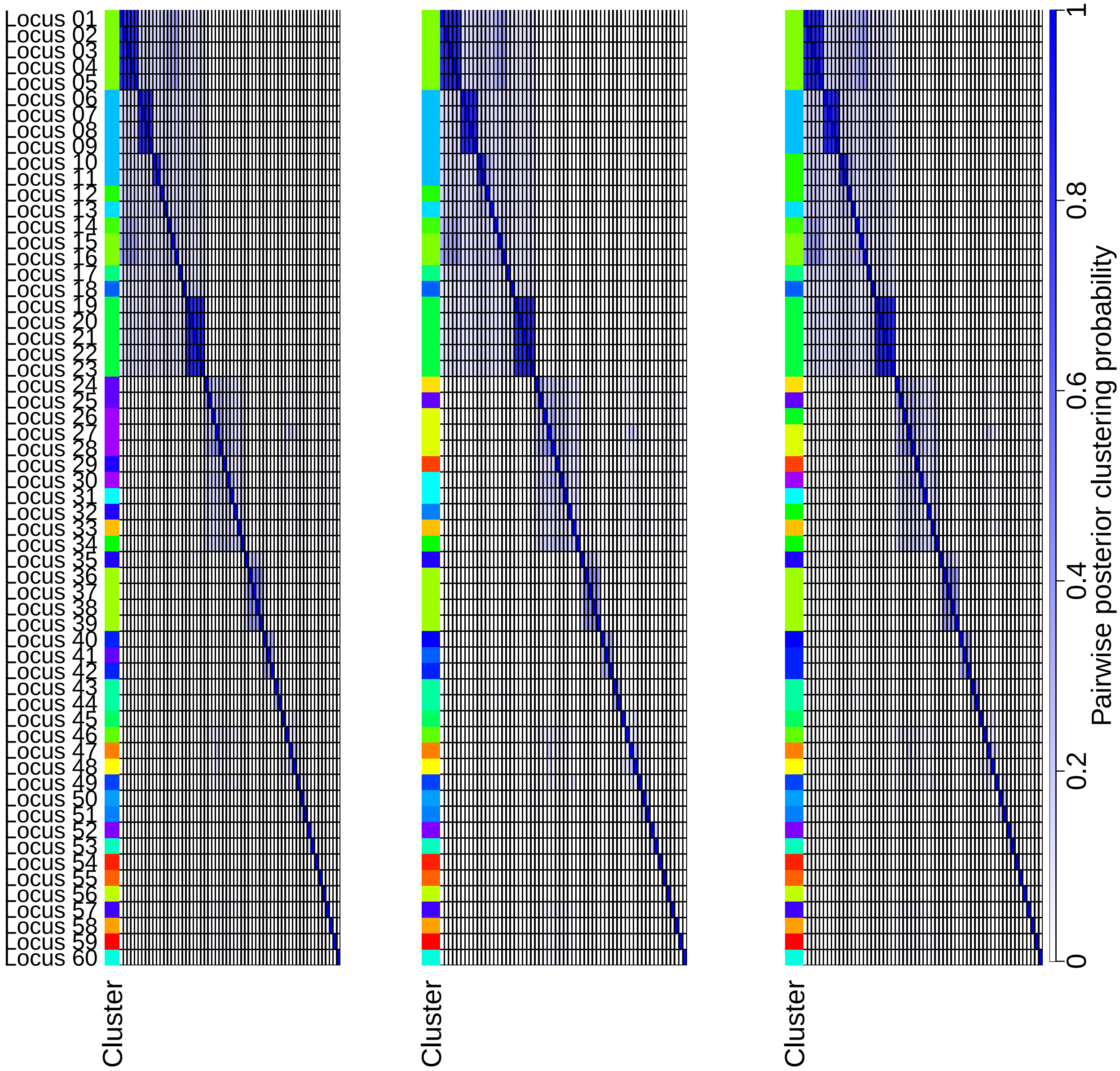

Figure S44: 3 independent MCMC chain, each ran for 100,000 iterations over the ITH Ovary patient P3 site adnexa-site-1.

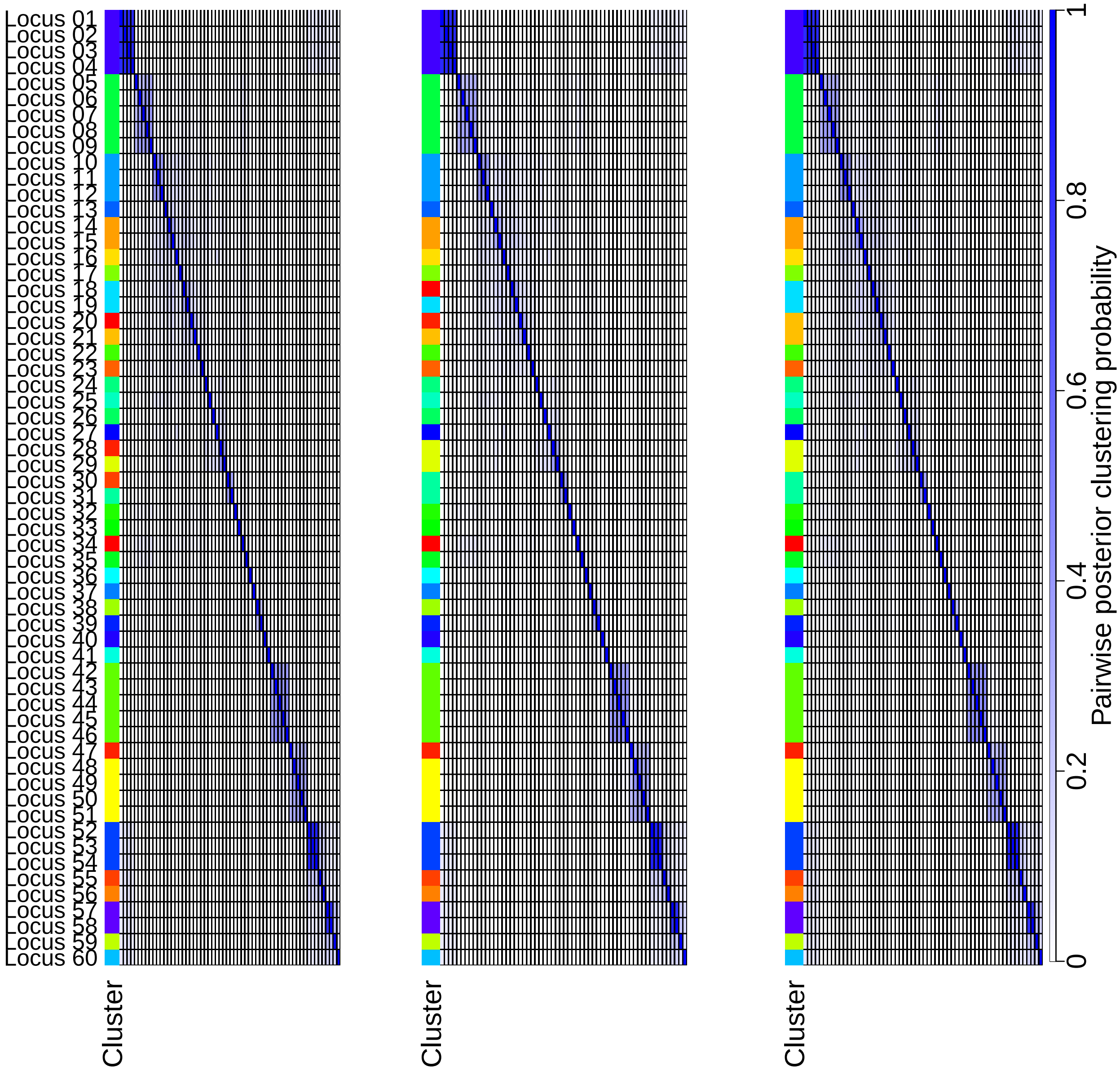

Figure S45: 3 independent MCMC chain, each ran for 100,000 iterations over the ITH Ovary patient P3 site omentum-site-1.

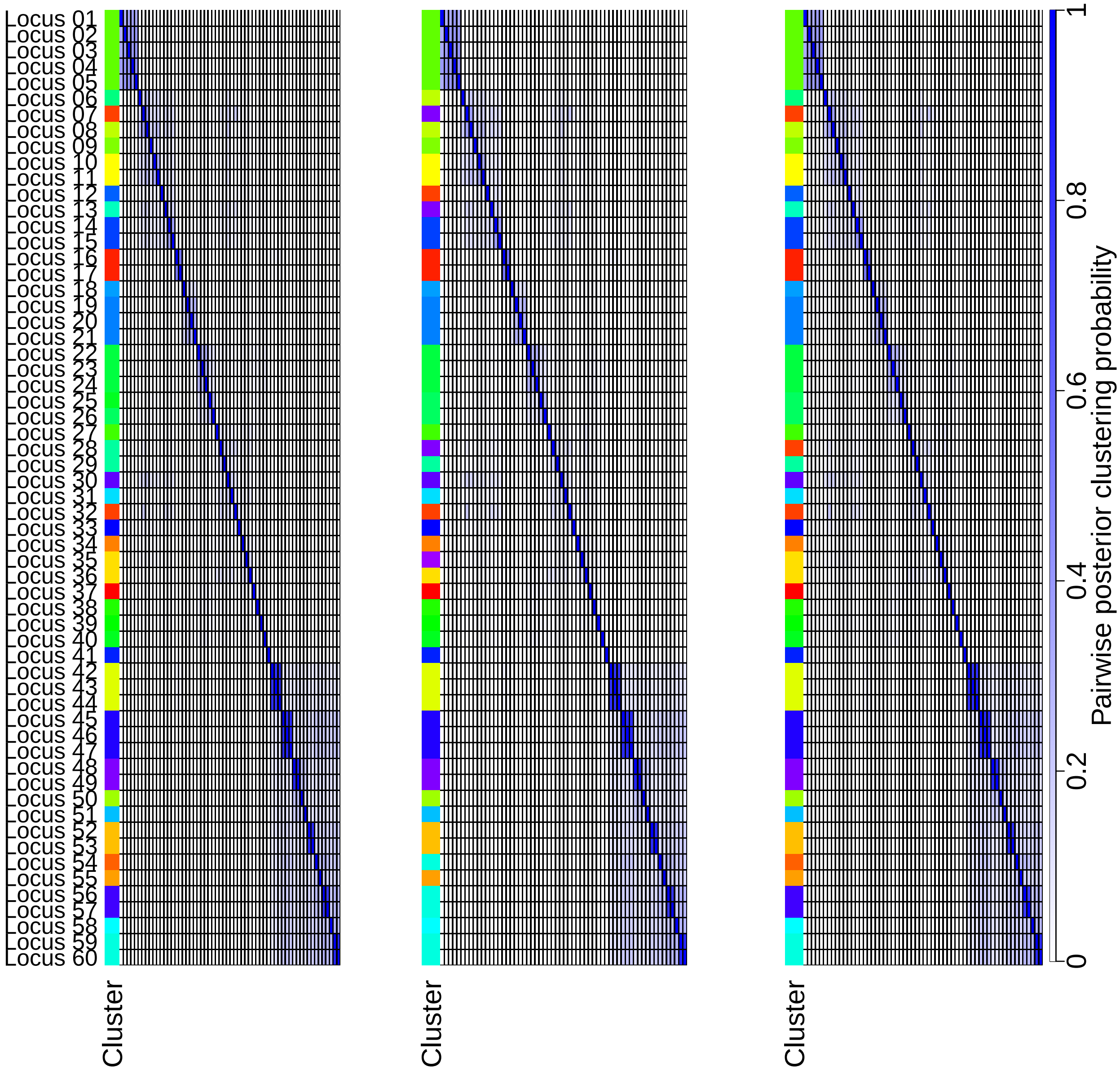

Figure S46: 3 independent MCMC chain, each ran for 100,000 iterations over the ITH Ovary patient P3 site right-ovary-site-1.

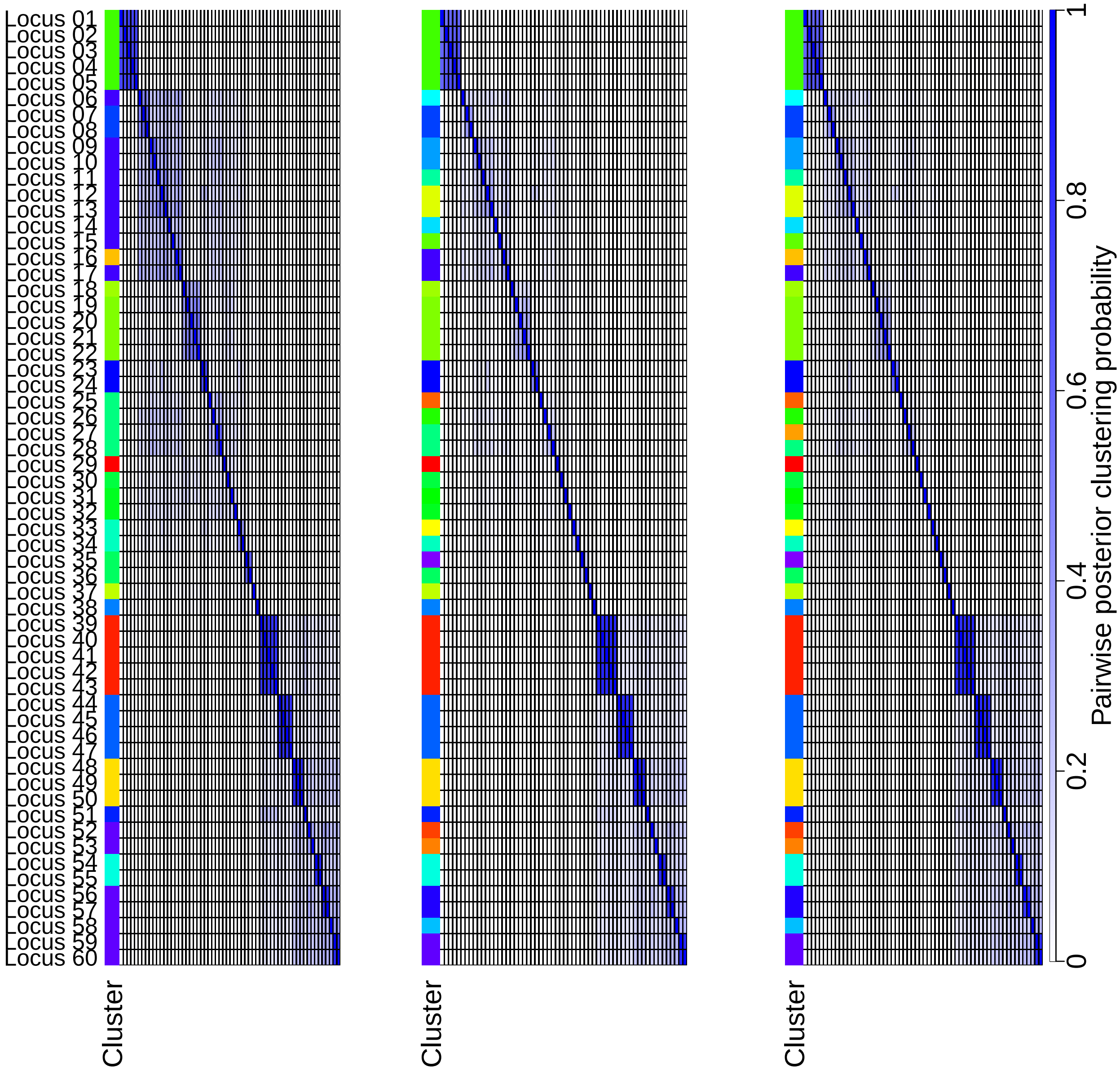

Figure S47: 3 independent MCMC chain, each ran for 100,000 iterations over the ITH Ovary patient P3 site right-ovary-site-2.

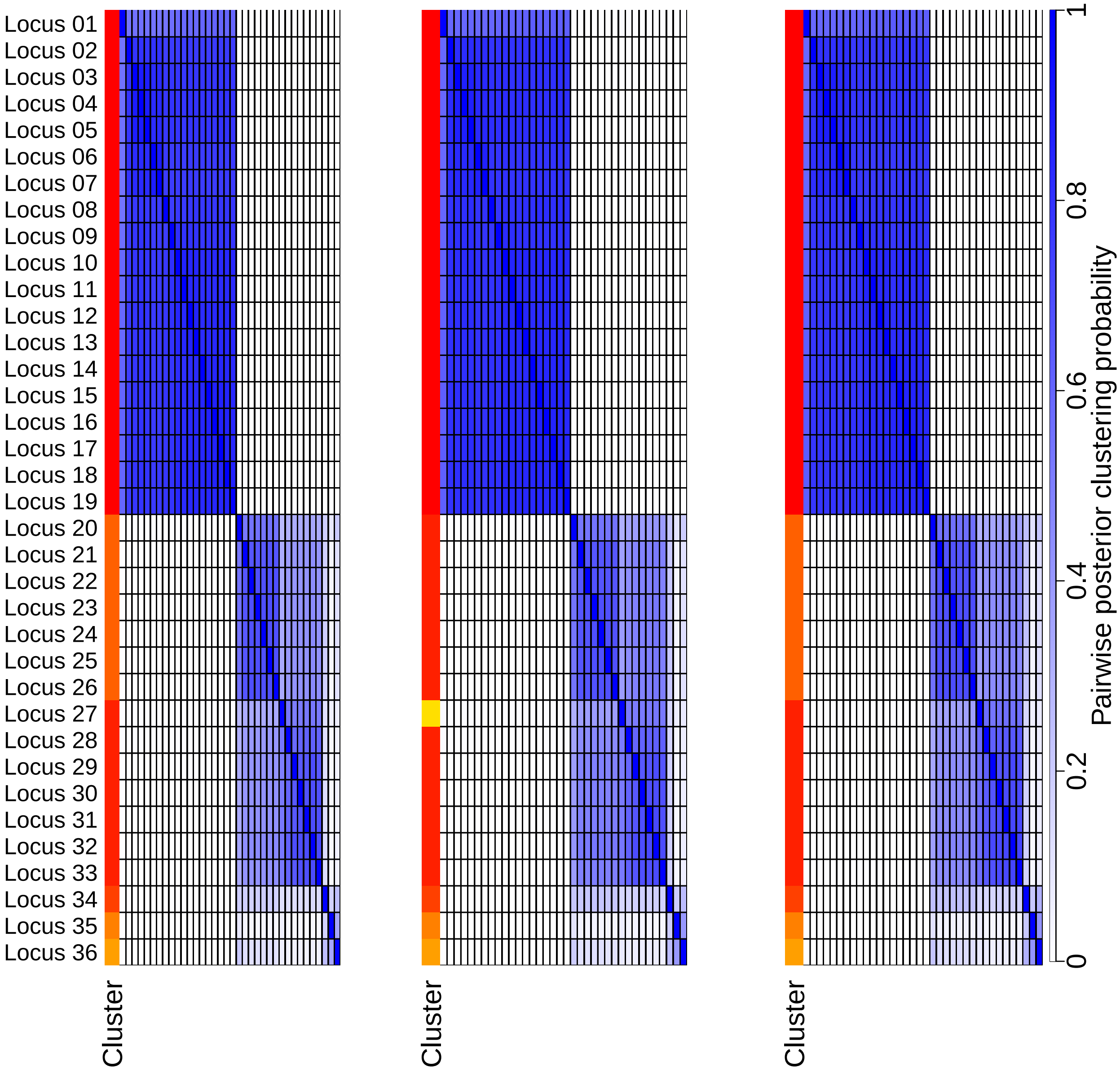

Figure S48: 3 independent MCMC chain, each ran for 100,000 iterations over the ITH Ovary patient P9 site left-ovary-site-1.

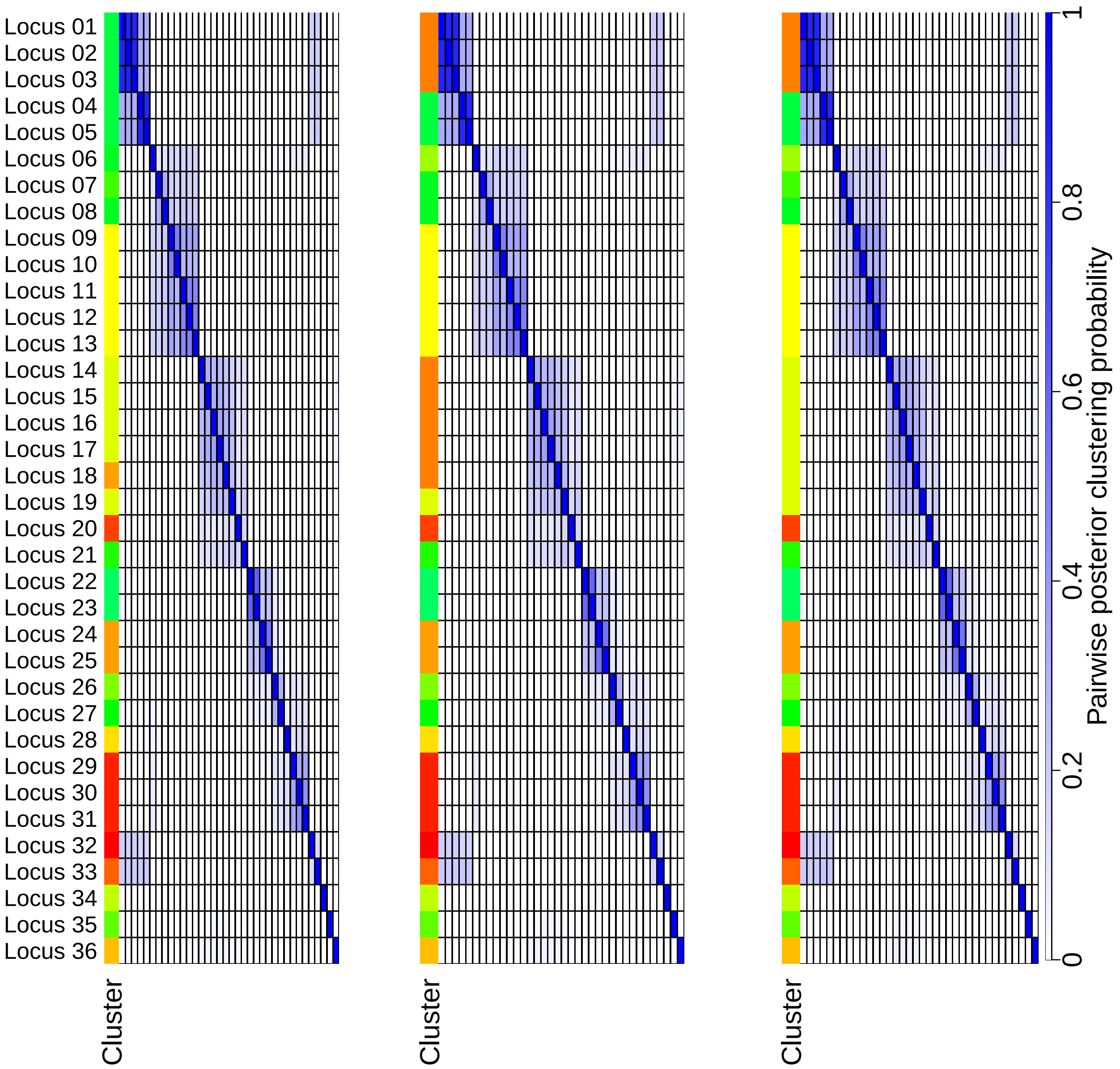

Figure S49: 3 independent MCMC chain, each ran for 100,000 iterations over the ITH Ovary patient P9 site left-ovary-site-2.

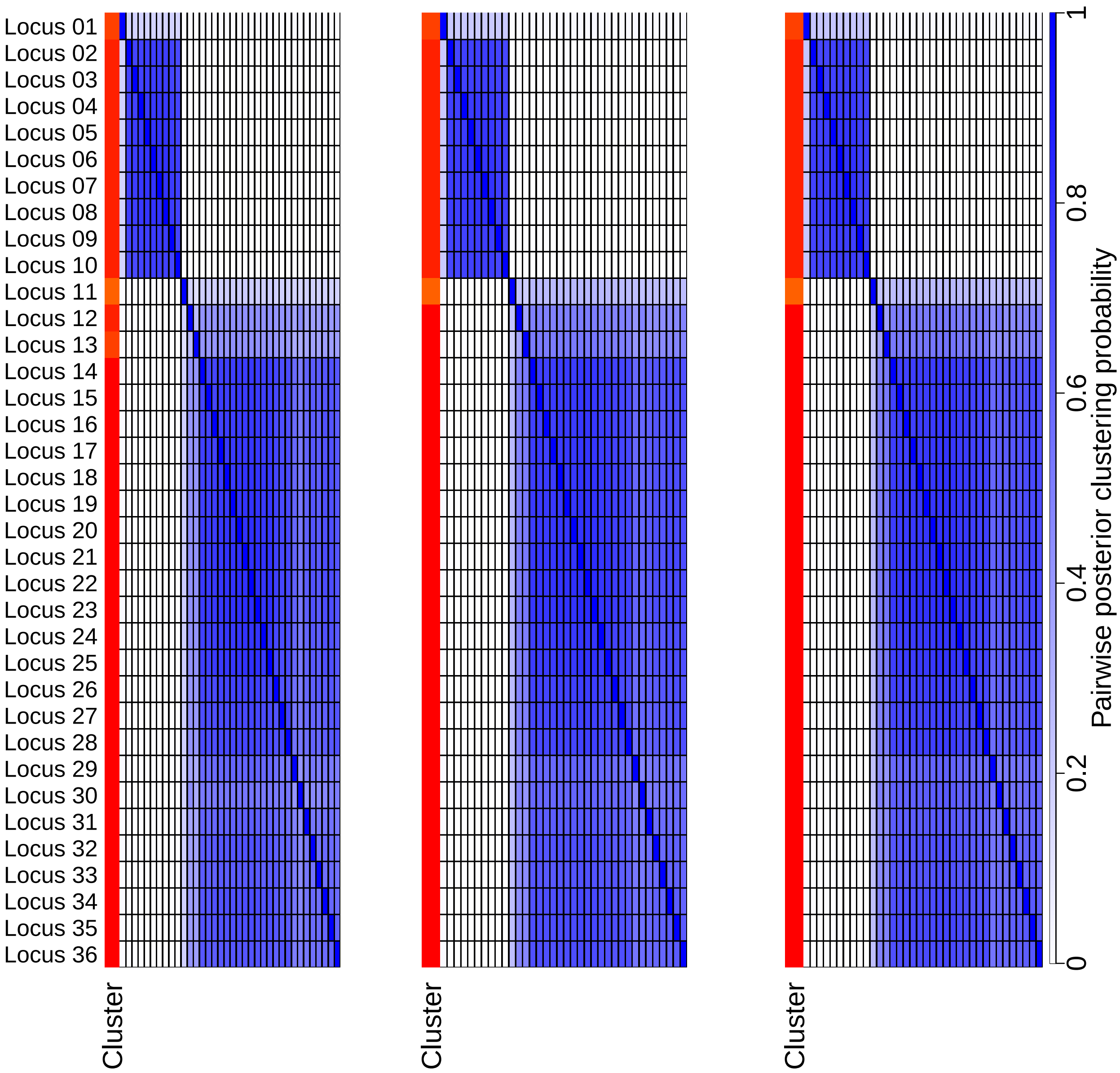

Figure S50: 3 independent MCMC chain, each ran for 100,000 iterations over the ITH Ovary patient P9 site omentum-site-1.

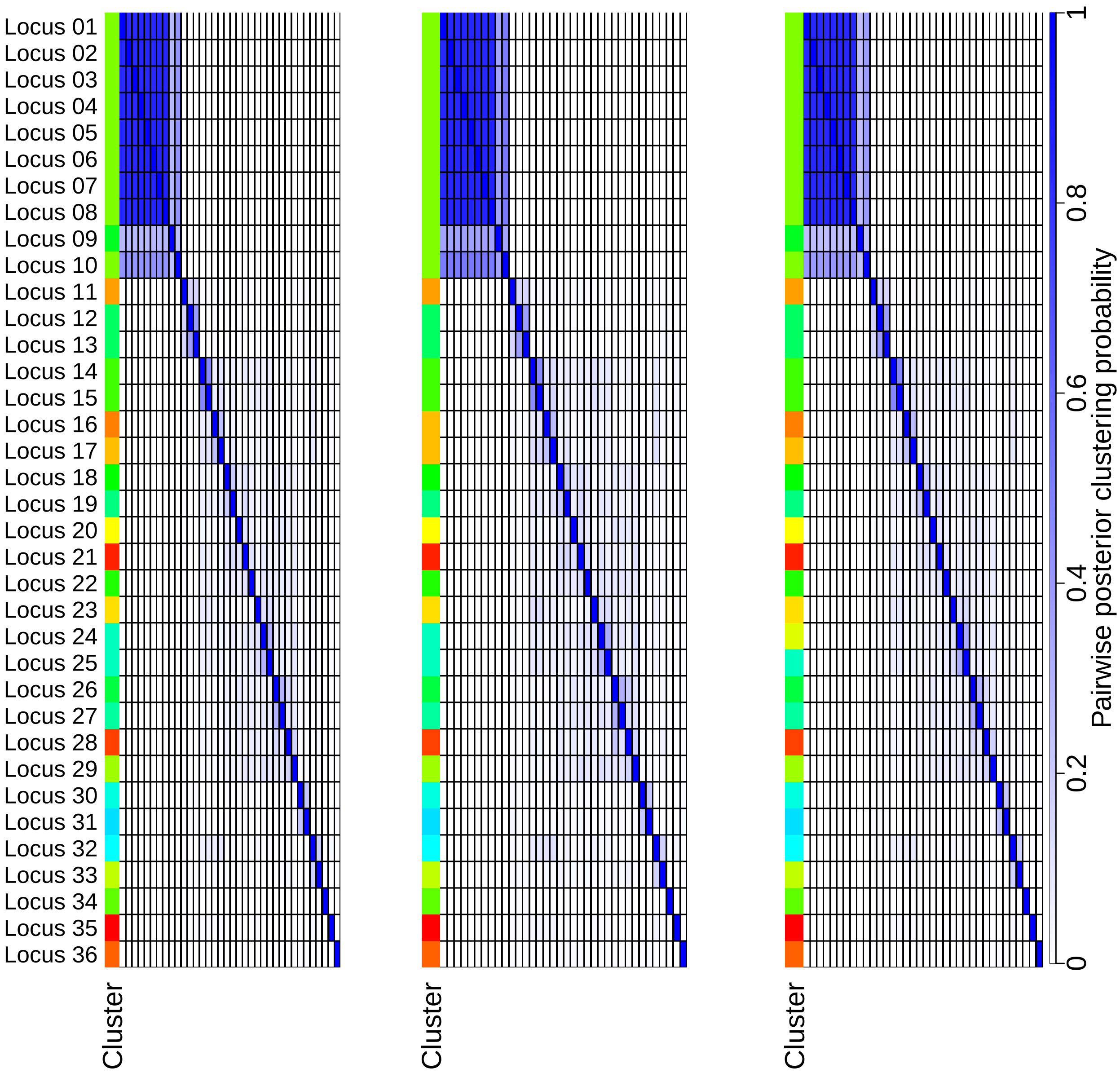

Figure S51: 3 independent MCMC chain, each ran for 100,000 iterations over the ITH Ovary patient P9 site omentum-site-2.

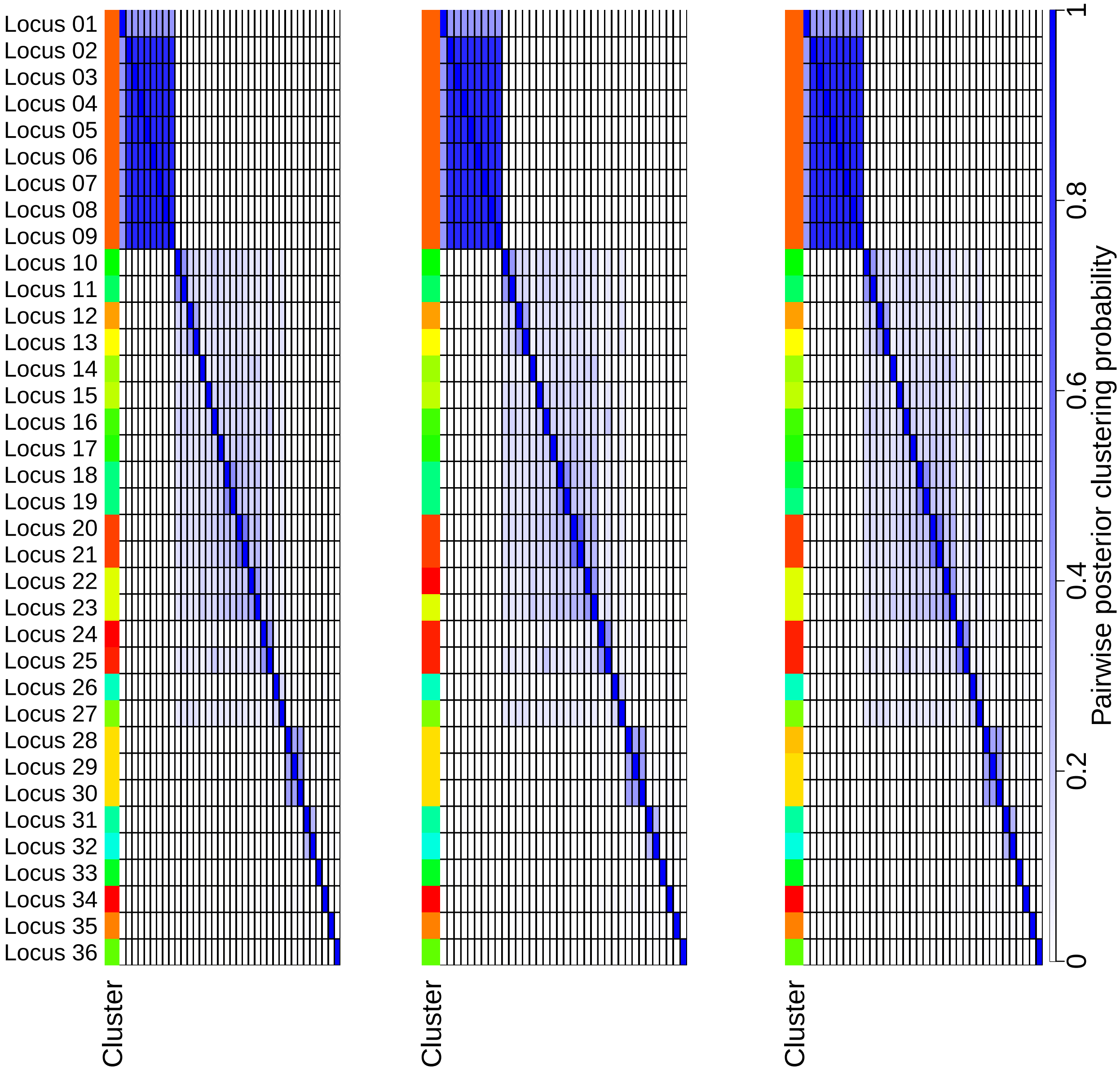

Figure S52: 3 independent MCMC chain, each ran for 100,000 iterations over the ITH Ovary patient P9 site right-ovary-site-1.

2 The ddClone model

Figure S3 illustrates our assumption about the relation between the genomic loci co-occurrence patterns and the underlying phylogenetic tree.

2.1 Simplified generative model

For exposition, we start with a simplified generative model in which we describe the relationship between inputs and the outputs of our method. Assume we have an heterogeneous tumour that contains subpopulations from two distinct haploid genotypes, g_1 and g_2 with clonal prevalences of 30% and 70%, respectively. For simplicity we set the tumour cellularity in our sample to one ($t = 1$). Since this implies that the expected fraction of variant allele reads is equal to cellular prevalence at each genomic locus ($\xi = \phi$), we will ignore ξ and directly use ϕ in this subsection.

The possible cellular prevalences for any genomic locus i in this tumour are $\phi_i = \{\phi_i^1 = 0.0, \phi_i^2 = 0.3, \phi_i^3 = 0.7, \phi_i^4 = 1.0\}$. This is because locus i is either not mutated in any of the genotypes (hence ϕ_1), only mutated in g_1 (corresponding to ϕ_2), only mutated in g_2 (therefore ϕ_3), or mutated in both g_1 and g_2 (meaning ϕ_4). These four cases represent our possible clusters.

To simulate the sequencing process for genomic locus i, we first pick its cluster. We use an auxiliary variable z_i as follows: $z_i \sim \text{Categorical}(w)$ where $w = w_{1:4}$ denotes the mixing weights, the proportion of clusters such that $\sum_{i=1}^{4} w_i = 1$.

The cellular prevalence for genomic locus i is now ϕ_{z_i}. **In the inference procedure, z_is and ϕ_{z_i}s constitute our desired outputs.** Next we simulate the number of variant alleles. Since according to our assumptions ϕ_{z_i} also denotes the expected proportion of variant reads in the sequencing experiment, we can relate it to the variant read counts b_i via a Binomial likelihood function as follows: $b_i \sim \text{Binom}(d_i, \phi_{z_i})$ where for now, we fix d_i, the total number of reads, to some appropriate constant value [1]. In the inference procedure, we observe b_i and d_i and they are the inputs to our model. Put together, we have:

$$\begin{aligned} z_i &\sim \text{Categorical}(w) \\ b_i &\sim \text{Binom}(d_i, \phi_{z_i}) \end{aligned} \tag{S1}$$

The two step process we described in Equation (S1) defines a mixture distribution as follows:

$$p(b_i) = \sum_{j=1}^{4} w_j \text{Binom}(d_i, \phi_j) \tag{S2}$$

[1] It could vary from about 10× in a whole genomic sequencing to about 10,000× in an ultra deep sequencing experiment [2].

Here we have four possible mixture components or clusters. Cluster assignments for each datapoint (a genomic locus in our model), are determined by the indicator variables z_i-s that are sampled from a categorical distribution, our prior over partitions, since we assumed that (i) the possible values for the ϕ_i-s were finite and (ii) known. Neither of these assumptions hold in general, that is, the ϕ_i-s could be any real-valued number in $[0, 1]$. To address this issue in a principled way, we introduce the Chinese Restaurant Process (CRP) in the main text.

Furthermore, co-occurrence patterns in g_1 and g_2 could be used to construct an informed prior over partitions of genomic loci. This can be done via a generalization of CRP, called ddCRP (Main text, Methods section).

Before describing our model, ddClone, in section 2, we present an updated generative process for the simplified example that we considered here in subsection 2.2.

2.2 Generative process for ddCRP mixture modelling

We now present the high level forward simulation algorithm for mixture modelling in ddCRP for the simplified example we considered in subsection 2.1:

1. For $i \in [1, N]$, draw $c_i \sim$ ddCRP(α, f, D).

   From this, derive $T(C)$, the corresponding table assignment.

2. For $i \in [1, K]$, draw $\phi_i \sim G_0$.

3. For $i \in [1, N]$, draw $b_i \sim F_i(\phi_{T_C(i)})$.

where α is a model parameter, f is a decay function, G_0 is the base distribution for the ϕ_i-s, F_i is the likelihood function relating expected number of reads to b_i to cellular prevalence ϕ_i as in Equation (S1), and $T_C(i)$ is the index of the table at which customer i is sitting.

Formally, in our simplified model, for each genomic locus $i \in [1, N]$, we want to infer ϕ_i and $T_C(i)$, given the model observations b_i and d_i. In section 2 we report a complete set of expected model inputs and outputs.

Here we introduce our model, ddClone. Figure S5 summarizes dependency and distributional assumptions in ddClone's model. Table S1 explains random variables used in this model.

Table S1: Notation reference for ddClone's probabilistic graphical model. [Notation reference for ddClone]

Variable	Description	Observed
A_0	Prior distribution over decay function's parameter a.	Yes
α_α	Shape hyperparameter over ddCRP distribution's α parameter.	Yes
β_α	Rate hyperparameter over ddCRP distribution's α parameter.	Yes
a	Decay function's parameter.	No
α	Model parameter for the ddCRP model.	No
H_0	Base measure for the ddCRP used to sample cellular prevalences for genomic loci.	Yes
α_s	Shape hyperparameter for the Beta-Binomial precision parameter s.	Yes
β_s	Rate hyperparameter for the Beta-Binomial precision parameter s.	Yes
D	Distance matrix over genomic loci. In this work, this is computed from single cell genotype analysis.	Yes
ddCRP	The distance dependent Chinese restaurant process with decay function f, distance matrix D, base measure H_0, and model parameter α.	No
s	Precision parameter for the Beta-Binomial emission model.	No
ϕ_i	Cellular prevalence for the genomic locus i.	No
d_i	Total number of reads that map to genome locus i.	Yes
b_i	Number of reads that map to variant allele at genomic locus i.	Yes
ψ_i	A vector (g_N^i, g_R^i, g_V^i) denoting genotype state at genomic locus i.	No
π_i	Prior over the genotype state for the genomic locus i.	Yes
t	Tumour cellularity.	Yes
N	Number of genomic loci.	Yes
M	Number of genotypes.	Yes

2.3 The Parental Copy Number (PCN) prior

Following [3], when copy number variation data in form of major and minor copy numbers is available, we have implemented a number of methods to elicit priors over locus genotypes. We assume that copy number

state at each genomic locus is reported as a pair of integers $(\bar{\zeta}_1, \bar{\zeta}_2)$, where the major copy number $\bar{\zeta}_1$, refers to the maximum of the two said integers and the minor copy number $\bar{\zeta}_2$ refers to the minimum of the pair and $\bar{\zeta} = \bar{\zeta}_1 + \bar{\zeta}_2$ is the total copy number. Here we describe the Parental Copy Number (PCN) strategy that is used in our experiments.

Let $\mathcal{P}$ denote the set of locus genotype states that PCN scheme describes. We assign equal weight to the locus genotype states in $\mathcal{P}$ and zero weight to any other locus genotype state that is not a member of $\mathcal{P}$, that is a locus genotype state $\psi_i \in \mathcal{P}$ has a weight equal to $\frac{1}{|\mathcal{P}|}$. The locus genotype states with non-zero weights are $\mathcal{P} = \{\psi_1, \psi_2, \psi_3, \psi_4\}$ where

- $\psi_1 = (g_N = (2, 0), g_R = (2, 0), g_V = (\bar{\zeta}_1, \bar{\zeta}_2))$
- $\psi_2 = (g_N = (2, 0), g_R = (2, 0), g_V = (\bar{\zeta}_2, \bar{\zeta}_1))$
- $\psi_3 = (g_N = (2, 0), g_R = (2, 0), g_V = (\bar{\zeta} - 1, 1))$
- $\psi_4 = (g_N = (2, 0), g_R = (\bar{\zeta}, 0), g_V = (\bar{\zeta} - 1, 1))$

These locus genotype states adhere to the following conditions: $g_N = (2, 0)$ so that the normal locus genotype is diploid with respect to the reference alleles, and $\zeta(g_V) = \bar{\zeta}$ and $b(g_V) \in \{1, \bar{\zeta}_1, \bar{\zeta}_2\}$. The number of variant alleles $b(g_V)$ is at least one, in other words we do not consider genomic loci that are not mutated. When $b(g_V) \in \{\bar{\zeta}_1, \bar{\zeta}_2\}$, we set $g_R = g_N$ (as in ψ_1, ψ_2). For $b(g_V) = 1$, we consider two scenarios: (i) either the point mutation event has happened before the copy number event, in which case we set $g_R = g_N$ (see ψ_3), or (ii) the copy number event preceded the point mutation, where we choose g_R such that $\zeta(g_R) = \bar{\zeta}$ and $b(g_R) = 0$ (as in ψ_4).

We note that for some copy number configurations such as when $\bar{\zeta}_1 = \bar{\zeta}_2$ or $\bar{\zeta}_2 = 0$, some ψ_i values will be identical. For example, when total copy number is equal to one, the possible locus genotype states in the PCN scheme are $\mathcal{P} = \{\psi_1 = (g_N = (2, 0), g_R = (2, 0), g_V = (0, 1)), \psi_2 = (g_N = (2, 0), g_R = (1, 0), g_V = (0, 1))\}$.

2.4 Resampling hyperparameters

α and a are resampled using methods described in [4]. Briefly, we used the following Gibbs move to update the value of hyperparameter α given the customer connection configuration C:

$$p(\alpha|C) \propto \alpha^K [\prod_{i=1}^{N} (\alpha + \sum_{j \neq i} f(d_{ij}))]^{-1} p(\alpha) \quad \text{(S3)}$$

where $K = \sum_i^N c_i = i$, i.e., the number of self-connections, and $p(\alpha) \sim \text{Gamma}(\alpha_0, \beta_0)$ is the Gamma prior over α with shape and rate parameters α_0 and β_0.

The decay function parameter a is updated using the following Gibbs move:

$$p(a|C,\alpha) \propto [\prod_{i:c_i \neq i} f(d_{ij}, a)][\prod_{i=1}^{N} (\alpha + \sum_{j=1}^{i-1} f(d_{ij}, a))]^{-1} p(a|\alpha) \quad \text{(S4)}$$

where we assume a uniform prior on a independent of α.

Since the decay function is exponential in our model, we use the Griddy-Gibbs [5] approach to sample approximately from Equation (S4).

We use the method proposed in [6] for resampling s.

Gamma distributed priors are characterized using shape α and rate β parameters. Equation S5 shows the corresponding distribution function:

$$g(x; \alpha, \beta) = \frac{\beta^{\alpha}, x^{\alpha-1} e^{-x\beta}}{\Gamma(\alpha)} \quad \text{(S5)}$$

where $\Gamma(\alpha)$ is the Gamma function.

By default, hyperparameter resampling is enabled in our experiments in this work, unless otherwise specified. We note that to explore the model's sensitivity to the value of hyperparameters, in some of our experiments in section 6.2, we disable hyperparameter resampling. We specify this in the description of those experiments.

This Gibbs sampler potentially displaces more customers at each step, and as such might have better mixing properties compared to the traditional CRP Gibbs sampler [4]. Figure S6 shows such a step in

ddCRP.

2.5 The modified Jaccard distance

The Jaccard index is symmetric with regard to the FP and FN noises, thus we may use a modified Jaccard distance that takes into account the higher FN rate (with respect to FP rate) and assigns the expected value of the distance given an estimated FN rate. Intuitively, it constitutes a softer prior over co-occurrence patterns in the single cell data. Let's assume that compared to the FN rate, the FP rate is negligible. The issue with the Jaccard distance (JD) is that it ignores the possibility that an observed value of 0 in the genotype matrix could actually be 1 with probability p_{FN} and for each locus observed at genotype x and y it assigns the following values (to be normalized):

Table S2: The value-table for the traditional Jaccard distance

x	y	Jaccard dist
0	0	0
0	1	1
1	0	1
1	1	0

We incorporate the uncertainty imposed by p_{FN} by computing the expected value of the Jaccard distance conditioned on the observations. It is straight forward to show that the modified Jaccard distance (MJD) at each locus, for two genotypes, would be:

Table S3: The value-table for the modified Jaccard distance

x	y	Modified Jaccard dist
0	0	$2p(1-p)$
0	1	$1-p$
1	0	$1-p$
1	1	0

where p is shorthand for p_{FP}.

For two binary vectors X and Y of size m, we can define the Jaccard distance as follows:

$$\frac{|X| + |Y| - 2|X \cap Y|}{|X| + |Y| - |X \cap Y|} \tag{S6}$$

where $|.|$ is the set size. In our case where X and Y are binary vectors, $|.|$ equals the number of ones in

the vector and $|X \cap Y|$ is the number of indexes for which both X and Y are one. Now using the value-table of the MJD, we define a probabilistic counterpart for the quantities in Equation S6. Let $|.|_p$ be the expected number of ones in its input binary vector, then:

$$|X|_p = \text{number of observed ones in X} + p \times (\text{number of observed zeros in X}) \tag{S7}$$

Thus MJD for binary vectors X and Y and FN rate p is defined as:

$$MJD(X, Y; p) := \frac{|X|_p + |Y|_p - 2|X \cap Y|_p}{|X|_p + |Y|_p - |X \cap Y|_p} \tag{S8}$$

When $p = 0$, we recover the traditional Jaccard distance. This formulation accounts for loci that are co-unobserved and less harshly penalizes mis-observed pairs (occurrences where for two loci, one is observed in genotype and the other is not) according to the estimated FN rate.

We assume in the following that FN rate equals the ADO rate. We ran ddClone with this modified Jaccard distance (MJD) over 10 simulated datasets with ADO rate of 0.3. As demonstrated in Figure S7, using the MJD provides a small advantage over JD in terms of clustering but not much difference in terms of cellular prevalence error over the simulated data. Figure S8 shows the application of the MJD on real datasets. There is a marked improvement for the TNBC Xenograft dataset and modest gains in the HGSOvCa dataset. Finally to check the effects of misspecifying the ADO rate in the MJD, we simulated 10 datasets with a true ADO rate of 0.3 and ran ddClone multiple times on this dataset, each time using the MJD with a different ADO rate (Figure S9). This results suggest that MJD is robust to misspecification of the ADO rate.

3 Simulation

3.1 Simulating genotypes

Here we introduce our simulation scheme. We first use the Generalized Dollo (GD) model to simulate cell genotypes. We then use these cell genotypes to simulate the bulk data. A binarized version of the cell genotypes is used to inform our prior in our method, while the bulk data constitutes the main input to our model and the competing methods. Figure S1 shows the high-level data simulation workflow.

We used a variation of the Stochastic Dollo (SD) model, called Generalized Dollo Model (GD) to sim-

ulate synthetic data accounting for both SNVs and CNVs. SD is a stochastic process that models evolution of binary features (in our case, point mutations) along a phylogenetic tree. A feature could only be gained on one point on the tree, and could be lost multiple times on different branches, but when lost, it cannot be regained [7].

A limitation of SD is that it is restricted to binary features. For instance, it can only model presence and absence of a mutation at a certain genomic locus.

Multi State Stochastic Dollo (MSSD) model [7] relaxes this restriction by expanding the *present feature* state and allowing transition within this expanded state space. For example, MSSD allows transition and transversion point mutations in addition to deletion.

MSSD can only model evolution after a SNV has happened. This is not a correct assumption when modelling copy number variation events where we would like to be able to account for copy number changes before a point mutation has happened.

To resolve this problem, in addition to expanding the *present feature*, we also expand the *absence feature* and allow transition within these new states. This is the GD model. Once the system *gains the feature*, that is, it transits into the *present features* state subspace, it can make transitions within this subspace, but cannot go back to the previous state. Figure S11 illustrates SD, MSSD, and GD side by side on a specific phylogenetic tree for a particular genomic locus.

Algorithm S1 Simulating From Generalized Dollo Model

```
procedure SIMULATEGENERALIZEDDOLLO (τ, μ, Q_ABOVE, Q_BELOW)
    for i in 1 : N do
        Simulate SNV edge e_SNV ~ ν(τ, μ)
        Simulate SNV point p_SNV ~ Uniform[0, |e_SNV|] on e_SNV.
        Simulate state of CTMC at the root node, H_i(Ω) ~ Categorical(u_i)
        aboveStates ← sampleTreeCTMC(τ_{-p_SNV}, Q_above)
        belowStates ← sampleTreeCTMC(τ_{p_SNV}, Q_below)
        allStates ← allStates ∪ combine(aboveStates, belowStates)
    return allStates
```

where

$$X \sim \nu(\tau, \mu) \Leftrightarrow p(X = x) = \frac{1}{||\tau|| + 1/\mu} \times \begin{cases} |x| & \text{if } x \neq \Omega \\ 1/\mu & \text{otherwise} \end{cases} \tag{S9}$$

and sampleTreeCTMC(τ, Q) simulates along a phylogeny, a substitution CTMC and a substitution-deletion CTMC for rate matrices Q_1 and Q_2 respectively.

Since ν has a point mass μ on Ω, there is a non-zero probability that a SNV happens at the root and hence $\tau_{-p_{SNV}} = \emptyset$. In this case sampleTreeCTMC$(\emptyset, Q)$ returns $\emptyset$. If a SNV does not happen at the root, then with probability one there are genotypes in the sample that have no variant allele copy at that genomic locus.

This will give us the copy number of each genotype at each genomic locus. We summarize this into copy-number aware genotype matrix $\Delta^{CN} \in (\mathbb{N} \times \mathbb{N})^{M \times N}$. Each element of this matrix $\Delta^{\text{CN}}_{m,n}$ is a pair = (CN_R, CN_V) where CN_R and CN_V represent reference and variant allele copy numbers respectively at genomic locus n for the m-th genotype. The binary genotype matrix Δ comes from binarized Δ^{CN}. An element of Δ is equal to one if the second element of the corresponding element in Δ^{CN} is non-zero, and it is zero otherwise. Section 3.5 shows 10 datasets generated from the GD model. Each dataset has a phylogenetic tree and 10 genotypes each with 48 genomic loci.

GD model's setup

GD uses a Continuous-Time Markov Chain (CTMC) to simulate the evolution of genomic loci states along the paths of the phylogenetic tree. The state space of this CTMC consists of pairs $(c_1, c_2) \in \mathbb{N}_0 \times \mathbb{N}_0$ where $\mathbb{N}_0 = \mathbb{N} \cup \{0\}$ and c_1 and c_2 represent reference copy and variant copy numbers respectively. Rate matrix Q_1 controls the CTMC before the occurrence of the rare-event and rate matrix Q_2 controls the CTMC after the occurrence of the rare-event.

We design Q_1 and Q_2 such that a complete deletion, i.e., transitioning to state $(0, 0)$ is not possible. Q_1 only allows transition between states that have zero variant copy number. This simulates the behaviour of the system before a SNV happens. We assume once a mutation is lost, it cannot be recovered, and enforce this assumption in Q_2 by not allowing transition from states with zero variant copy number zero to states with non-zero variant copy numbers.

Simulating from the GD model

To simulate data for each genomic locus from the GD model on a phylogenetic tree, we sample a point on the tree by selecting a topological location according to Equation (S9), followed by continuous uniform

sampling on the branch if the location is not the most recent common ancestor. The sampled point is interpreted as the location at which the SNV occurred for the current locus. We call the subtree rooted at the sampled point, the below-subtree and the remaining part of the tree, the above-subtree. We simulate GD on the above-subtree to determine the copy number of the SNV point along with other point on this subtree. This accounts for evolution of the genotypes before a SNV has happened. We continue by simulating the GD on the below-subtree to determine the copy number of decedents of the SNV point. This accounts for evolution of the genotypes after the SNV occurs.

To continue we first establish some notation following [8]. We define a phylogeny denoted by τ to be a continuous set of points and $\mathcal{G}(\mathcal{L}, \mathcal{E})$ to be its topology where $\mathcal{L}$ are the leaves and $\mathcal{E}$ the edges. Let $H_i(\nu)$ denote the state of the CTMC for genomic locus i at point ν. For an edge $e \in \mathcal{E}$, let $|e|$ denote its length.

Let τ_{-x} be the tree pruned at point x, that is, the tree with subtree rooted at x, removed.

We write τ_x for the subtree of τ rooted at node x, and τ_{-x} the subtree pruned at node x, that is, the subtree with points in $\tau - \tau_x$. Let ρ be a normalized measure that assigns zero weights to absorbing states and equal weights to non-absorbing states. Then the state of the CTMC at the root $H_i(\Omega)$ is distributed according to a categorical distribution with parameter ρ.

Algorithm S1 shows the pseudo code to simulate from Generalized Dollo Model. As input we provide the SimulateGeneralizedDollo procedure with tree topology τ with M leaves and parameter μ, as well as rate matrices $Q_{\text{above}}, Q_{\text{below}}$.

3.2 Simulating single cell data

To simulate cells, we first sample observed prevalences $\Phi = \{\Phi_1^{\text{observed}}, \Phi_2^{\text{observed}}, ..., \Phi_M^{\text{observed}}\}$ for each genotype from a Dirichlet distribution $\Phi_{\text{observed}} \sim \text{Dir}(\lambda\Phi)$, where $\Phi = \{\Phi_1, \Phi_2, ..., \Phi_M\}$ are the true prevalences for genotypes 1 to M. We then simulate m cells from a multinomial distribution with parameters Φ_{observed}, i.e., $(n_1, n_2, ..., n_M) \sim \text{Mult}(\Phi_{\text{observed}})$ where n_i is the number of cells that have genotype i. This process is equivalent to sampling the cells from a Dirichlet-multinomial distribution, that is, $(n_1, n_2, ..., n_M) \sim \text{Dirichlet-multinomial}(\lambda\Phi)$. The larger the λ is, the closer are the two vectors Φ_{observed} and Φ. In fact as the value of λ grows, the Dirichlet-multinomial distribution progressively better approximates the Multinomial distribution. For each dataset, we represent the average error between true and observed prevalences by $e_\Phi = \frac{1}{M}\sum_1^M |\Phi_i - \Phi_i^{\text{observed}}|$, the average absolute difference between true and

observed genotype prevalences. We measure the discrepancy between the true and the observed prevalences by the number of absent genotypes in the samples of cells and by e_Φ, the average error between true and observed prevalences.

For $\lambda = 0.01$, on average only about 1 out of 10 genotypes are observed in the sampled cells and $e_\Phi = 0.17$. In contrast, when $\lambda = 1000$, on average, over 9 out of 10 genotypes are observed and observed prevalences closely resemble the true genotype prevalences ($e_\Phi = 0.008$).

3.3 Simulating bulk data

We use the generated cell genotypes Δ^{CN} from the GD model to simulate the bulk data. This bulk data serves as the input to the competing methods in this work. Our method additionally takes as input a binarized version of the cell genotype data to inform its prior over partitions of genomic loci.

For each genomic locus i, the simulated bulk data consists in (i) variant and total allele counts (b_i, d_i), (ii) major and minor copy numbers $\bar{\zeta}_1^i$ and $\bar{\zeta}_2^i$, and (iii) tumour cellularity t. We set $t = 1$ for simulated experiments in this work.

Let $\Phi = \Phi_{1:M}$ where Φ_m is the clonal prevalence for cell genotype m, that is, the fraction of cells in the tumour sample that have cell genotype m and M be the total number of cell genotypes. Then ϕ_i, the clonal prevalence of genomic locus i in our sample would be $\Phi.\Delta[, i]$ and $\phi = \Phi.\Delta$. In this work, we set $\Phi_m = \frac{m}{\sum_{j=1}^{M} j}$.

To generate bulk data at genomic locus i, we first simulate d_i, the total number of reads mapping to i from a Poisson distribution with parameter 10,000. We then use the CN of the most prevalent genotype from Δ^{CN} (here, it would be the M-th genotype) at locus i to set the major and minor CNs for the bulk. That is we set $\bar{\zeta}_1^i = \text{Maximum}(\Delta_{M,i}^{\text{CN}})$ and $\bar{\zeta}_2^i = \text{Minimum}(\Delta_{M,i}^{\text{CN}})$. To simulate the variant allele counts b_i we have to take into account the aggregate effect of all genotypes at locus i in Δ^{CN}. This means that the ψ_i-s will be slightly different from that introduced in the Methods section of the main text, that is, instead of containing normal, reference, and variant subpopulations $\psi_i = (g_N^i, g_R^i, g_V^i)$, it should contain normal, and all the genotypes from Δ^{CN}. With a slight abuse of notation, we denote this by $\psi_i^*(\Delta^{\text{CN}}) = \psi_i^* = (g_N^i, g_1^i, g_2^i, ..., g_M^i)$. We

also have to modify the definition of ξ to work with the new ψ^* as follows:

$$\xi^*(\psi_i^*, \Phi_i, t) = \frac{(1-t)\zeta(g_N)}{Z^*}\mu(g_N) + t\sum_{j=1}^{M}\frac{\Phi_j\zeta(g_m^i)}{Z^*}\mu(g_m^i) \tag{S10}$$

where Z^* is the appropriate updated normalizing constant. Finally, we have $b_i \sim$ Beta-Binomial$(d_i, \xi^*(\psi_i^*, \phi_i, t), s)$ where we set $s = 1000$. Algorithm S2 summarizes the bulk data simulation procedure:

Algorithm S2 Simulate Bulk Data

procedure SIMULATEBULKDATA($\Phi, \Delta^{\text{CN}}, s, t$)
  for i in $1 : N$ **do**
    $d \leftarrow d\cup$ ($d_i \sim$ Pois$(10,000)$)
    $\bar{\zeta}_1 \leftarrow \bar{\zeta}_1 \cup$ Maximum$(\Delta^{\text{CN}}_{M,i})$
    $\bar{\zeta}_2 \leftarrow \bar{\zeta}_2 \cup$ Minimum$(\Delta^{\text{CN}}_{M,i})$
    $b \leftarrow b\cup$ ($b_i \sim$ Beta-Binomial$(d_i, \xi^*(\psi_i^*, \Phi_i, t), s)$)
  return $\{d, b, \bar{\zeta}_1, \bar{\zeta}_2, t\}$

3.4 Parameters for synthetic data generation

We used the following setup to generate synthetic data:

```
t = 1
d = 10,000
s = 1000
```
$\mu = 1$
```
number of genotypes = 10
number of genomic loci = 48
Max Total Copy Number = 4
```

3.5 Simulated genotypes from the GD model

See Figures S13 to S22.

4 Triple-negative breast cancer xenograft data

To test our method over a real dataset, we used a subset of samples from a triple-negative breast cancer xenograft study [1] where breast cancer tissues from 55 patients were transplanted into highly immunodefi-

cient mice to generate 30 xenograft lines. Over 3 years, these lines were passaged up to 16 generations.

Whole genome sequencing was performed over a subsample of this cohort to identify candidate genomic positions. It was followed by deep targeted amplicon sequencing of between 100 to 300 SNV positions per sample. 210 cells from five timepoints that span two samples were chosen for single cell genotyping, and about 48 SNV positions were targeted for each timepoint.

The results were post-processed to remove all positions labeled as non-somatic. This was further summarized into constituent cell genotypes. A consensus phylogenetic tree was inferred using MrBayes [9]. Cells were grouped into clades consisting of high probability branching splits. For each clade a consensus cell genotype was derived by taking the most prevalent cell genotype at each genomic locus. Figure S23 shows the inferred cell genotype matrix Δ for each sample. In each timepoint, we only kept genomic loci that were shared between the bulk and single cell genotype data. Inferred cell genotypes from the triple-negative breast cancer xenograft single cell genotyping study is shown in Figure S23.

4.1 Establishing the benchmark

Since exact clustering configuration and cellular prevalences of the genomic loci in the real dataset is unknown, we used multi-sample PyClone's result over 11 timepoints from sample SA501 and 4 timepoints from sample SA494 as our benchmark (figure S26). PyClone in multi-sample mode borrows statistical strength across all timepoints to give better estimates of subclonal structure in individual timepoints.

The following timepoints were used for sample SA501:

```
SA501T, SA501X1A, SA501X2A,
SA501X2B,
SA501X3A, SA501X3B, SA501X4A, SA501X4B,
SA501X4C, SA501X4D, SA501X5A.
```

The following timepoints were used for sample SA494:

```
SA494X4, SA494X3, SA494X2, SA494T
```

PyClone was run for 100,000 iterations with a burn-in period of 50,000 iterations. The rest of the settings were identical to synthetic simulation experiments as in listing 9. Cellular prevalence estimates are summarized in Figure S24.

We ran our method along with competing methods over timepoints `SA501 X1, X2, X4`, and `SA494 T, X4` for which we had matching single genotype sequencing data. Figure 6 in the main text shows the performance of each method against the benchmark.

5 Discarded data and potential effects on inference

Table S4 shows the number of incorporated mutations in the real datasets. ddClone works on the set of mutations that are present both in bulk and single cell data. Therefore in this work, we used the intersection of mutations in bulk and single cell data. On average the TNBC Xenograft dataset [1] had 141 mutations sequenced in the bulk data and 49 mutations sequenced in the single cell data, 41 of which were shared by both sources and were used in our benchmarking. In the HGSOvCa [10] dataset, out of the 141 and 49 loci that were sequenced in the bulk and single cell data respectively (on average), 41 targeted the same mutation. Finally, in the acute lymphoblastic leukemia (ALL) dataset [11], on average, 45 loci were shared by the 46 and 45 loci that were targeted in the bulk and single cell experiments respectively.

All methods are limited in a way and have to use the type of data that is available to them. ddClone needs genomic loci shared by both bulk and single cell sequencing data. The discarded positions may carry information about extra clusters in which case we expect ddClone to report fewer clusters.

6 Sensitivity analysis

6.1 Sensitivity to presence of doublets and sampling distortion noise

The the main text, Fig. 3, we compare ddClone with a number of bulk only and single cell only methods under three noise regimes, namely: (i) ideal data with no ADO or doublets; (ii) data with moderate levels of sampling distortion, in presence of 30% doublet cells and an ADO rate of 30%, and finally (iii) data with higher levels of sampling distortion reflective of real data, with the same doublet and ADO rates to ii. In Figure S25 we investigate what happens in absence of doublets. As expected we observed an improvement in the performance of single cell only methods, but the ranking still does not change and ddClone outcompetes all the other methods.

6.2 Sensitivity to a and noise

Here we examine the effects of simultaneously varying a and introducing noise. In our first experiment, we added point noise. We simulated five datasets from the GD model with 10 genotypes over 48 genomic loci. For each dataset, we ran ddClone for 200 iterations 60 times. Each time we fixed the hyperparameters a, α and s to a different starting value and disabled hyperparameter resampling. For each dataset, we introduced point noise with specified probability p to the original genotype matrix, and input the filtered genotype matrix to our model. Results for this experiment are shown in Figure S31. It implies that in presence of noise, the model is more sensitive to higher values of decay function parameter a and as a increases, model performance declines.

We examined two genotype loss scenarios: one where only a single genotype is lost, and one where progressively more genotypes are missed. Results for the first scenario are in Figure S32. Five datasets identical to the point noise experiment were generated. For each dataset, we held out the specified genotype and input the remaining as the genotype matrix to our model (i.e., a matrix with 9 genotypes in our experiments).

In the second scenario, we progressively removed more genotypes. Figure S33 depicts these results. Except for genotype loss, the rest of experiment setup was identical to the first scenario. This result implies that the model is more sensitive to the value of the decay function parameter a than it is to genotype removal.

6.3 Sensitivity to the initial value of hyperparameter a

Figure S34 shows the result of running our model with different starting values for the hyperparameter a. In these experiments we disabled resampling of hyperparameters a, α, and s, and fixed them at their starting value. We simulated 10 datasets from the GD model with 5 genotypes over 48 genomic loci. We ran our model 170 times for each dataset, with different initial values for hyperparameters, each time for 200 iterations. Each box plot shows the respective performance index for runs with an identical initial value of a and different values for s and α, each for 5 datasets.

7 Data requirements for the method to function well

7.1 Bulk data

The bulk likelihood portion of the model is designed for analysis of deeply sequenced (coverage $> 100Ł$) mutations to identify and quantify clonal structure [3].

7.2 Single cell data - number of cells

ddClone's performance in terms of both clustering and cellular prevalence accuracy highly depends on the number of captured genotypes (see Figure 7 in the main text). Even in presence of half of the genotypes, ddClone compares favourably against the second best performing bulk method.

The number of captured genotypes depends on the distribution of the genotypes across the tumour. We have modelled this using the concentration parameter λ in the Dirichlet-multinomial distribution that scales the original prevalence parameters (see Figure 4 in the main text). For small values of λ, sampled cells are not a good representative of the underlying tumour structure. For instance, at $\lambda = 1.12$, 50 sampled cells on average capture only slightly over 4 out of 10 existing genotypes. In this situation, even sampling 1000 cells will on average capture about 5 out of 10 genotypes. On the other hand, for larger values of λ, the sampling procedure results in cells that more accurately represent the tumour. For example, at $\lambda = 10$, a sample of 50 cells, on average capture 8 genotype out of 10 existing genotypes. Sampling 1000 cells in this situation will on average reveal an extra genotype. In summary, our results suggests that ddClone will perform comparably well when at least half of the genotypes are recognized.

7.3 Single cell data - depth

The sequencing depth at each loci has to be large enough to enable SNV detection. This is dependent on a number of factors including the variant allele frequency at the target locus, the library preparation and amplification, and mutation calling pipeline. In this study we used the binomial exact test described in [12] that recommends a coverage of at least > 43 at each locus. Reference [13] has a number of recommendations on what coverage to use. Moreover ddClone is designed to work with genotypes, rather than raw single cell data and therefore we anticipate that the coverage requirements of the genotype inference procedure may also be important (see [14] for an up to date review of such algorithms). Moreover, we have shown that

ddClone is fairly robust to presence of point noise (e.g., false positive or false negative error in SNVs as in Fig. 6 and Fig.7 in the main text).

8 Convergence diagnostics

Following [3] to assess convergence of the MCMC chain for TNBC Xenograft samples SA501 and SA494, we ran 3 chains for 10,000 iterations with random seeds and visually inspected Posterior Similarity Matrices (PSM) to ensure similarity. Figure S39 shows the PSM for time point X4 in sample SA494. These experiments imply that the chains have converged.

9 Parameter setting in method comparison experiments

We ran PyClone version `0.12.3` for 10,000 iterations with a burn-in of 1000 and thinning of 1. Remaining parameters were set as follows:

Bulk data only methods

```
num_iters:10,000
base_measure alpha=1
base_measure_ beta=1
concentration=1
prior shape = 1
prior rate = 0.001
density = pyclone_beta_binomial
beta_binomial_precison value = 1000
beta_binomial_prior shape = 1
beta_binomial_prior rate = 1
beta_binomial_precison proposal precision = 0.01
tumour_content = 1
error_rate = 0.001
```

We used Clomial version `1.4.0` and provided it with the correct number of clusters via its `c`. Remaining arguments were set as follows:

maxIt = 20

binomTryNum=10

c = True Number of Clusters

We note that for the simulation studies, when provided with the correct number of clusters, Clomial converged in no data points. therefore we set its `c` parameter to half of the gold standard one.

We downloaded PhyloWGS with git tag `smchet1-31-g57294e3` and used it with the default settings for the following parameters:

—mcmc–samples = 2500

—mh–iterations = 5000

Since this version of PhyloWGS did not output clonal frequencies, we edited the source code to extract these values. Furthermore, to simplify comparison with other methods, we provided PhyloWGS with an empty copy number file.

Single cell data only methods

SCITE

We downloaded latest version of SCITE from its GitHub repository

(hash = `512c8b84ddd2ff5632d1c9f310c38fbc6b109bc6`). It outputs a mutation tree. To compute a clustering of mutations, we first collapse the nodes in this mutation tree. Briefly, we traverse the tree in a level order (breadth-first search), and merge every node with degree less than 2 with its parent node. Nodes with degree 3 or more are left unmerged (we assigned them either to a single cluster or to separate clusters, depending on which results in a higher V-measure). This results in a new tree with potentially fewer nodes. Every node in this new tree comprises a new cluster. In this study we considered 5 maximum likelihood trees, calculated clustering and cellular prevalence estimates on all of them, evaluated them, and reported the one with the highest score.

We report the proportion of cells that are descendants of a particular mutation as its cellular prevalence. SCITE has the option to attach the cells at a node in the mutation tree it estimates. To count the number of cells that harbour a mutation, we recursively consider that mutation and all its descendant mutation nodes.

To run SCITE, we set the option `-a` to attach single cells to mutations. The option `-ad` accepts two

parameters, (i) the rate of missed heterozygous mutations, and (ii) the rate of heterozygous mutations called as homozygous mutations. We designate them as `ado.1` and `ado.2` respectively. For simulated studies, we set ado.1 to the true value of ADO rate. In the simulations studies, some of the error types do not exists, but setting them to zero caused SCITE to crash. In such scenarios, we set those parameters to a minimum value to let SCITE run:

```
fd = 1e-4/1000000
cc = 1e-4/1000000
ad.2 = 1e-4 * 100
```

If no estimates were available, we set these parameters as follows:

```
fd = .01
ad.1 = .25
ad.2 = .25
cc = .01
```

The rest of the parameters are as follows:

```
-i = 1
-l = 900000      // # of MCMC iterations
-max_treelist_size = 5
-e = 0.01        // learning error rate
```

OncoNEM

We used OncoNEM version `1.0`. Along with the a clonal lineage tree, it outputs an occurrence parameter Θ, the posterior probability of a mutation to have occurred in a subpopulation (clone). We assign a mutation to a clone with maximum probability. This constitutes OncoNEM's clustering prediction.

To infer mutation prevalences, we use the posterior mutation-cell matrix, `p_mut`. We call it's transpose p^t_{must}. Each element of this matrix, $p^t_{\text{must}}(i, j)$ indicates the posterior probability of a mutation i being present in the cell j. For each row i of this matrix, we define $t^i_{treshold}$ as the average of the elements in the row, that is $t^i_{treshold} = \sum_{j=1}^{M} p^t_{\text{mus}}(i, j)$. We then convert p^t_{must} into a binary cell-mutation matrix $G_{OncoNEM}$ by

assigning $G_{OncoNEM}(i,j) = 1$ if $p^t_{\text{mus}}(i,j) > t^i_{treshold}$ and $G_{OncoNEM}(i,j) = 0$ otherwise. We define OncoNEM's mutation cellular prevalence as the sum of columns of $G_{OncoNEM}$.

To run OncoNEM we followed the recommended steps in the OncoNEM's R-package Vignette. We set the initial search range for the false positive and false negative values to $\{0.010, 0.028, 0.046, 0.064, 0.082, 0.100\}$ The rest of the parameters are as follows:

```
epsilon = 5
delta = 25
checkMax = 10000
```

References

[1] Eirew P, Steif A, Khattra J, Ha G, Yap D, Farahani H, et al. Dynamics of genomic clones in breast cancer patient xenografts at single-cell resolution. Nature. 2014;.

[2] Shah SP, Roth A, Goya R, Oloumi A, Ha G, Zhao Y, et al. The clonal and mutational evolution spectrum of primary triple-negative breast cancers. Nature. 2012;486(7403):395–399.

[3] Roth A, Khattra J, Yap D, Wan A, Laks E, Biele J, et al. PyClone: statistical inference of clonal population structure in cancer. Nat Meth. 2014 04;11(4):396–398. Available from: `http://dx.doi.org/10.1038/nmeth.2883`.

[4] Blei DM, Frazier PI. Distance dependent Chinese restaurant processes. The Journal of Machine Learning Research. 2011;12:2461–2488.

[5] Ritter C, Tanner MA. Facilitating the Gibbs Sampler: The Gibbs Stopper and the Griddy-Gibbs Sampler. Journal of the American Statistical Association. 1992;87(419):861–868. Available from: `http://www.tandfonline.com/doi/abs/10.1080/01621459.1992.10475289`.

[6] Neal RM. Markov Chain Sampling Methods for Dirichlet Process Mixture Models. Journal of Computational and Graphical Statistics. 2000;9(2):249–265. Available from: `http://amstat.tandfonline.com/doi/abs/10.1080/10618600.2000.10474879`.

[7] Alekseyenko AV, Lee CJ, Suchard MA. Wagner and Dollo: a stochastic duet by composing two parsimonious solos. Systematic biology. 2008;57(5):772–784.

[8] Bouchard-Côté A, Jordan MI. Evolutionary inference via the Poisson indel process. Proceedings of the National Academy of Sciences. 2013;10.1073/pnas.1220450110.

[9] Ronquist F, Teslenko M, van der Mark P, Ayres DL, Darling A, Höhna S, et al. MrBayes 3.2: efficient Bayesian phylogenetic inference and model choice across a large model space. Systematic biology. 2012;61(3):539–542.

[10] McPherson A, Roth A, Laks E, Masud T, Bashashati A, Zhang AW, et al. Divergent modes of clonal spread and intraperitoneal mixing in high-grade serous ovarian cancer. Nat Genet. 2016 07;48(7):758–767. Available from: http://dx.doi.org/10.1038/ng.3573.

[11] Gawad C, Koh W, Quake SR. Dissecting the clonal origins of childhood acute lymphoblastic leukemia by single-cell genomics. Proceedings of the National Academy of Sciences of the United States of America. 2014 Dec;111(50):17947–17952. Available from: http://www.ncbi.nlm.nih.gov/pubmed/25425670.

[12] Shah SP, Morin RD, Khattra J, Prentice L, Pugh T, Burleigh A, et al. Mutational evolution in a lobular breast tumour profiled at single nucleotide resolution. Nature. 2009 10;461(7265):809–813. Available from: http://dx.doi.org/10.1038/nature08489.

[13] Zafar H, Wang Y, Nakhleh L, Navin N, Chen K. Monovar: single-nucleotide variant detection in single cells. Nat Meth. 2016 04;advance online publication:–. Available from: http://dx.doi.org/10.1038/nmeth.3835.

[14] Navin NE, Chen K. Genotyping tumor clones from single-cell data. Nat Meth. 2016 07;13(7):555–556. Available from: http://dx.doi.org/10.1038/nmeth.3903.

Table S4: Breakdown of the number incorporated mutations real datasets for benchmarking

	dataSet	SampleID/PatientID	DataPoint	nBulkMuts	nSingleCellMuts	nCommonMuts	nUnionM
1	ALL	P1		20	19	19	[illegible]
2	ALL	P2		16	15	15	[illegible]
3	ALL	P3		49	48	48	[illegible]
4	ALL	P4		78	77	77	[illegible]
5	ALL	P5		105	104	104	[illegible]
6	ALL	P6		10	9	9	[illegible]
7	HGSOvCa	P2	Om1	140	43	37	[illegible]
8	HGSOvCa	P2	Om2	140	43	37	[illegible]
9	HGSOvCa	P2	ROv1	140	43	37	[illegible]
10	HGSOvCa	P2	ROv2	140	43	37	[illegible]
11	HGSOvCa	P3	Adnx1	211	84	60	[illegible]
12	HGSOvCa	P3	Om1	211	84	60	[illegible]
13	HGSOvCa	P3	ROv1	211	84	60	[illegible]
14	HGSOvCa	P3	ROv2	211	84	60	[illegible]
15	HGSOvCa	P9	LOv1	183	43	36	[illegible]
16	HGSOvCa	P9	LOv2	183	43	36	[illegible]
17	HGSOvCa	P9	Om1	183	43	36	[illegible]
18	HGSOvCa	P9	Om2	183	43	36	[illegible]
19	HGSOvCa	P9	ROv1	183	43	36	[illegible]
20	TNBC Xenograft	SA501	X1	177	55	45	[illegible]
21	TNBC Xenograft	SA501	X2	177	55	45	[illegible]
22	TNBC Xenograft	SA501	X4	177	55	45	[illegible]
23	TNBC Xenograft	SA494	T	88	42	35	[illegible]
24	TNBC Xenograft	SA494	X4	88	42	35	[illegible]

The columns include DataSet, indicating one of the three real datasets used in this study, SampleID/PatientID, indicating which sample or patient the tumour sample is coming from, DataPoint, that is the TimePoint in case of the TNBC Xenograft study, and anatomical sample in case of the HGSOvCa study. nBulkMuts indicates the number of mutations in the bulk sequencing experiment, nSingleCellMuts is the number of mutations in the single cell sequencing experiments. nCommonMuts indicates mutations that were shared in both bulk and single cell sequencing data and thus were used in this study and nUnionMuts indicates the total number of genomic loci targeted by either bulk or single cell sequencing experiments.
